# Rhodium-Catalyzed Reductive Carbonylative Cyclization of Aryl Alkynes with Hydrosilanes via C–H Activation to Access Silyl-Substituted Indanones

**DOI:** 10.1021/acs.orglett.6c00345

**Published:** 2026-03-11

**Authors:** Fengxiang Zhu, Mengdi Zhou, Xiao-Feng Wu

**Affiliations:** † School of Chemistry and Chemical Engineering, 12441 Shanxi University, Taiyuan 030006, China; ‡ Dalian National Laboratory for Clean Energy, Dalian Institute of Chemical Physics, Chinese Academy of Sciences, Dalian 116023, China; § Leibniz-Institut für Katalyse e.V., Albert-Einstein-Straße 29a, Rostock 18059, Germany

## Abstract

A rhodium-catalyzed reductive carbonylative cyclization for the direct synthesis of 3-silyl-1-indanones from readily available aryl alkynes and hydrosilanes is described. This transformation concurrently introduces a silicon moiety and constructs the indanone core under an atmosphere of carbon monoxide, exhibiting high regioselectivity. The reaction features a broad substrate scope across a range of symmetrical and unsymmetrical alkynes as well as diverse hydrosilanes. Preliminary mechanistic studies support a pathway involving hydrosilylation, rate-limiting C–H activation, and carbonylation. This work provides a concise and practical route to valuable organosilicon scaffolds.

## Graphical abstract

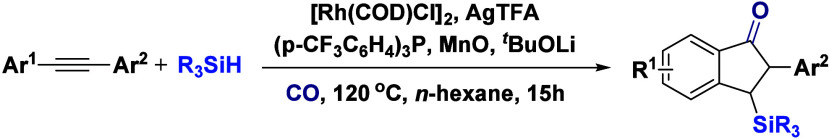

The indanone motif represents a fundamental structural scaffold in numerous bioactive natural products and pharmaceuticals (Scheme 1A).^1^ Consequently, the development of efficient and selective methods for their construction remains a persistent goal in synthetic chemistry. Classical approaches, such as intramolecular Friedel–Crafts acylation^2^ and the Heck reaction,^3^ often suffer from harsh conditions, limited functional group tolerance, and poor regiocontrol. Alternatively, transition-metal-catalyzed annulations of *ortho*-halogenated carbonyl compounds can afford indenols or indenones, which may be further transformed into indanones (Scheme 1B).^4,5^ However, such strategies usually require a stepwise sequence involving additional oxidation or reduction steps to convert intermediates into desired indanones. Transition-metal-catalyzed carbonylative annulations have emerged as a powerful alternative, offering convergent access to carbonyl-containing hetero- and carbocycles from simple unsaturated precursors.^6^ In particular, carbonylative C–H functionalization and cycloaddition processes mediated by transition metals have proven highly effective. Although elegant methods for synthesizing indanones via carbonylative cyclizations have been established,^7^ approaches to indanones bearing versatile functional handles—such as silyl groups—at specific ring positions remain significantly underdeveloped.

**Scheme 1:**
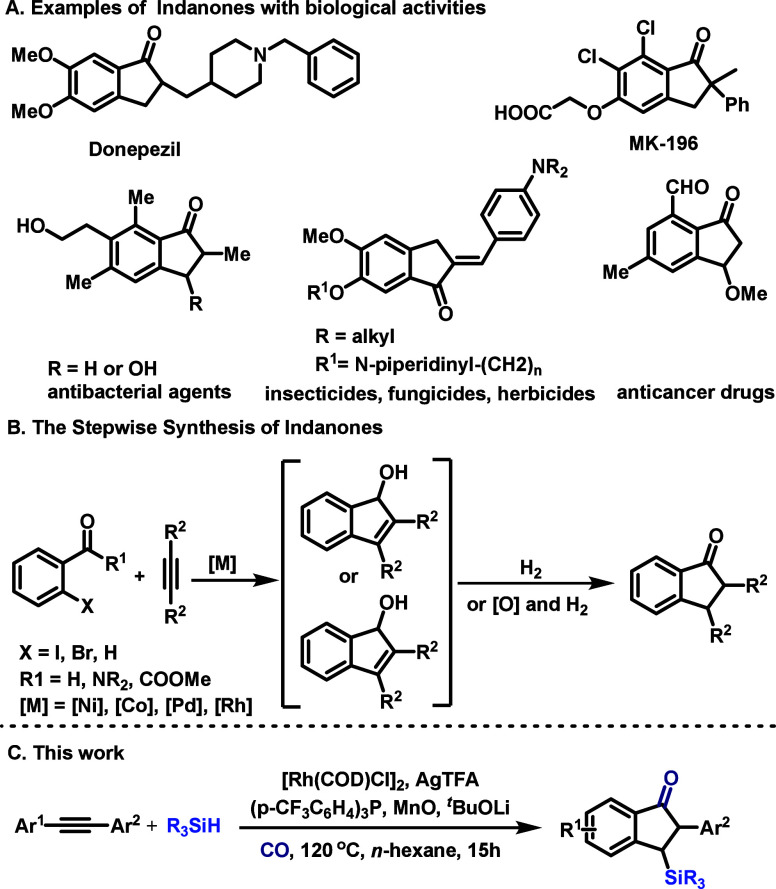
Examples of Bioactive Compounds and Reaction Design

The incorporation of silicon into organic frameworks profoundly alters their physicochemical and biological properties,^8^ making silylated compounds highly valuable in medicinal chemistry and materials science.^9^ Conventional strategies to access silylated indanones typically rely on multistep sequences involving prefunctionalized substrates or late-stage silicon installation,^10^ diminishing their overall efficiency and flexibility. A conceptually attractive approach would involve the direct assembly of the indanone core from readily available components while simultaneously introducing the silicon moiety.

Herein, we report the realization of this concept through a rhodium-catalyzed reductive carbonylative cyclization (Scheme 1C). This method directly converts readily available aryl alkynes and hydrosilanes into diverse 3-silyl-1-indanones with high regioselectivity under CO pressure. The reaction demonstrates a broad substrate scope and excellent functional group tolerance. Mechanistic studies elucidate a unique pathway involving vinylsilane formation, followed by rate-limiting C–H activation.

With triethylsilane (**1a**) and 1,2-diphenylacetylene (**2a**) as the model substrates, we initiated our investigation to establish this catalytic system (Table 1). Initial studies identified a promising combination of [Rh­(COD)­Cl]_2_ (3.5 mol %), tris­(*p*-(trifluoromethyl)­phenyl)­phosphine (L1, 15 mol %), silver trifluoroacetate (AgTFA, 20 mol %), lithium *tert*-butoxide (^*t*^BuOLi, 2.0 equiv), and manganese­(II) oxide (MnO, 2.0 equiv) in *n*-hexane under carbon monoxide (2 atm) at 120 °C, delivering desired 3-silylindanone **3aa** in 79% GC yield (Table 1, entry 1). Systematic optimization revealed the critical importance of each component. The reaction was completely dependent on rhodium precatalysis, and the specific precursor was crucial, as alternative complexes such as [Rh­(nbd)­Cl]_2_ and [Cp*RhCl_2_]_2_ proved significantly less effective (entries 2–4). The silver additive was essential, with its omission drastically reducing the yield to 25%; other silver salts (AgSbF_6_, AgBF_4_, and AgOTf) failed to match the performance of AgTFA, suggesting a beneficial role beyond halide abstraction of [Rh­(COD)­Cl]_2_ (entries 5 and 6). Ligand screening underscored a strong electronic dependence: electron-deficient (*p*-CF_3_C_6_H_4_)_3_P was found to be optimal. Its replacement with triphenylphosphine or other *para*-substituted triarylphosphines (electron-donating or electron-withdrawing) resulted in markedly lower yields, while bidentate phosphines (DPEPhos, XantPhos, DPPF, and DPPP) were largely ineffective (entries 7 and 8). The evaluation of oxidants identified MnO as uniquely effective, with MnO_2_ providing a moderate yield (64%) and other common oxidants (e.g., Cu­(OAc)_2_, quinones, and peroxides) proving unsuitable (entries 9 and 10). Finally, an examination of the base confirmed ^*t*^BuOLi as optimal, with other alkali metal *tert*-butoxides, carbonates, or phosphates affording inferior results, and no desired reaction occurred in the absence of base (entries 11 and 12). Notably, the yield of **3aa** decreased to 35% when the reaction was performed under 1 bar of CO.

**Table 1:**
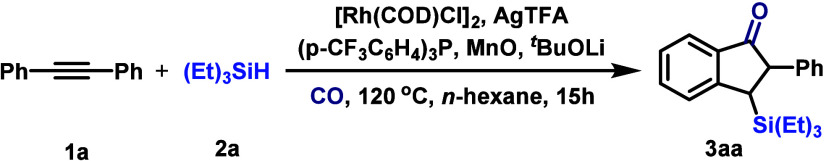
Screening of Optimal Reaction Conditions^a^

Entry	Variation from the standard conditions	Yield (%)
1	-	79
2	Without [Rh(COD)Cl]_2_	-
3	[Rh(nbd)Cl]_2_ instead of [Rh(COD)Cl]_2_	38
4	[Cp*RhCl_2_]_2_ instead of [Rh(COD)Cl]_2_	35
5	Without AgTFA	25
6	AgSbF_6_, AgBF_4_, AgBF_4_ instead of AgTFA	27, 33, 53
7	PPh_3_, (*p*-FC_6_H_4_)_3_P, (C_6_F_5_)_3_P, (*p*-MeC_6_H_4_)_3_P instead of (*p*-CF_3_C_6_H_4_)_3_P	31, 28, 11, 27
8^b^	DPEPhos, XantPhos, DPPF, DPPP instead of (*p*-CF_3_C_6_H_4_)_3_P	8, 9, 15, 17
9	MnO_2_, Cu(OAc)_2_ instead of MnO	64, 11
10	BQ, DDQ, K_2_S_2_O_8_, DTBP instead of MnO	12, trace, 14, 7
11	^t^BuONa, ^t^BuOK, MeOLi instead of ^t^BuOLi	43, 48, 35
12	Na_3_PO_4_, K_2_CO_3_, K_2_HPO_4_, Cs_2_CO_3_, Li_2_CO_3_ instead of ^t^BuOLi	65, 64, 63, 58, 32

a Reaction conditions: **1a** (0.1 mmol, 1.0 equiv), **2a** (0.2 mmol, 2 equiv), CO (2 bar), [Rh­(COD)­Cl]_2_ (3.5 mol %), AgTFA (20 mol %), (p-CF_3_C_6_H_4_)P (15 mol %), MnO (0.2 mmol, 2.0 equiv),^*t*^BuOLi (2.0 equiv), *n*-hexane (2 mL, super dry, water ≤ 30 ppm), stirred at 120 °C for 15 h, yields determined by GC analysis using hexadecane as an internal standard.

b Ligand (10 mol %). BQ: 1,2-benzoquinone. DDQ: 2,3-dichloro-5,6-dicyano-1,4-benzoquinone. DPEPhos: bis­(2-diphenyphosphinophenyl)­ether; XantPhos: 4,5-bis­(diphenylphosphino)-9,9-dimethylxanthene; DPPF: 1,1′-bis­(diphenylphosphino)­ferrocene; DPPP:1,3-bis­(diphenylphosphino)­propane.

With the optimized reaction conditions established, we next evaluated the scope and generality of this reductive carbonylative cyclization with respect to the alkyne component (Scheme 2). The transformation proved robust for symmetrical diarylacetylenes. While diphenylacetylene itself afforded product **3aa** in 79% yield, its derivatives bearing electron-donating *para*-substituents, such as methyl, *tert*-butyl, or methoxy groups, proceeded in even higher yields (**3ab–3ad**, 87–90%). Heteroaromatic systems were also compatible, as demonstrated by the efficient conversion of 1,2-*di*(thiophen-2-yl)­ethyne to **3ae** in 89% yield. The reaction tolerated *meta*-substitution, with 1,2-bis­(3-methylphenyl)­ethyne providing **3af** in 85% yield without observable regioselectivity issues. We then investigated the more challenging unsymmetrical diaryl acetylenes to probe the regioselectivity of the key *ortho* C–H bond activation and cyclization event.

**Scheme 2:**
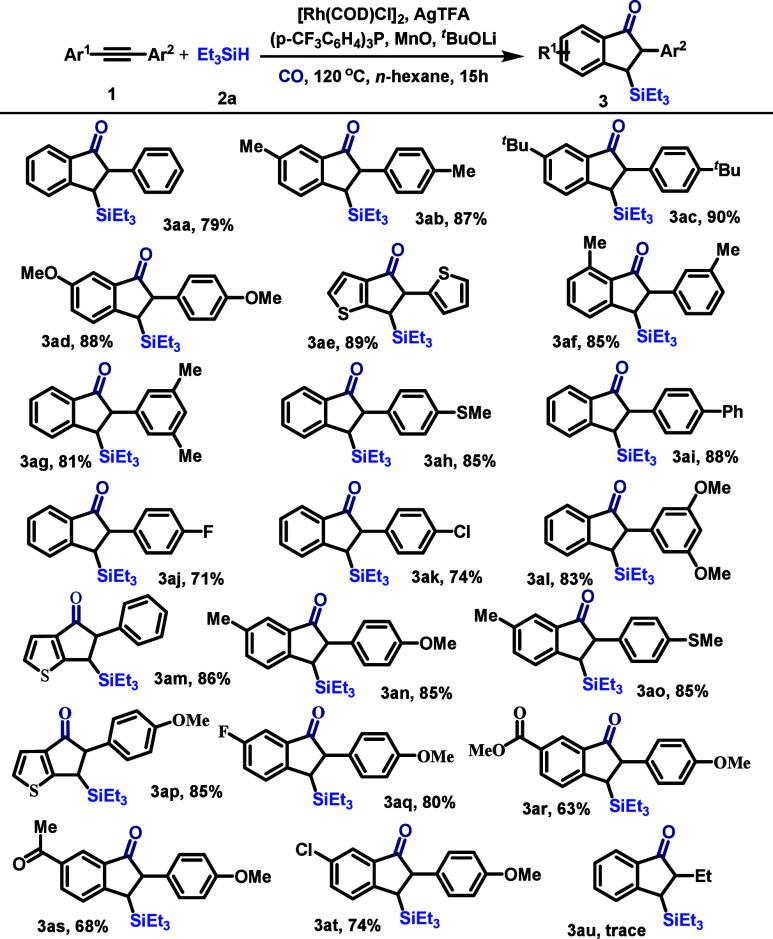
Variation of the Alkynes^a^

a Conditions: **1a** (0.1 mmol, 1.0 equiv), **2a** (0.2 mmol, 2 equiv), CO (2 bar), [Rh­(COD)­Cl]_2_ (3.5 mol %), AgTFA (20 mol %), (p-CF_3_C_6_H_4_)P (15 mol %), MnO (0.2 mmol, 2.0 equiv), ^*t*^BuOLi (2.0 equiv), *n*-hexane (2 mL, super dry, water ≤ 30 ppm), stirred at 120 °C for 15 h, isolated yields.

When one aryl ring was unsubstituted and the other bore substituents, cyclization occurred exclusively at the *ortho* position of the unsubstituted arene. This trend held for acetylenes with a disubstituted partner ring (e.g., 3,5-dimethyl, **3ag**, 81%; 3,5-dimethoxy, **3al**, 83%) or a *para*-monosubstituted one (**3ah–3ak**, 71–88%). Interestingly, for heteroaromatic derivative 2-(phenylethynyl)­thiophene, the cyclization took place on the thiophene ring, yielding **3am** in 86% yield, indicating the preferential activation of the heterocyclic C–H bond under the reaction conditions. However, the reaction failed when a furan or pyridine derivative of alkynes was tested. For acetylenes where both aryl rings bear a substituent, the reaction displayed a clear and instructive regioselectivity pattern. With substrates such as 1-methoxy-4-(*p*-tolylethynyl)­benzene and methyl­(4-(*p*-tolylethynyl)­phenyl)­sulfane, cyclization occurred selectively on the *p*-tolyl ring rather than on the *para*-methoxyphenyl or methylthiophenyl ring (**3an** and **3ao**, both 85%). This preference was systematically confirmed by using a *para*-methoxyphenyl group paired against a differentially substituted phenyl ring. In all cases examined—whether the opposing substituent was electron-donating, weakly withdrawing (e.g., COOMe, −COMe), or strongly withdrawing (e.g., −F, −Cl)—the *ortho* C–H activation and cyclization occurred consistently on the nonmethoxy-bearing arene, affording products **3aq–3at** in 63–80% yields. This trend extended to the heteroaromatic substrate 2-((4-methoxyphenyl)­ethynyl)­thiophene, where cyclization took place on the thiophene ring to furnish **3ap** in 85% yield. Notably, the reaction failed with the alkylaryl alkyne 1-phenylbut-1-yne, while various diaryl acetylenes proved effective. This stark contrast underscores a nonobvious but stringent structural requirement, suggesting that the presence of two (hetero)­aryl groups on the alkyne is crucial for productive engagement in the catalytic cycle, possibly due to their role in stabilizing key intermediates through π-interactions or by modulating the alkyne’s electron density.

Next, we examined the scope of hydrosilanes using diphenylacetylene as the standard alkyne partner (Scheme 3). The reaction exhibited excellent compatibility with a range of alkyl-substituted silanes. Trialkylsilanes such as ethyldimethylsilane and *tert*-butyldimethylsilane delivered products **3ba** and **3ca** in excellent yields of 92 and 90%, respectively. Diethylmethylsilane was also a competent substrate (**3da**, 83%). The protocol extended to aryl-substituted silanes, although yields were somewhat modulated by the steric and electronic properties of the arene. Benzyldimethylsilane provided **3ea** in 67% yield, while dimethylphenylsilane and its *para*-substituted derivatives with methyl, methoxy, fluoro, or chloro groups performed reliably, furnishing products **3fa–3ka** in 73–81% yields. Unfortunately, silanes containing alkoxy groups, such as diethoxy­(methyl)­silane **2l**, or those with multiple Si–H bonds, like diethylsilane **2m**, were ineffective under the standard conditions, which might be due to its stability issues. The reaction failed when triphenylsilane was tested.

**Scheme 3:**
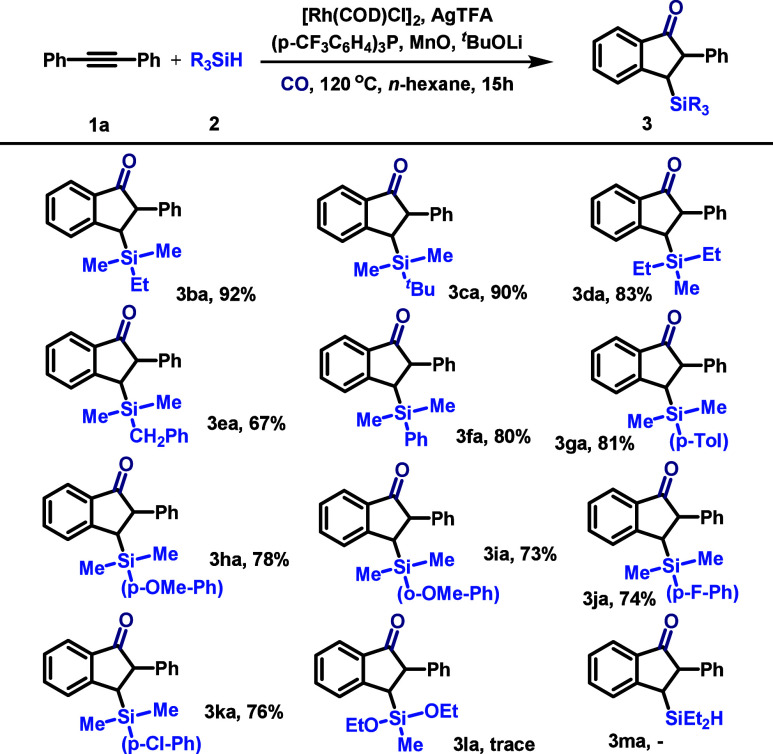
Variation of the Silanes^a^

a Conditions: **1a** (0.1 mmol, 1.0 equiv), **2** (0.2 mmol, 2 equiv), CO (2 bar), [Rh­(COD)­Cl]_2_ (3.5 mol %), AgTFA (20 mol %), (p-CF_3_C_6_H_4_)P (15 mol %), MnO (0.2 mmol, 2.0 equiv), ^*t*^BuOLi (2.0 equiv), *n*-hexane (2 mL, super dry, water ≤ 30 ppm), stirred at 120 °C for 15 h, isolated yields.

To demonstrate the practical utility of this catalytic protocol, a gram-scale reaction was performed using diphenylacetylene (**1a**, 2.0 mmol) and triethylsilane (4.0 mmol) under the optimized conditions. The desired 3-silylindanone **3aa** was isolated in 65% yield, confirming the scalability of this method (Scheme 4, step 1). For a better mechanistic understanding, a series of experiments were conducted (Scheme 4, steps 2 and 3). To probe the origin of the hydrogen atoms (H^1^/H^2^) in the product, we employed deuterium-labeled substrates. Subjecting [D_10_]-1,2-diphenylethyne and triethylsilane to the standard conditions afforded deuteriosilylation product [D_9_]-**3aa** in 61% yield with no deuterium incorporation at the H^1^/H^2^ positions (Scheme 4, step 2A). This result unambiguously excludes the *ortho* C–H bonds of the alkyne as the hydrogen source. Furthermore, using C_6_D_12_ as the solvent or adding D_2_O (5 μL) led to no deuterium incorporation in **3aa** (Scheme 4, step 2B), ruling out the solvent or adventitious water as hydrogen donors. Collectively, these labeling studies confirm that hydrogen atoms H1/H2 originate exclusively from the hydrosilane. A parallel kinetic isotope effect (KIE) experiment using an equimolar mixture of **1a** and [D10]-**1a** with triethylsilane revealed a kH/kD value of 3.0 (from a 3:1 product ratio), indicating that the *ortho* C–H bond cleavage is involved in the rate-determining step or a step preceding it (Scheme 4, step 2C). Several potential reaction intermediates, including a silyl-substituted inden-1-one **4** and stilbene **5**, were subjected to the standard conditions and failed to yield **3aa**, ruling out their roles as productive intermediates (Scheme 4, steps 2D and 2E). Interestingly, when the reaction of **1a** with triethylsilane was conducted in the absence of CO and MnO, vinylsilane (1,2-diphenylvinyl)­triethylsilane (**6**) was formed as the major product in 91% GC yield as a mixture of E/Z configuration. However, subjecting isolated **6** to the standard reaction conditions did not lead to the formation of indanone **3aa** (Scheme 4, step 2F). This result indicates that **6** is not a productive on-cycle intermediate.

**Scheme 4:**
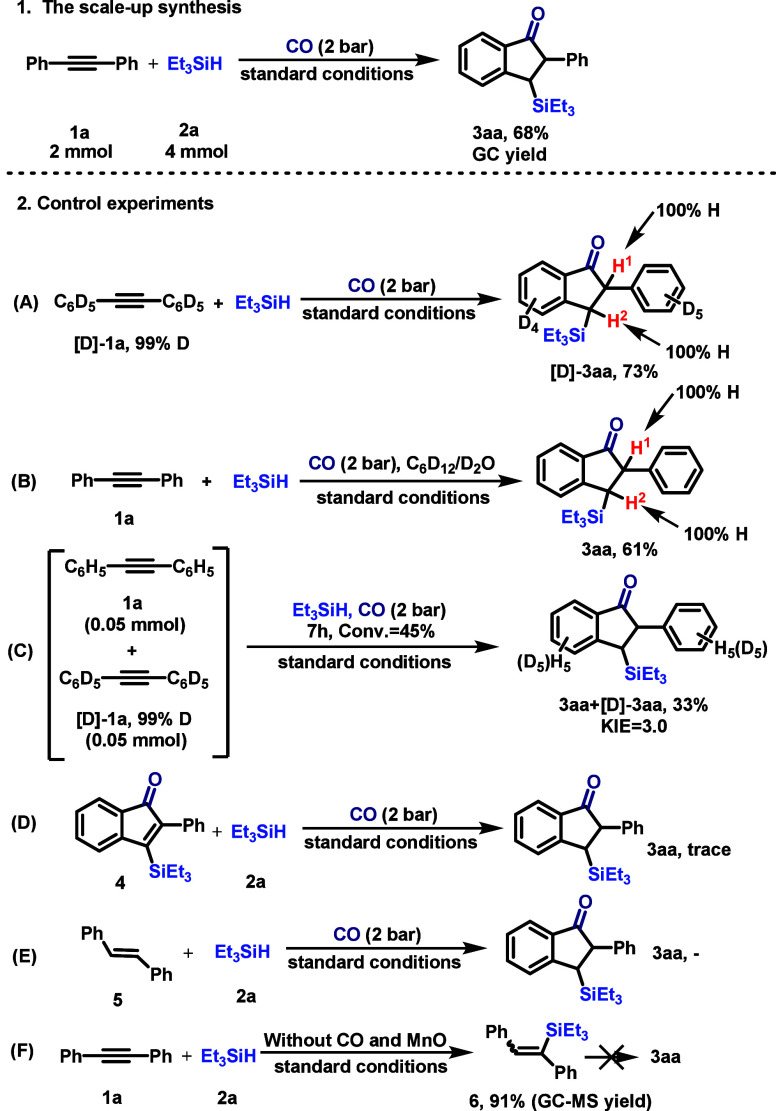
Synthetic Utility and Control Experiment

Based on the above mechanistic studies, a catalytic cycle is proposed (Scheme 5). The cycle is initiated by the oxidative addition of hydrosilane **2** to an active Rh­(I) species, generating hydridosilylrhodium­(III) intermediate **A**. Subsequent *cis*-hydrometalation (migratory insertion) of the alkyne into the Rh–H bond furnishes vinylrhodium species **B**. Assisted by the oxidant MnO and base ^*t*^BuOLi, intermediate **B** then undergoes an intramolecular, rate-determining *ortho* C–H activation to afford the key five-membered rhodacycle **C**. Following coordination and migratory insertion of carbon monoxide into the Rh–C­(aryl) bond, acylrhodium­(III) intermediate **D** is formed. Then reductive elimination and recoordination of RhH lead to complex **E**, which has further been transformed into alkoxyrhodium intermediate **F**. Subsequently, intermediate **F** reacts with a second equivalent of hydrosilane **2**. This step undergoes isomerization and liberates silyl-substituted indanone product **3** while regenerating the Rh^I^ catalyst, thereby closing the catalytic cycle.

**Scheme 5:**
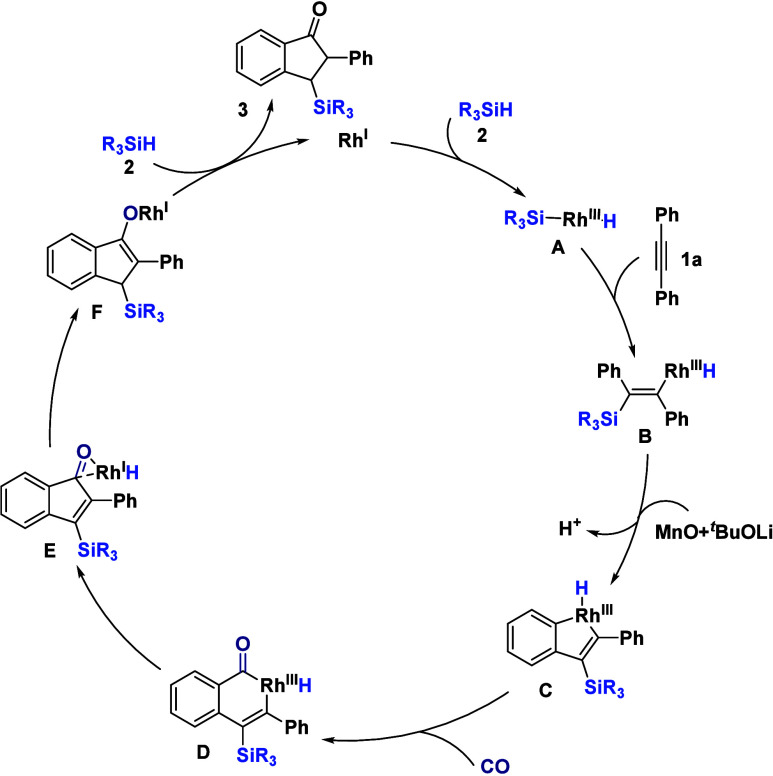
Proposed Mechanism

In summary, we have developed an efficient rhodium-catalyzed protocol for the direct assembly of 3-silyl-1-indanones from simple starting materials. This reductive carbonylative cyclization is operationally straightforward and scalable and tolerates a variety of functional groups, enabling rapid access to a diverse range of silicon-containing indanones that are challenging to synthesize by conventional means. Based on experimental observations, a plausible catalytic cycle has been proposed.

## Supplementary Material



## Data Availability

The data underlying this study are available in the published article and its Supporting Information.

## Notes

Experimental details, characterization data, and NMR spectra (PDF)
