# Supplementary material for: Rhodium-Catalyzed Reductive Carbonylative Cyclization of Aryl Alkynes with Hydrosilanes via C–H Activation to Access Silyl-Substituted Indanones
Source: Org Lett. 2026 Mar 11;28(11):3474–9. doi: 10.1021/acs.orglett.6c00345 (PMC13010350; doi:10.1021/acs.orglett.6c00345)

Electronic Supplementary Information For

Rhodium-Catalyzed Reductive Carbonylative Cyclization of Aryl Alkynes with Hydrosilanes via C–H Activation to Access Silyl-Substituted Indanones

Fengxiang Zhu,*[a] Mengdi Zhou[a] and Xiao-Feng Wu*[b,c]

[a] School of Chemistry and Chemical Engineering, Shanxi University, Taiyuan 030006 (China).

[b] Institution Dalian National Laboratory for Clean Energy, Dalian Institute of Chemical Physics, Chinese Academy of Sciences, Dalian 116023 (China)

[c] Leibniz-Institut für Katalyse e.V., Albert-Einstein-Straße 29a, Rostock 18059 (Germany)

E-mail: zfx201989@sxu.edu.cn (Fengxiang Zhu); xiao-feng.wu@catalysis.de (Xiao-Feng Wu).

Table of Content

1. General Methods S2
2. Typical reaction procedure for the synthesis of 3-silyl-1-indanones S2
3. Characterization data for products S3-S10
4. NMR Spectrum Copies S11-S44

1. General Methods

NMR spectra were recorded on Bruker Avance NEO 600 M and 400 M. Chemical shifts (ppm) are given relative to solvent: references for $CDCl_3$ were 7.26 ppm (^{1}H-NMR) and 77.0 ppm (^{13}C-NMR). ^{13}CNMR spectra were acquired on a broad band decoupled mode. Multiplets were assigned as s (singlet), d (doublet), t (triplet), dd (doublet of doublet), m (multiplet) and br. s (broad singlet). All measurements were carried out at room temperature unless otherwise stated. Gas chromatography analysis was performed on a Shimadzu 2014 instrument with an FID detector and HP-5 capillary column (polydimethylsiloxane with 5% phenyl groups, 30 m, 0.32 mm i.d., 0.25 µm film thickness) using N_2 as carrier gas. HRMS was obtained on a Bruker Daltonics Bio-TOF-Q mass spectrometer by the ESI method. The products were isolated from the reaction mixture by column chromatography on silica gel 60, 0.063-0.2 mm, 70-230 mesh. All reactions were carried out under air atmosphere. All the reagents were purchased from Heowns, Rhawn, and Laajoo chemical company.

2. Typical reaction procedure for the synthesis of 3-silyl-1-indanones:

General procedure: A 4 ml screw-cap vial was charged with diphenylacetylene (0.1 mmol, 1 equiv, 17.8 mg), triethylsilane (0.2 mmol, 2 equiv, 23.2 mg), (p-$CF_3C_6H_4)_3P$ (15 mol%, 7 mg), [Rh(COD)Cl]$_2$ (3.5 mol%, 1.7 mg), MnO (0.2 mmol, 14.2 mg), ^{t}BuOLi (0.2 mmol, 16 mg), AgTFA (20 mol%, 44.2 mg), *n*-hexane (2 mL, superdry, water≤30 ppm), and a stir bar. The vial was closed by a Teflon septum and a phenolic cap and connected to the atmosphere through a needle. Then the vial was fixed in an alloy plate and put into Paar 4560 series autoclave (300 mL). At room temperature, the autoclave is flushed with CO for three times, and 2 bar of CO was charged. The autoclave was placed on a heating plate equipped with magnetic stirring and an aluminum block. The reaction was heated at 120 °C for 15 hours. Afterwards, the autoclave was cooled to room temperature and the pressure carefully released. Upon completion, the reaction mixture was concentrated under vacuum. The residue was purified by silica gel column chromatography using a petroleum ether/AcOEt (120:1) as the eluent to give the corresponding products.

Experimental Procedures for Control Experiments

1. Gram-Scale Reaction

A mixture of diphenylacetylene **1a** (2.0 mmol, 1.0 equiv), triethylsilane **2a** (4.0 mmol, 2.0 equiv), [Rh(COD)Cl]$_2$ (3.5 mol%), AgTFA (20 mol%), (p-$CF_3C_6H_4)_3P$ (15 mol%), MnO (4.0 mmol, 2.0 equiv), and *t*-BuOLi (4.0 mmol, 2.0 equiv) in superdry *n*-hexane (20 mL, water ≤ 30 ppm) was placed in a 100 mL stainless steel autoclave. The autoclave was purged three times with CO, then pressurized to 2 bar CO and stirred at 120 °C for 15 h. After cooling to room temperature, the pressure was carefully released. The crude mixture was analyzed by GC using hexadecane as the internal standard, indicating a 68% GC yield (437.9 mg) of product **3aa**.

2. Deuterium Labeling Experiments

A. Reaction with [D_{10}]-Diphenylacetylene

[D_{10}]-Diphenylacetylene **1a**-d_{10} (0.1 mmol, 1.0 equiv) was subjected to the standard reaction conditions with triethylsilane 2a (0.2 mmol). After work-up, product [D_9]-**3aa** was isolated in 73% yield. ^{1}H NMR analysis revealed no deuterium incorporation at the H^1/H^2 positions, confirming that the ortho C–H bonds of the alkyne are not the hydrogen source.

B. Reaction with C_6D_{12} as Solvent or with D_2O Additive

The standard reaction of **1a** (0.1 mmol) and **2a** (0.2 mmol) was performed using: (i) C_6D_{12} (2 mL) as the solvent, and (ii) *n*-hexane (2 mL) as solvent with addition of D_2O (5 µL). In both cases, the isolated product **3aa** showed

no deuterium incorporation by ^{1}H NMR and GC-MS analysis, ruling out solvent or adventitious water as hydrogen donors.

C. Parallel Kinetic Isotope Effect Experiment

An equimolar mixture of **1a** (0.05 mmol) and [D_{10}]-**1a** (0.05 mmol) was reacted with triethylsilane **2a** (0.2 mmol) under the standard conditions for 7 h (conversion 45%). The product ratio of **3aa** to [D_9]-**3aa** was determined by ^{1}H NMR analysis to be 3:1, corresponding to a kH/kD value of 3.0. This indicates that ortho C–H bond cleavage is involved in the rate-determining step.

3. Control Experiments with Potential Intermediates

D. Reaction of Silyl-Substituted Inden-1-one 4

A mixture of silyl-substituted inden-1-one 4 (0.1 mmol), triethylsilane 2a (0.2 mmol), $[Rh(COD)Cl]_2$ (3.5 mol%), AgTFA (20 mol%), $(p\text{-}CF_3C_6H_4)_3P$ (15 mol%), MnO (0.2 mmol), and t-BuOLi (0.2 mmol) in *n*-hexane (2 mL, water ≤ 30 ppm) was stirred at 120 °C under 2 bar CO for 15 h. GC-MS analysis of the crude mixture showed no formation of **3aa**, with **4** remaining unreacted.

E. Reaction of Stilbene 5

The standard reaction conditions were applied to stilbene **5** (0.1 mmol) in place of diphenylacetylene **1a**. After 15 h, GC-MS analysis showed no formation of **3aa**, and **5** was recovered unchanged.

F. Reaction of Vinylsilane 6

Synthesis of Vinylsilane **6**: A mixture of diphenylacetylene **1a** (0.1 mmol), triethylsilane **2a** (0.2 mmol), $[Rh(COD)Cl]_2$ (3.5 mol%), and $(p\text{-}CF_3C_6H_4)_3P$ (15 mol%) in *n*-hexane (2 mL) was stirred at 120 °C under N_2 (without CO and MnO) for 15 h. GC-MS analysis confirmed formation of (1,2-diphenylvinyl)triethylsilane (**6**) in 91% GC yield.

Reaction of **6** under Standard Conditions: Isolated vinylsilane **6** (0.05 mmol) was subjected to the standard reaction conditions (with CO, AgTFA, MnO, base, etc.). After 15 h, GC-MS analysis showed no formation of **3aa**, and **6** was recovered unchanged, indicating that **6** is not a productive on-cycle intermediate.

3. Characterization data for products

2-phenyl-3-(triethylsilyl)-2,3-dihydro-1H-inden-1-one

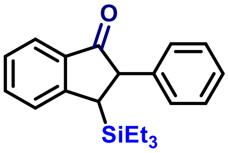

3aa

Purification by flash column chromatography (petroleum ether/EtOAc = 120:1). Yellow oily (25.5 mg, 79%).^{1}H NMR (600 MHz, $CDCl_3$) δ 7.79 (d, *J* = 8.0 Hz, 1H), 7.62 (t, *J* = 7.6 Hz, 1H), 7.45 (d, *J* = 7.6 Hz, 1H), 7.34 (t, *J* = 7.5 Hz, 1H), 7.27 (t, *J* = 9.6 Hz, 2H), 7.23 (d, *J* = 7.3 Hz, 1H), 7.14 (d, *J* = 8.7 Hz, 2H), 3.80 (d, *J* = 8.7 Hz, 1H), 3.06 (d, *J* = 6.2 Hz, 1H), 0.88 (t, *J* = 8.0 Hz, 9H), 0.64 (q, *J* = 8.0 Hz, 6H). ^{13}C NMR (151 MHz, $CDCl_3$) δ 206.5, 158.8, 140.8, 135.1, 134.9, 128.9, 127.2, 127.0, 126.3, 125.4, 125.2, 56.8, 36.8, 7.4, 2.5. GC-MS(EI-70eV): m/z (%) 322 (65), 87 (100), 191 (38), 115 (27), 59 (71). HRMS (ESI): calcd for $C_{21}H_{27}OSi^+$ $[M+H]^+$: 323.1826, found: 323.1831.

6-methyl-2-(p-tolyl)-3-(triethylsilyl)-2,3-dihydro-1H-inden-1-one

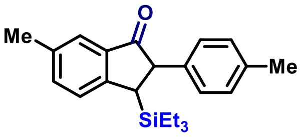

3ab

Purification by flash column chromatography (petroleum ether/EtOAc = 120:1). Yellow oily (30.5 mg, 87%). ^{1}H NMR (500 MHz, $CDCl_3$) δ 7.56 (s, 1H), 7.41 (d, *J* = 9.9 Hz, 1H), 7.30 (d, *J* = 7.9 Hz, 1H), 7.05 (d, *J* = 7.8 Hz, 2H), 6.99 (d, *J* = 8.1 Hz, 2H), 3.72 (d, *J* = 2.3 Hz, 1H), 2.94 (d, *J* = 2.4 Hz, 1H), 2.40 (s, 3H), 2.28 (s, 3H), 0.86 (t, *J* = 7.9 Hz, 9H), 0.60 (q, *J* = 7.6 Hz, 6H). ^{13}C N1MR (126 MHz, $CDCl_3$) δ 206.8, 156.1, 138.0, 136.5, 136.2, 136.0, 135.3, 129.6, 127.1, 125.1, 125.0, 56.7, 36.3, 21.0,

21.0, 7.5, 2.6. GC-MS(EI-70eV): m/z (%) 350 (94), 87 (100), 321 (27), 219 (67), 59 (82). HRMS (ESI): calcd for $C_{23}H_{31}OSi^+$ $[M+H]^+$: 351.2139, found: 351.2141.

6-(tert-butyl)-2-(4-(tert-butyl)phenyl)-3-(triethylsilyl)-2,3-dihydro-1H-inden-1-one

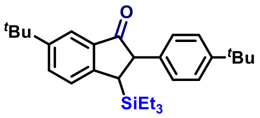

3ac

Purification by flash column chromatography (petroleum ether/EtOAc = 120:1). Yellow oily (39.1 mg, 90%). ^1H NMR (500 MHz, $CDCl_3$) δ 7.80 (s, 1H), 7.69 (d, J = 8.1 Hz, 1H), 7.38 (d, J = 8.2 Hz, 1H), 7.31 (d, J = 8.4 Hz, 2H), 7.09 (d, J = 8.4 Hz, 2H), 3.80 (d, J = 2.1 Hz, 1H), 3.02 (d, J = 2.6 Hz, 1H), 1.39 (s, 9H), 1.31 (s, 9H), 0.91 (t, J = 7.9 Hz, 9H), 0.65 (q, J = 8.2 Hz, 6H). ^{13}C NMR (126 MHz, $CDCl_3$) δ 207.1, 156.1, 149.6, 149.6, 137.9, 135.0, 132.7, 126.8, 125.8, 124.9, 121.3, 56.8, 36.1, 34.7, 34.4, 31.3, 7.4, 2.6. GC-MS(EI-70eV): m/z (%) 434 (82), 87 (100), 419 (39), 377 (38), 59 (53). HRMS (ESI): calcd for $C_{29}H_{43}OSi^+$ $[M+H]^+$: 435.3078, found: 435.3081.

6-methoxy-2-(4-methoxyphenyl)-3-(triethylsilyl)-2,3-dihydro-1H-inden-1-one

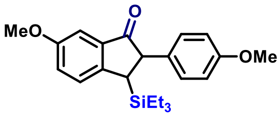

3ad

Purification by flash column chromatography (petroleum ether/EtOAc = 30:1). yellow solid (33.6 mg, 88%).^1H NMR (500 MHz, $CDCl_3$) δ 7.29 (s, 1H), 7.20 (d, J = 3.4 Hz, 2H), 7.04 (d, J = 8.7 Hz, 2H), 6.80 (d, J = 8.7 Hz, 2H), 3.83 (s, 3H), 3.75 (s, 3H), 3.73 (d, J = 2.1 Hz, 1H), 2.89 (d, J = 2.3 Hz, 1H), 0.86 (t, J = 7.9 Hz, 9H), 0.60 (q, J = 8.1 Hz, 6H). ^{13}C NMR (126 MHz, $CDCl_3$) δ 206.7, 158.6, 158.6, 151.5, 136.1, 133.0, 128.2, 126.1, 124.5, 114.3, 106.0, 56.6, 55.6, 55.2, 35.9, 7.4, 2.6. GC-MS(EI-70eV): m/z (%) 382 (87), 251 (100), 115 (19), 87 (78), 59 (63). HRMS (ESI): calcd for $C_{23}H_{30}NaO_3Si^+$ $[M+Na]^+$: 405.1856, found: 405.1857.

5-(thiophen-2-yl)-6-(triethylsilyl)-5,6-dihydro-4H-cyclopenta[b]thiophen-4-one

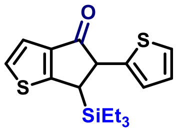

3ae

Purification by flash column chromatography (petroleum ether/EtOAc = 60:1). Yellow oily (29.7 mg, 89%). ^1H NMR (500 MHz, $CDCl_3$) δ 7.28 (d, J = 3.4 Hz, 2H), 7.20 (t, J = 3.8 Hz, 1H), 6.97 (d, J = 4.6 Hz, 2H), 4.32 (d, J = 2.4 Hz, 1H), 3.15 (d, J = 2.6 Hz, 1H), 0.94 (t, J = 7.9 Hz, 9H), 0.70 (q, J = 8.1 Hz, 6H). ^{13}C NMR (126 MHz, $CDCl_3$) δ 196.1, 174.5, 142.1, 141.6, 129.6, 127.0, 124.9, 124.2, 120.3, 57.1, 29.7, 7.3, 2.4. GC-MS(EI-70eV): m/z (%) 334 (50), 87 (100), 203 (20), 115 (28), 59 (48). HRMS (ESI): calcd for $C_{17}H_{22}NaOS_2Si^+$ $[M+Na]^+$: 357.0774, found: 357.0771.

7-methyl-2-(m-tolyl)-3-(triethylsilyl)-2,3-dihydro-1H-inden-1-one

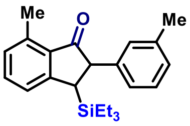

3af

Purification by flash column chromatography (petroleum ether/EtOAc = 120:1). Yellow oily (29.8 mg, 85%). ^1H NMR (500 MHz, $CDCl_3$) δ 7.66 (d, J = 7.8 Hz, 1H), 7.19 (s, 1H), 7.13 (t, J = 7.5 Hz, 2H), 7.00 (d, J = 6.7 Hz, 1H), 6.90 (d, J = 5.3 Hz, 2H), 3.71 (d, J = 2.3 Hz, 1H), 2.95 (d, J = 2.6 Hz, 1H), 2.47 (s, 3H), 2.27 (s, 3H), 0.86 (t, J = 7.9 Hz, 9H), 0.61 (q, J = 7.7 Hz, 6H). ^{13}C NMR (126 MHz, $CDCl_3$) δ 206.1, 159.4, 146.0, 141.0, 138.5, 133.0, 128.7, 127.9, 127.7, 127.6, 125.7, 125.0, 124.2, 57.0, 36.6, 22.4, 21.5, 7.4, 2.6. GC-MS(EI-70eV): m/z (%) 350 (19), 273 (100), 348 (52), 319 (34), 301 (42). HRMS (ESI): calcd for $C_{23}H_{30}NaOSi^+$ $[M+Na]^+$: 373.1958, found:373.1959.

2-(3,5-dimethylphenyl)-3-(triethylsilyl)-2,3-dihydro-1H-inden-1-one

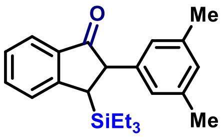

3ag

Purification by flash column chromatography (petroleum ether/EtOAc = 120:1). Yellow oily (28.4 mg, 81%). ^1H NMR (500 MHz, $CDCl_3$) δ 7.79 (d, J = 7.6 Hz, 1H), 7.62 (t, J = 7.6 Hz, 1H), 7.44 (d, J = 7.8 Hz, 1H), 7.34 (t, J = 7.2 Hz, 1H), 6.86 (s, 1H), 6.72 (s, 2H), 3.71 (d, J = 2.6 Hz, 1H), 3.02 (d, J = 2.3 Hz, 1H), 2.25 (s, 6H), 0.88 (t, J = 8.0 Hz, 9H), 0.63 (q, J = 7.9 Hz, 6H). ^{13}C NMR (126 MHz, $CDCl_3$) δ 206.8, 158.9, 140.6, 138.3, 135.2, 134.8, 128.7, 126.1, 125.4, 125.1, 125.0, 56.7, 36.7, 21.3, 7.4, 2.5. GC-MS(EI-70eV): m/z (%) 350 (20), 273 (100), 319 (42), 301 (34), 59 (31). HRMS (ESI): calcd for $C_{23}H_{31}OSi^+$ $[M+H]^+$: 351.2139, found: 351.2136.

2-(4-(methylthio)phenyl)-3-(triethylsilyl)-2,3-dihydro-1H-inden-1-one

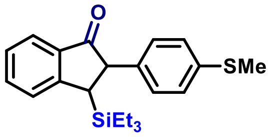

3ah

Purification by flash column chromatography (petroleum ether/EtOAc = 80:1). Red solid (31.3 mg, 85%). ^{1}H NMR (500 MHz, $CDCl_3$) δ 7.76 (d, *J* = 7.6 Hz, 1H), 7.59 (t, *J* = 7.6 Hz, 1H), 7.41 (d, *J* = 7.9 Hz, 1H), 7.32 (t, *J* = 7.4 Hz, 1H), 7.15 (d, *J* = 8.5 Hz, 2H), 7.04 (d, *J* = 8.5 Hz, 2H), 3.72 (d, *J* = 2.3 Hz, 1H), 2.99 (d, *J* = 2.4 Hz, 1H), 2.43 (s, 3H), 0.85 (t, *J* = 7.9 Hz, 9H), 0.61 (q, *J* = 7.9 Hz, 6H). ^{13}C NMR (126 MHz, $CDCl_3$) δ 206.3, 158.7, 137.7, 137.0, 135.0, 127.7, 127.4, 126.3, 125.4, 125.2, 56.3, 36.6, 16.1, 7.4, 2.6. GC-MS(EI-70eV): m/z (%) 368 (59), 87 (100), 292 (34), 237 (36), 59 (45). HRMS (ESI): calcd for $C_{22}H_{29}OSSi^+$ $[M+H]^+$: 369.1703, found: 369.1669.

2-([1,1'-biphenyl]-4-yl)-3-(triethylsilyl)-2,3-dihydro-1H-inden-1-one

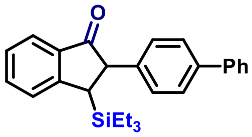

3ai

Purification by flash column chromatography (petroleum ether/EtOAc = 70:1). Yellow oily (35.0 mg, 88%).^{1}H NMR (500 MHz, $CDCl_3$) δ 7.54 (d, *J* = 8.2 Hz, 1H), 7.50 (d, *J* = 8.4 Hz, 1H), 7.44 (d, *J* = 6.9 Hz, 1H), 7.38 (t, *J* = 8.1 Hz, 2H), 7.35 (d, *J* = 2.8 Hz, 1H), 7.33 (d, *J* = 10.7 Hz, 1H), 7.30 (d, *J* = 7.9 Hz, 1H), 7.24 – 7.20 (m, 1H), 7.19 – 7.14 (m, 1H), 7.10 (d, *J* = 8.2 Hz, 2H), 7.06 (d, *J* = 6.9 Hz, 1H), 3.73 (d, *J* = 2.4 Hz, 1H), 2.98 (d, *J* = 2.4 Hz, 1H), 0.79 (t, *J* = 7.8 Hz, 9H), 0.55 (q, *J* = 8.1 Hz, 6H). ^{13}C NMR (126 MHz, $CDCl_3$) δ 206.4, 140.8, 139.9, 135.0, 134.0, 129.0, 128.8, 127.7, 127.3, 127.1, 126.4, 125.8, 125.5, 125.2, 123.2, 56.5, 36.7, 7.5, 2.6. GC-MS(EI-70eV): m/z (%) 398 (79), 87 (100), 369 (25), 267 (43), 59 (60). HRMS (ESI): calcd for $C_{27}H_{31}OSi^+$ $[M+H]^+$:399.2139, found: 399.2143.

2-(4-fluorophenyl)-3-(triethylsilyl)-2,3-dihydro-1H-inden-1-one

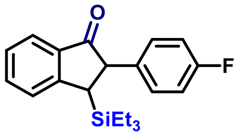

3aj

Purification by flash column chromatography (petroleum ether/EtOAc = 100:1). Yellow oily (24.1 mg, 71%). ^{1}H NMR (500 MHz, $CDCl_3$) δ 7.76 (d, *J* = 7.6 Hz, 1H), 7.60 (t, *J* = 7.6 Hz, 1H), 7.42 (d, *J* = 7.8 Hz, 1H), 7.33 (t, *J* = 7.4 Hz, 1H), 7.08 (d, *J* = 3.5 Hz, 2H), 6.94 (t, *J* = 8.6 Hz, 2H), 3.74 (d, *J* = 2.4 Hz, 1H), 2.98 (d, *J* = 2.4 Hz, 1H), 0.86 (t, *J* = 7.9 Hz, 9H), 0.61 (q, *J* = 7.9 Hz, 6H). ^{13}C NMR (126 MHz, $CDCl_3$) δ 206.2, 160.9(d, *J* = 239.4 Hz), 135.0(d, *J* = 37.8 Hz), 128.5(d, *J* = 12.6 Hz), 126.4, 125.4, 125.2, 115.8(d, *J* = 25.2 Hz), 55.9, 36.7, 7.4, 2.5. ^{19}F NMR (471 MHz, $CDCl_3$) δ -115.80. GC-MS(EI-70eV): m/z (%) 340 (76), 87 (100), 311 (38), 209 (41), 59 (67). HRMS (ESI): calcd for $C_{21}H_{25}FNaOSi^+$ $[M+Na]^+$: 363.1551, found: 363.1549.

2-(4-chlorophenyl)-3-(triethylsilyl)-2,3-dihydro-1H-inden-1-one

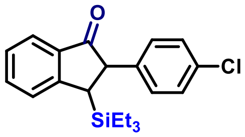

3ak

Purification by flash column chromatography (petroleum ether/EtOAc = 130:1). Yellow oily (26.3 mg, 74%). ^{1}H NMR (500 MHz, $CDCl_3$) δ 7.79 (d, *J* = 7.6 Hz, 1H), 7.64 (t, *J* = 7.6 Hz, 1H), 7.44 (d, *J* = 6.7 Hz, 1H), 7.35 (t, *J* = 7.5 Hz, 1H), 7.25 (d, *J* = 8.5 Hz, 2H), 7.08 (d, *J* = 8.4 Hz, 2H), 3.76 (d, *J* = 2.4 Hz, 1H), 3.00 (d, *J* = 2.4 Hz, 1H), 0.88 (t, *J* = 7.9 Hz, 9H), 0.64 (q, *J* = 8.1 Hz, 6H). ^{13}C NMR (126 MHz, $CDCl_3$) δ 205.9, 158.5, 139.2, 135.1, 134.8, 132.8, 129.1, 128.6, 126.4, 125.4, 125.3, 56.1, 36.6, 7.4, 2.5. GC-MS(EI-70eV): m/z (%) 356 (52), 87 (100), 225 (26), 151 (35), 59 (63). HRMS (ESI): calcd for $C_{21}H_{25}ClNaOSi^+$ $[M+Na]^+$: 379.1255, found: 379.1256.

2-(3,5-dimethoxyphenyl)-3-(triethylsilyl)-2,3-dihydro-1H-inden-1-one

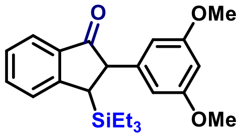

3al

Purification by flash column chromatography (petroleum ether/EtOAc = 30:1). Yellow oily5 (31.7 mg, 83%). ^{1}H NMR (500 MHz, $CDCl_3$) δ 7.78 (d, *J* = 7.8 Hz, 1H), 7.60 (t, *J* = 8.2 Hz, 1H), 7.42 (d, *J* = 7.6 Hz, 1H), 7.33 (t, *J* = 7.4 Hz, 1H), 6.33 (s, 1H), 6.30 (s, 2H), 3.74 (s, 6H), 3.71 (d, *J* = 2.1 Hz, 1H), 3.04 (d, *J* = 2.3 Hz, 1H), 0.88 (t, *J* = 7.9

Hz, 9H), 0.63 (q, *J* = 7.9 Hz, 6H). ^{13}C NMR (126 MHz, $CDCl_3$) δ 206.0, 161.0, 158.7, 143.0, 134.9, 126.2, 125.4, 125.2, 105.6, 98.5, 56.9, 55.3, 36.6, 7.4, 2.5. GC-MS(EI-70eV): m/z (%) 382 (88), 87 (100), 353 (59), 251 (38), 59 (84). HRMS (ESI): calcd for $C_{23}H_{30}NaO_3Si^+$ $[M+Na]^+$: 405.1856, found: 405.1851.

5-phenyl-6-(triethylsilyl)-5,6-dihydro-4H-cyclopenta[b]thiophen-4-one

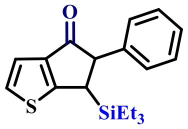

3am

Purification by flash column chromatography (petroleum ether/EtOAc = 60:1). Yellow oily (28.2 mg, 86%). ^{1}H NMR (500 MHz, $CDCl_3$) δ 7.34 (d, *J* = 7.0 Hz, 1H), 7.32 (d, *J* = 2.4 Hz, 1H), 7.30 (d, *J* = 1.9 Hz, 1H), 7.28 (t, *J* = 7.5 Hz, 1H), 7.22 (d, *J* = 1.8 Hz, 1H), 7.21 (t, *J* = 1.7 Hz, 1H), 7.20 (t, *J* = 1.4 Hz, 1H), 4.05 (d, *J* = 2.1 Hz, 1H), 3.07 (d, *J* = 2.9 Hz, 1H), 0.93 (t, *J* = 7.9 Hz, 9H), 0.71 (q, *J* = 8.1 Hz, 6H). ^{13}C NMR (126 MHz, $CDCl_3$) δ 198.0, 175.2, 142.8, 140.3, 129.5, 129.0, 127.4, 127.2, 120.2, 62.5, 36.7, 7.4, 2.5. GC-MS(EI-70eV): m/z (%) 328 (83), 87 (100), 197 (19), 115 (35), 59 (51). HRMS (ESI): calcd for $C_{19}H_{24}NaOSSi^+$ $[M+Na]^+$: 351.1209, found:351.1209.

2-(4-methoxyphenyl)-6-methyl-3-(triethylsilyl)-2,3-dihydro-1H-inden-1-one

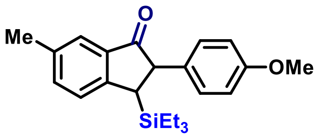

3an

Purification by flash column chromatography (petroleum ether/EtOAc = 60:1). Yellow oily (31.1 mg, 85%). ^{1}H NMR (500 MHz, $CDCl_3$) δ 7.56 (s, 1H), 7.41 (d, *J* = 7.9 Hz, 1H), 7.30 (d, *J* = 7.9 Hz, 1H), 7.04 (d, *J* = 8.7 Hz, 2H), 6.79 (d, *J* = 8.7 Hz, 2H), 3.75 (s, 3H), 3.71 (d, *J* = 2.3 Hz, 1H), 2.92 (d, *J* = 9.0 Hz, 1H), 2.39 (s, 3H), 0.86 (t, *J* = 7.9 Hz, 9H), 0.61 (q, *J* = 8.0 Hz, 6H). ^{13}C NMR (126 MHz, $CDCl_3$) δ 206.8, 158.6, 156.0, 136.2, 135.2, 133.1, 129.6, 128.2, 127.1, 125.1, 125.0, 114.3, 56.3, 55.2, 36.3, 21.0, 7.4, 2.6. GC-MS(EI-70eV): m/z (%) 366 (87), 87 (100), 235 (50), 115 (19), 59 (63). HRMS (ESI): calcd for $C_{23}H_{30}NaO_2Si^+$ $[M+Na]^+$: 389.1907, found: 389.1904.

6-methyl-2-(4-(methylthio) phenyl)-3-(triethylsilyl)-2,3-dihydro-1H-inden-1-one

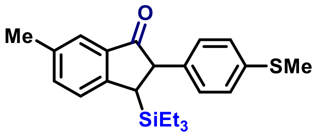

3ao

Purification by flash column chromatography (petroleum ether/EtOAc = 80:1). Red solid (32.5 mg, 85%). ^{1}H NMR (500 MHz, $CDCl_3$) δ 7.58 (s, 1H), 7.48 (d, *J* = 2.0 Hz, 1H), 7.32 (d, *J* = 8.2 Hz, 1H), 7.06 (d, *J* = 8.2 Hz, 2H), 7.00 (d, *J* = 8.1 Hz, 2H), 3.74 (d, *J* = 2.1 Hz, 1H), 2.94 (d, *J* = 2.6 Hz, 1H), 2.51 (s, 3H), 2.29 (s, 3H), 0.86 (d, *J* = 7.8 Hz, 9H), 0.61 (d, *J* = 8.1 Hz, 6H). ^{13}C NMR (126 MHz, $CDCl_3$) δ 206.1, 155.8, 137.7, 137.1, 136.7, 135.9, 133.9, 129.6, 127.0, 125.6, 121.6, 56.8, 36.4, 21.1, 15.9, 7.5, 2.6. GC-MS(EI-70eV): m/z (%) 382 (73), 87 (100), 306 (27), 251 (40), 59 (62). HRMS (ESI): calcd for $C_{23}H_{30}NaOSSi^+$ $[M+Na]^+$: 405.1679, found: 405.1685.

5-(4-methoxyphenyl)-6-(triethylsilyl)-5,6-dihydro-4H-cyclopenta[b]thiophen-4-one

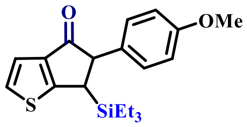

3ap

Purification by flash column chromatography (petroleum ether/EtOAc = 30:1). Yellow oily (30.4 mg, 85%). ^{1}H NMR (500 MHz, $CDCl_3$) δ 7.30 (d, *J* = 4.1 Hz, 1H), 7.21 (d, *J* = 5.2 Hz, 1H), 7.13 (d, *J* = 8.9 Hz, 2H), 6.88 (d, *J* = 8.7 Hz, 2H), 4.01 (d, *J* = 2.0 Hz, 1H), 3.82 (s, 3H), 3.03 (d, *J* = 2.3 Hz, 1H), 0.93 (t, *J* = 7.9 Hz, 9H), 0.70 (q, *J* = 8.2 Hz, 6H). ^{13}C NMR (126 MHz, $CDCl_3$) δ 198.4, 175.2, 158.8, 142.8, 132.3, 129.4, 128.4, 120.2, 114.4, 61.7, 55.3, 36.7, 7.4, 2.5. GC-MS(EI-70eV): m/z (%) 358 (77), 87 (100), 228 (18), 115 (27), 59 (62). HRMS (ESI): calcd for $C_{20}H_{26}NaO_2SSi^+$ $[M+Na]^+$: 381.1315, found: 381.1314.

6-fluoro-2-(4-methoxyphenyl)-3-(triethylsilyl)-2,3-dihydro-1H-inden-1-one

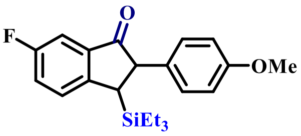

3aq

Purification by flash column chromatography (petroleum ether/EtOAc = 50:1). Yellow oily (29.6 mg, 80%). ^{1}H NMR (500 MHz, $CDCl_3$) δ 7.40 (d, J = 10.2 Hz, 1H), 7.31 (t, J = 9.2 Hz, 1H), 7.21 (d, J = 13.6 Hz, 1H), 7.08 (d, J = 9.5 Hz, 1H), 7.02 (d, J = 8.7 Hz, 1H), 6.96 (d, J = 8.7 Hz, 1H), 6.80 (d, J = 8.7 Hz, 1H), 3.89 (s, 1H), 3.76 (s, 3H), 2.90 (d, J = 31.3 Hz, 1H), 0.93 (t, J = 7.9 HZ, 9H), 0.60 (q, J = 7.9 Hz, 6H). ^{13}C NMR (126 MHz, $CDCl_3$) δ 206.2, 161.9(d, J = 239.4 Hz), 158.7, 136.5(d, J = 37.8 Hz), 132.4, 128.8(d, J = 18.8 Hz), 128.2, 126.1, 124.7, 115.7(d, J = 21.4 Hz), 114.4, 106.1, 56.7, 55.6, 36.1, 7.4, 2.6. ^{19}F NMR (471 MHz, $CDCl_3$) δ -115.84. GC-MS(EI-70eV): m/z (%) 370 (83), 87 (100), 239 (50), 115 (29), 59 (64). HRMS (ESI): calcd for $C_{22}H_{28}FO_2Si^+$ [M+H]$^+$: 371.1837, found: 371.1843.

methyl 2-(4-methoxyphenyl)-3-oxo-1-(triethylsilyl)-2,3-dihydro-1H-indene-5-carboxylate

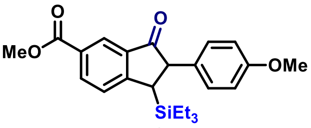

3ar

Purification by flash column chromatography (petroleum ether/EtOAc = 25:1). Yellow oily (25.8 mg, 63%). ^{1}H NMR (500 MHz, $CDCl_3$) δ 8.41 (s, 1H), 8.27 (d, J = 8.0 Hz, 1H), 7.47 (d, J = 8.1 Hz, 1H), 7.01 (d, J = 8.7 Hz, 2H), 6.80 (d, J = 8.7 Hz, 2H), 3.93 (s, 3H), 3.77 (d, J = 2.3 Hz, 1H), 3.76 (s, 3H), 3.05 (d, J = 2.6 Hz, 1H), 0.86 (t, J = 7.9 Hz, 9H), 0.62 (q, J = 8.2 Hz, 6H). ^{13}C NMR (126 MHz, $CDCl_3$) δ 205.7, 166.4, 158.8, 135.6, 135.2, 132.2, 128.7, 128.1, 126.7, 125.4, 124.4, 114.5, 56.3, 55.3, 52.3, 37.6, 7.4, 2.5. GC-MS(EI-70eV): m/z (%) 410 (87), 87 (100), 279 (27), 236 (29), 59 (77). HRMS (ESI): calcd for $C_{24}H_{31}O_4Si^+$ [M+H]$^+$: 411.1986, found: 411.1989.

6-acetyl-2-(4-methoxyphenyl)-3-(triethylsilyl)-2,3-dihydro-1H-inden-1-one

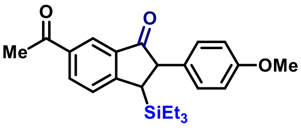

3as

Purification by flash column chromatography (petroleum ether/EtOAc = 25:1). Yellow oily (26.8 mg, 68%). ^{1}H NMR (500 MHz, $CDCl_3$) δ 8.27 (s, 1H), 8.25 (d, J = 8.1 Hz, 1H), 7.49 (d, J = 8.1 Hz, 1H), 7.02 (d, J = 8.9 Hz, 2H), 6.80 (d, J = 8.7 Hz, 2H), 3.79 (d, J = 2.3 Hz, 1H), 3.75 (s, 3H), 3.06 (d, J = 2.3 Hz, 1H), 2.63 (s, 3H), 0.86 (t, J = 7.9 Hz, 9H), 0.62 (q, J = 7.7 Hz, 6H). ^{13}C NMR (126 MHz, $CDCl_3$) δ 205.9, 197.0, 164.0, 158.8, 135.6, 135.1, 134.1, 132.1, 128.1, 125.7, 125.6, 114.5, 56.4, 55.3, 37.7, 26.6, 7.4, 2.6. GC-MS(EI-70eV):m/z (%)394 (98),87 (100),263 (30),115 (31),59 (68). HRMS (ESI): calcd for $C_{24}H_{31}O_3Si^+$ [M+H]$^+$: 395.2037, found: 395.2041.

6-chloro-2-(4-methoxyphenyl)-3-(triethylsilyl)-2,3-dihydro-1H-inden-1-one

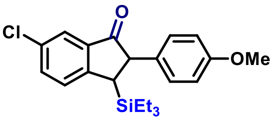

3at

Purification by flash column chromatography (petroleum ether/EtOAc = 30:1). Yellow oily (28.6 mg, 74%). ^{1}H NMR (500 MHz, $CDCl_3$) δ 7.7 (s, 1H), 7.5 (d, J = 8.2 Hz, 1H), 7.3 (d, J = 8.2 Hz, 1H), 7.0 (d, J = 8.9 Hz, 2H), 6.8 (d, J = 8.9 Hz, 2H), 3.8 (s, 3H), 3.7 (d, J = 2.3 Hz, 1H), 2.9 (d, J = 2.7 Hz, 1H), 0.9 (t, J = 7.9 Hz, 9H), 0.6 (q, J = 7.6 Hz, 6H). ^{13}C NMR (126 MHz, $CDCl_3$) δ 205.4, 158.8, 156.8, 136.5, 134.9, 132.5, 132.3, 128.2, 126.5, 124.8, 114.4, 56.4, 55.3, 36.4, 7.4, 2.5. GC-MS(EI-70eV): m/z (%) 386 (59), 87 (100), 255 (27), 115 (32), 59 (63). HRMS (ESI): calcd for $C_{22}H_{28}ClO_2Si^+$ [M+H]$^+$:387.1542, found: 387.1539.

3-(ethyldimethylsilyl)-2-phenyl-2,3-dihydro-1H-inden-1-one

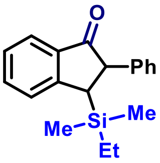

3ba

Purification by flash column chromatography (petroleum ether/EtOAc = 120:1). Yellow oily (26.9 mg, 92%). ^{1}H NMR (500 MHz, $CDCl_3$) δ 7.80 (d, J = 7.6 Hz, 1H), 7.62 (t, J = 7.6 Hz, 1H), 7.44 (d, J = 7.8 Hz, 1H), 7.35 (t, J = 7.4 Hz, 1H), 7.28 (t, J = 7.4 Hz, 2H), 7.23 (t, J = 6.6 Hz, 1H), 7.14 (d, J = 7.8 Hz, 2H), 3.76 (d, J = 2.4 Hz, 1H), 2.97 (d, J = 2.4 Hz, 1H), 0.91 (t, J = 7.9 Hz, 3H), 0.61 (q, J = 7.2 Hz, 2H), 0.05 (d, J = 7.2 Hz, 6H). ^{13}C NMR (126 MHz, $CDCl_3$)

δ 206.4, 158.4, 140.8, 135.1, 134.9, 128.9, 127.3, 127.0, 126.3, 125.4, 125.1, 56.8, 38.7, 7.2, 5.7, -4.9, -5.2. GC-MS(EI-70eV): m/z (%) 294 (52), 245 (100), 263 (46), 215 (11), 59 (24). HRMS (ESI): calcd for $C_{19}H_{23}OSi^+$ $[M+H]^+$: 295.1513, found: 295.1518.

3-(tert-butyldimethylsilyl)-2-phenyl-2,3-dihydro-1H-inden-1-one

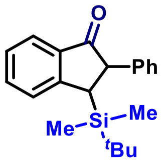

3ca

Purification by flash column chromatography (petroleum ether/EtOAc = 120:1). Yellow oily (29.0 mg, 90%). ^1H NMR (500 MHz, $CDCl_3$) δ 7.79 (d, *J* = 7.6 Hz, 1H), 7.62 (t, *J* = 8.2 Hz, 1H), 7.48 (d, *J* = 7.9 Hz, 1H), 7.35 (t, *J* = 7.5 Hz, 1H), 7.27 (t, *J* = 7.5 Hz, 2H), 7.22 (d, *J* = 7.3 Hz, 1H), 7.14 (d, *J* = 6.9 Hz, 2H), 3.92 (d, *J* = 2.0 Hz, 1H), 3.07 (d, *J* = 1.5 Hz, 1H), 0.89 (s, 9H), 0.13 (s, 3H), -0.02 (s, 3H). ^{13}C NMR (126 MHz, $CDCl_3$) δ 206.1, 158.9, 140.4, 135.0, 134.6, 128.9, 127.1, 127.0, 126.3, 126.0, 125.2, 57.6, 37.9, 27.3, 17.9, -5.5, -7.5. GC-MS(EI-70eV): m/z (%) 322 (57), 265 (100), 249 (18), 191 (88), 73 (84). HRMS (ESI): calcd for $C_{21}H_{27}OSi^+$ $[M+H]^+$: 323.1826, found: 323.1821.

3-(diethyl(methyl)silyl)-2-phenyl-2,3-dihydro-1H-inden-1-one

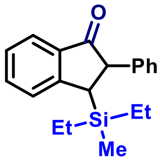

3da

Purification by flash column chromatography (petroleum ether/EtOAc = 120:1). Yellow oily (25.6 mg, 83%). ^1H NMR (500 MHz, $CDCl_3$) δ 7.80 (d, *J* = 7.6 Hz, 1H), 7.62 (t, *J* = 7.0 Hz, 1H), 7.44 (d, *J* = 7.6 Hz, 1H), 7.35 (t, *J* = 7.5 Hz, 1H), 7.28 (t, *J* = 7.3 Hz, 2H), 7.23 (d, *J* = 7.2 Hz, 1H), 7.14 (d, *J* = 7.0 Hz, 2H), 3.78 (d, *J* = 2.4 Hz, 1H), 3.02 (d, *J* = 2.3 Hz, 1H), 0.89 (t, *J* = 8.0 Hz, 6H), 0.62 (q, *J* = 7.8 Hz, 4H), 0.04 (s, 3H). ^{13}C NMR (126 MHz, $CDCl_3$) δ 206.4, 158.5, 140.8, 135.1, 134.9, 128.9, 127.3, 127.0, 126.3, 125.4, 125.1, 56.8, 37.7, 7.3, 4.2, 3.8, -7.1. GC-MS(EI-70eV): m/z (%) 308 (93), 73 (100), 279 (41), 191 (70), 101 (32). HRMS (ESI): calcd for $C_{20}H_{25}OSi^+$ $[M+H]^+$: 309.1669, found: 309.1671.

3-(benzyldimethylsilyl)-2-phenyl-2,3-dihydro-1H-inden-1-one

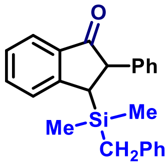

3ea

Purification by flash column chromatography (petroleum ether/EtOAc = 80:1). Yellow solid (23.9 mg, 67%). ^1H NMR (500 MHz, $CDCl_3$) δ 7.83 (d, *J* = 7.6 Hz, 1H), 7.65 (t, *J* = 7.6 Hz, 1H), 7.45 (d, *J* = 7.0 Hz, 1H), 7.39 (t, *J* = 7.5 Hz, 1H), 7.33 – 7.29 (m, 2H), 7.27 (d, *J* = 7.2 Hz, 1H), 7.18 (t, *J* = 7.5 Hz, 2H), 7.15 (d, *J* = 6.7 Hz, 2H), 7.09 (t, *J* = 7.4 Hz, 1H), 6.89 (d, *J* = 6.9 Hz, 2H), 3.77 (d, *J* = 2.4 Hz, 1H), 3.03 (d, *J* = 2.6 Hz, 1H), 2.21 (s, 2H), 0.09 (s, 3H), 0.03 (s, 3H). ^{13}C NMR (126 MHz, $CDCl_3$) δ 206.0, 157.8, 140.4, 138.6, 135.1, 135.0, 128.9, 128.4, 128.2, 127.4, 127.1, 126.5, 125.4, 125.2, 124.5, 56.8, 38.5, 24.0, -4.4, -4.9. GC-MS(EI-70eV): m/z (%) 356 (57), 265 (100), 191 (83), 149 (47), 121 (64). HRMS (ESI): calcd for $C_{24}H_{24}NaOSi^+$ $[M+Na]^+$: 379.1489, found: 379.1491.

3-(dimethyl(phenyl)silyl)-2-phenyl-2,3-dihydro-1H-inden-1-one

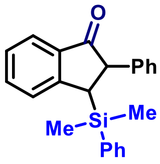

3fa

Purification by flash column chromatography (petroleum ether/EtOAc = 80:1). Yellow solid (27.4 mg, 80%). ^1H NMR (600 MHz, $CDCl_3$) δ 7.75 (d, *J* = 7.6 Hz, 1H), 7.52 (t, *J* = 7.5 Hz, 1H), 7.40 (d, *J* = 7.7 Hz, 3H), 7.34 (t, *J* = 6.5 Hz, 2H), 7.32 (d, *J* = 7.3 Hz, 1H), 7.24 – 7.17 (m, 3H), 7.14 (d, *J* = 7.8 Hz, 1H), 6.98 (d, *J* = 8.3 Hz, 2H), 3.73 (d, *J* = 2.4 Hz, 1H), 3.13 (d, *J* = 2.3 Hz, 1H), 0.32 (s, 6H). ^{13}C NMR (151 MHz, $CDCl_3$) δ 206.2, 157.7, 140.8, 136.0, 135.3, 134.8, 134.0, 129.8, 128.8, 128.0, 127.3, 126.9, 126.4, 125.5, 124.9, 56.8, 39.5, -4.3, -5.0. GC-MS(EI-70eV): m/z (%) 342 (52), 135 (100), 264 (31), 249 (15), 75 (13). HRMS (ESI): calcd for $C_{23}H_{22}NaOSi^+$ $[M+Na]^+$: 365.1332, found: 365.1332.

3-(dimethyl(p-tolyl)silyl)-2-phenyl-2,3-dihydro-1H-inden-1-one

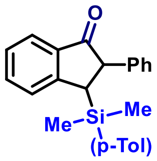

3ga

Purification by flash column chromatography (petroleum ether/EtOAc = 60:1). Yellow oily (28.8 mg, 81%). ^{1}H NMR (500 MHz, $CDCl_3$) δ 7.78 (d, *J* = 7.8 Hz, 1H), 7.56 (t, *J* = 7.7 Hz, 1H), 7.35 (t, *J* = 7.5 Hz, 1H), 7.34 - 7.29 (m, 3H), 7.24 (d, *J* = 4.0 Hz, 2H), 7.21 – 7.17 (m, 3H), 7.03 (d, *J* = 6.4 Hz, 2H), 3.76 (d, *J* = 2.4 Hz, 1H), 3.15 (d, *J* = 2.6 Hz, 1H), 2.41 (s, 3H), 0.33 (d, *J* = 5.6 Hz, 6H).^{13}C NMR (126 MHz, $CDCl_3$) δ 206.3, 157.8, 140.9, 139.7, 135.3, 134.8, 134.1, 132.3, 128.9, 128.8, 127.4, 126.9, 126.4, 125.6, 124.9, 56.8, 39.5, 21.5, -4.0, -4.9. GC-MS(EI-70eV): m/z (%) 356 (43), 149 (100), 282 (22), 364 (60), 249 (21). HRMS (ESI): calcd for $C_{24}H_{24}NaOSi^{+}$ $[M+Na]^{+}$: 379.1489, found: 379.1484.

3-((4-methoxyphenyl) dimethylsilyl)-2-phenyl-2,3-dihydro-1H-inden-1-one

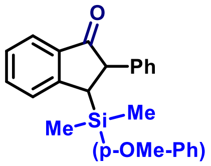

3ha

Purification by flash column chromatography (petroleum ether/EtOAc = 40:1). Yellow oily (29.0 mg, 78%). ^{1}H NMR (500 MHz, $CDCl_3$) δ 7.74 (d, *J* = 7.8 Hz, 1H), 7.52 (t, *J* = 7.5 Hz, 1H), 7.31 (t, *J* = 8.8 Hz, 3H), 7.24 – 7.16 (m, 3H), 7.13 (d, *J* = 8.7 Hz, 1H), 6.99 (d, *J* = 6.6 Hz, 2H), 6.88 (d, *J* = 8.7 Hz, 2H), 3.82 (s, 3H), 3.71 (d, *J* = 2.3 Hz, 1H), 3.09 (d, *J* = 2.7 Hz, 1H), 0.29 (d, *J* = 6.3 Hz, 6H). ^{13}C NMR (126 MHz, $CDCl_3$) δ 206.3, 161.0, 157.9, 140.9, 135.5, 135.3, 134.7, 128.8, 127.3, 126.9, 126.7, 126.4, 125.5, 124.9, 113.8, 56.8, 55.1, 39.7, -3.9, -4.8. GC-MS(EI-70eV): m/z (%) 372 (18), 165 (100), 298 (11), 264 (70), 249 (17). HRMS (ESI): calcd for $C_{24}H_{24}NaO_2Si^{+}$ $[M+Na]^{+}$: 395.1438, found: 395.1441.

3-((2-methoxyphenyl) dimethylsilyl)-2-phenyl-2,3-dihydro-1H-inden-1-one

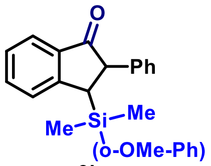

3ia

Purification by flash column chromatography (petroleum ether/EtOAc = 40:1). Yellow oily (27.2 mg, 73%). ^{1}H NMR (500 MHz, $CDCl_3$) δ 7.78 (d, *J* = 7.6 Hz, 1H), 7.53 (t, *J* = 7.6 Hz, 1H), 7.42 (t, *J* = 7.4 Hz, 1H), 7.33 (t, *J* = 8.4 Hz, 2H), 7.25 – 7.18 (m, 4H), 7.00 (t, *J* = 7.7 Hz, 1H), 6.93 (d, *J* = 6.4 Hz, 2H), 6.76 (d, *J* = 7.6 Hz, 1H), 3.69 (d, *J* = 2.6 Hz, 1H), 3.49 (s, 3H), 3.39 (d, *J* = 2.7 Hz, 1H), 0.38 (s, 3H), 0.25 (s, 3H). ^{13}C NMR (126 MHz, $CDCl_3$) δ 207.1, 164.1, 158.7, 141.3, 135.6, 135.3, 134.7, 131.7, 128.7, 127.5, 126.7, 126.1, 125.5, 124.7, 124.2, 120.6, 109.5, 57.0, 54.6, 38.8, -3.5, -4.5. GC-MS(EI-70eV): m/z (%) 372 (13), 135 (100), 264 (11), 165 (24), 91 (13). HRMS (ESI): calcd for $C_{24}H_{24}NaO_2Si^{+}$ $[M+Na]^{+}$: 395.1438, found: 395.1437.

3-((4-fluorophenyl) dimethylsilyl)-2-phenyl-2,3-dihydro-1H-inden-1-one

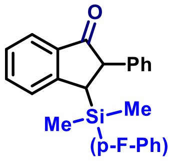

3ja

Purification by flash column chromatography (petroleum ether/EtOAc = 100:1). Yellow oily (26.6 mg, 74%). ^{1}H NMR (400 MHz, $CDCl_3$) δ 7.74 (d, *J* = 7.4 Hz, 1H), 7.53 (t, *J* = 7.5 Hz, 1H), 7.36 – 7.30 (m, 3H), 7.21 (d, *J* = 7.6 Hz, 3H), 7.12 (d, *J* = 7.8 Hz, 1H), 7.03 (t, *J* = 8.9 Hz, 2H), 6.96 (d, *J* = 8.3 Hz, 2H), 3.67 (d, *J* = 2.5 Hz, 1H), 3.09 (d, *J* = 2.8 Hz, 1H), 0.31 (s, 6H). ^{13}C NMR (101 MHz, $CDCl_3$) δ 206.0, 157.4, 140.6, 136.0 (d, *J* = 137.1 Hz), 135.3, 134.8, 128.9, 127.2 (d, *J* = 25.3 Hz)1, 126.5, 125.2 (d, *J* = 37.4 Hz), 115.3 (d, *J* =20.2 Hz), 56.7, 39.4, -4.1, -4.8. ^{19}F NMR (376 MHz, $CDCl_3$) δ -110.59. GC-MS(EI-70eV): m/z (%) 360 (50), 153 (100), 286 (24), 264 (34), 91 (23). HRMS (ESI): calcd for $C_{23}H_{21}FNaOSi^{+}$ $[M+Na]^{+}$: 383.1238, found: 383.1235.

3 -((4-chlorophenyl) dimethylsilyl)-2-phenyl-2,3-dihydro-1H-inden-1-one

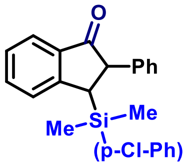

3ka

Purification by flash column chromatography (petroleum ether/EtOAc = 100:1). Yellow oily (28.6 mg, 76%). ^{1}H NMR (600 MHz, $CDCl_3$) δ 7.75 (d, *J* = 7.7 Hz, 1H), 7.53 (t, *J* = 7.5 Hz ,1H), 7.33 (t, *J* = 7.4 Hz, 2H), 7.30 (d, *J* = 4.0 Hz, 3H), 7.21 (t, *J* = 5.5 Hz, 3H), 7.13 (dt, *J* = 7.8, 1.0 Hz, 1H), 6.98 (d, *J* = 6.6 Hz, 2H), 3.67 (d, *J* = 2.4 Hz, 1H), 3.11 (d, *J* = 2.6 Hz, 1H), 0.31 (d, *J* = 3.5 Hz, 6H). ^{13}C NMR (151 MHz, $CDCl_3$) δ 205.9, 157.3, 140.6, 136.2,

135.4, 134.9, 134.3, 128.9, 128.3, 127.9, 127.3, 127.0, 126.6, 125.4, 125.1, 56.7, 39.2, -4.2, -4.9. GC-MS(EI-70eV): m/z (%) 376 (59), 169 (100), 264 (53), 91 (31), 75 (29). HRMS (ESI): calcd for $C_{23}H_{21}ClNaOSi^{+}$ $[M+Na]^{+}$: 399.0942, found: 399.0939.

2-(phenyl-d5)-3-(triethylsilyl)-2,3-dihydro-1H-inden-1-one-4,5,6,7-d4 and 2-phenyl-3-(triethylsilyl)-2,3-dihydro-1H-inden-1-one

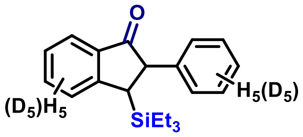

3aa+[D]-3aa, 33%
KIE=3.0

Purification by flash column chromatography (petroleum ether/EtOAc = 120:1). Yellow oily. ^{1}H NMR (500 MHz, $CDCl_3$) δ 7.79 (d, J = 7.8 Hz, 1H), 7.62 (t, J = 7.6 Hz, 1H), 7.44 (d, J = 7.9 Hz, 1H), 7.34 (t, J = 7.4 Hz, 1H), 7.30 – 7.27 (m, 1H), 7.22 (t, J = 7.3 Hz, 1H), 7.13 (d, J = 6.9 Hz, 2H), 3.79 (d, J = 2.3 Hz, 1H), 3.05 (d, J = 2.1 Hz, 1H), 0.88 (t, J = 7.9 Hz, 9H), 0.64 (q, J = 7.9 Hz, 6H).

2-(phenyl-d5)-3-(triethylsilyl)-2,3-dihydro-1H-inden-1-one-4,5,6,7-d4

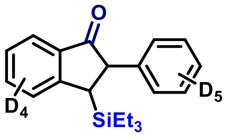

[D]-3aa, 33%

Purification by flash column chromatography (petroleum ether/EtOAc = 120:1). Yellow oily. ^{1}H NMR (500 MHz, $CDCl_3$) δ 3.77 (d, J = 2.3 Hz, 1H), 3.03 (d, J = 2.3 Hz, 1H), 0.86 (t, J = 7.9 Hz, 9H), 0.62 (q, J = 7.9 Hz, 6H).

4. NMR Spectrum Copies

^{1}H NMR (600 MHz) Spectrum of **3aa** in $CDCl_3$

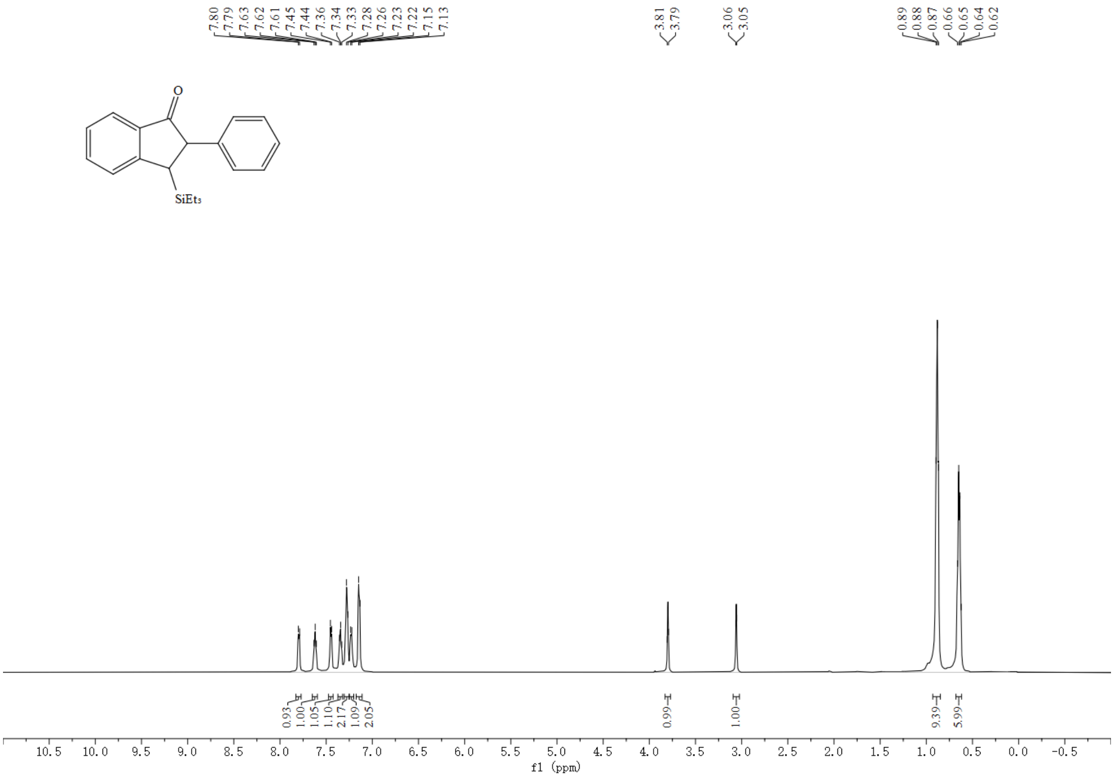

^{13}C{^{1}H} NMR (151 MHz) Spectrum of **3aa** in $CDCl_3$

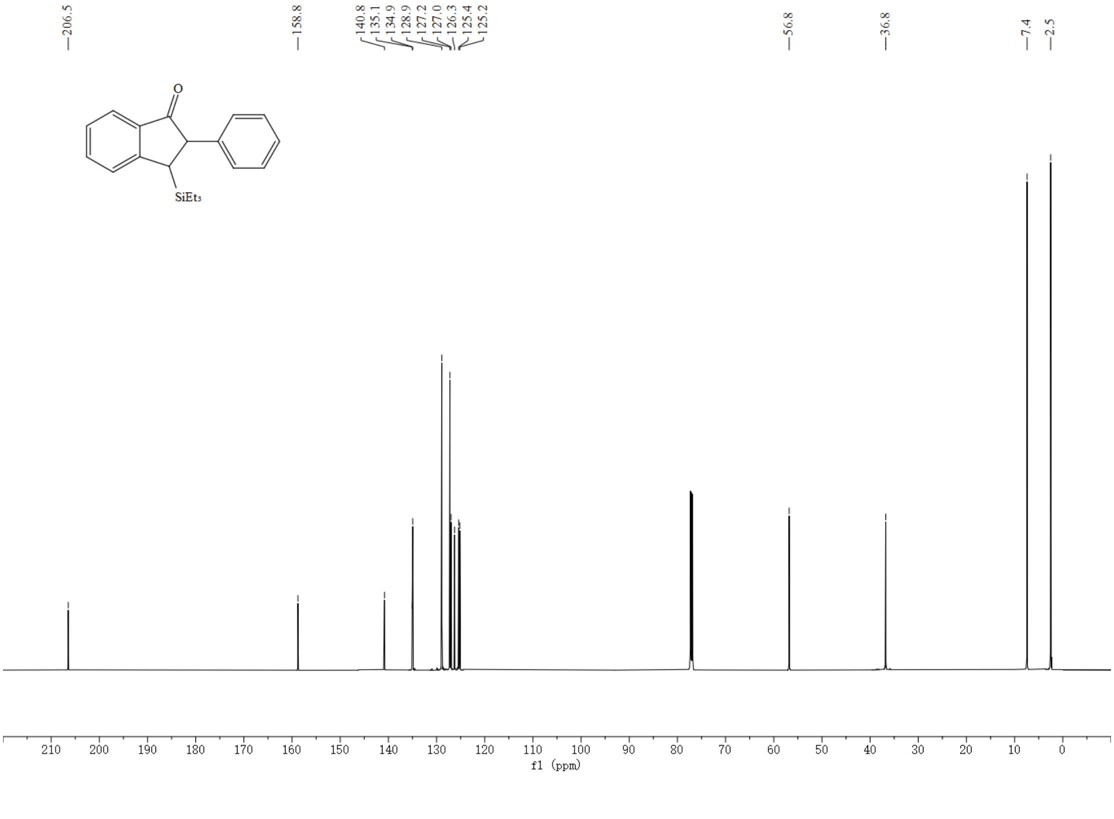

^{1}H NMR (500 MHz) Spectrum of **3ab** in $CDCl_3$

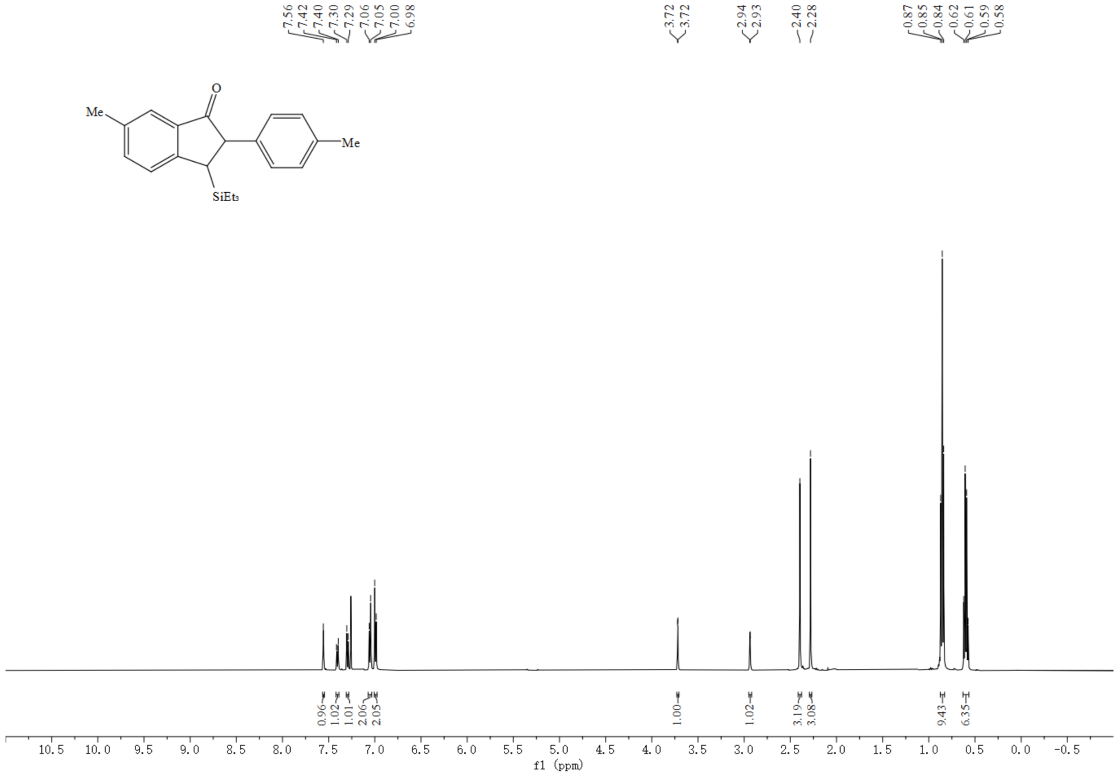

$^{13}C\{^{1}H\}$ NMR (126 MHz) Spectrum of **3ab** in $CDCl_3$

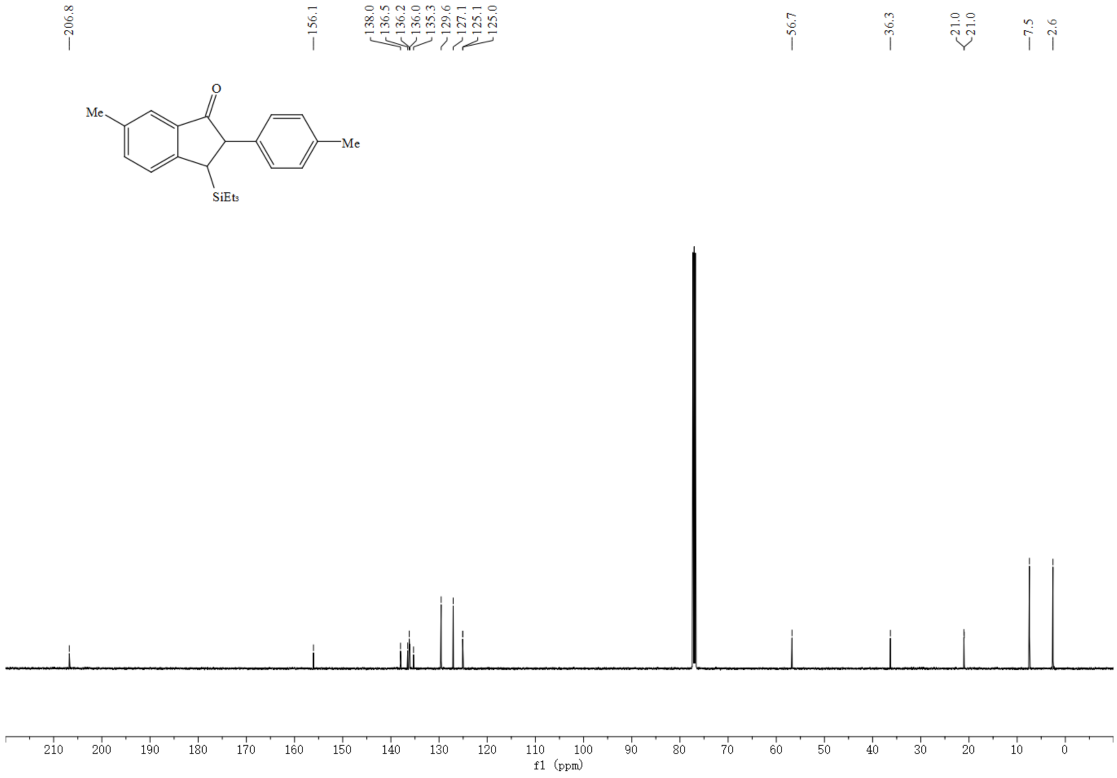

^{1}H NMR (500 MHz) Spectrum of **3ac** in $CDCl_3$

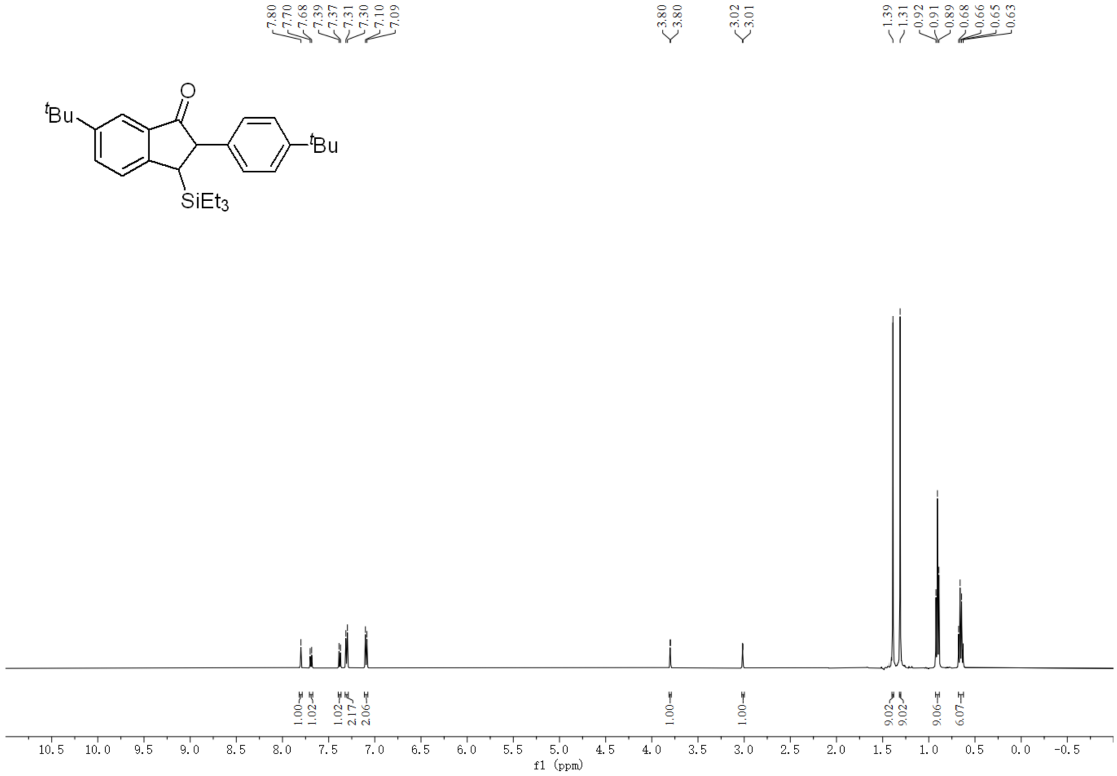

^{13}C{^{1}H} NMR (126 MHz) Spectrum of **3ac** in $CDCl_3$

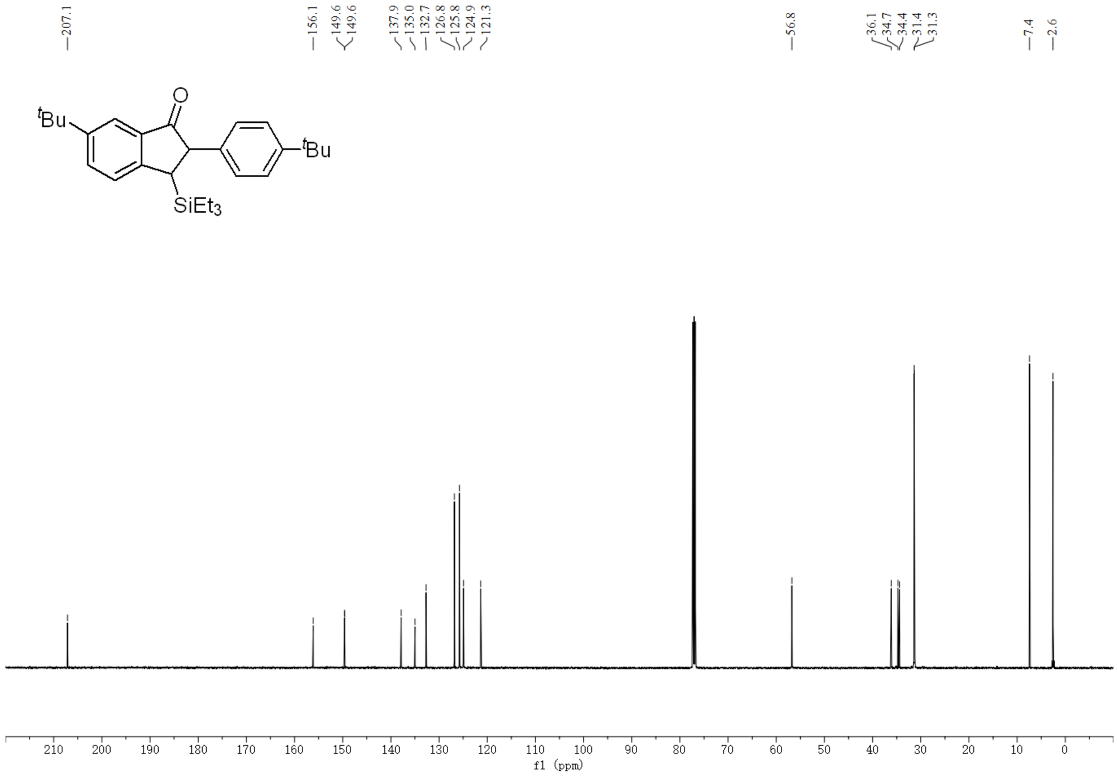

^{1}H NMR (500 MHz) Spectrum of **3ad** in $CDCl_3$

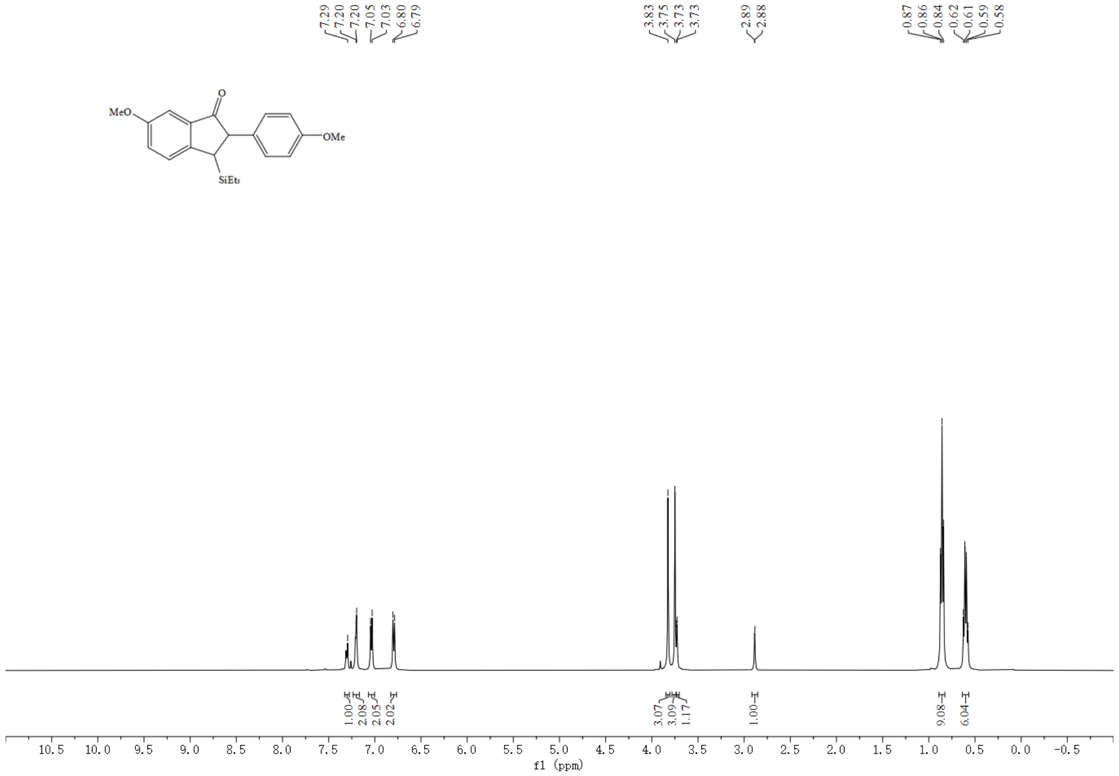

^{13}C{^{1}H} NMR (126 MHz) Spectrum of **3ad** in $CDCl_3$

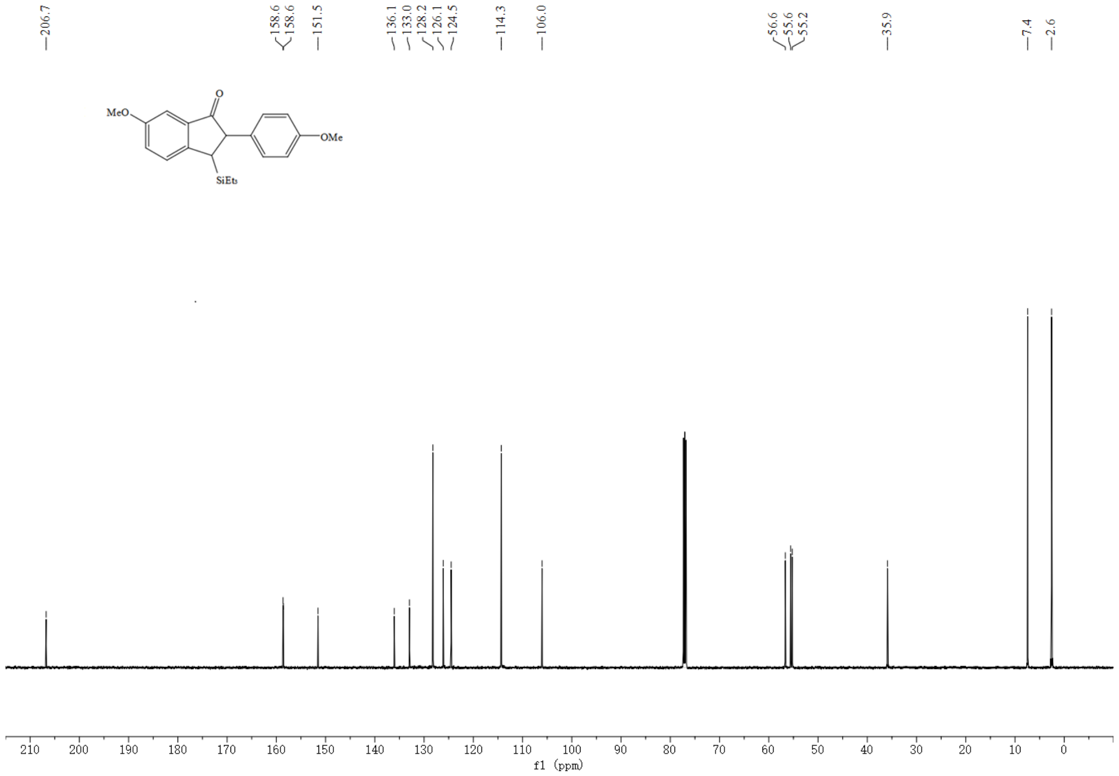

^{1}H NMR (500 MHz) Spectrum of **3ae** in $CDCl_3$

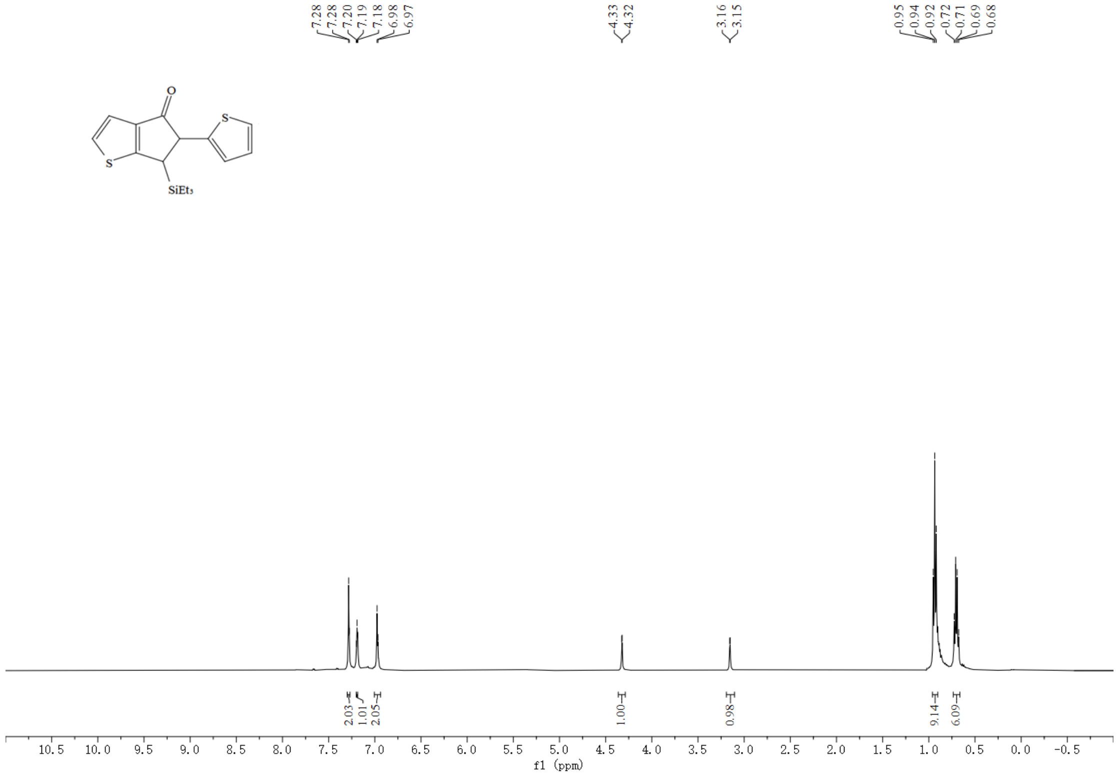

$^{13}C\{^{1}H\}$ NMR (126 MHz) Spectrum of **3ae** in $CDCl_3$

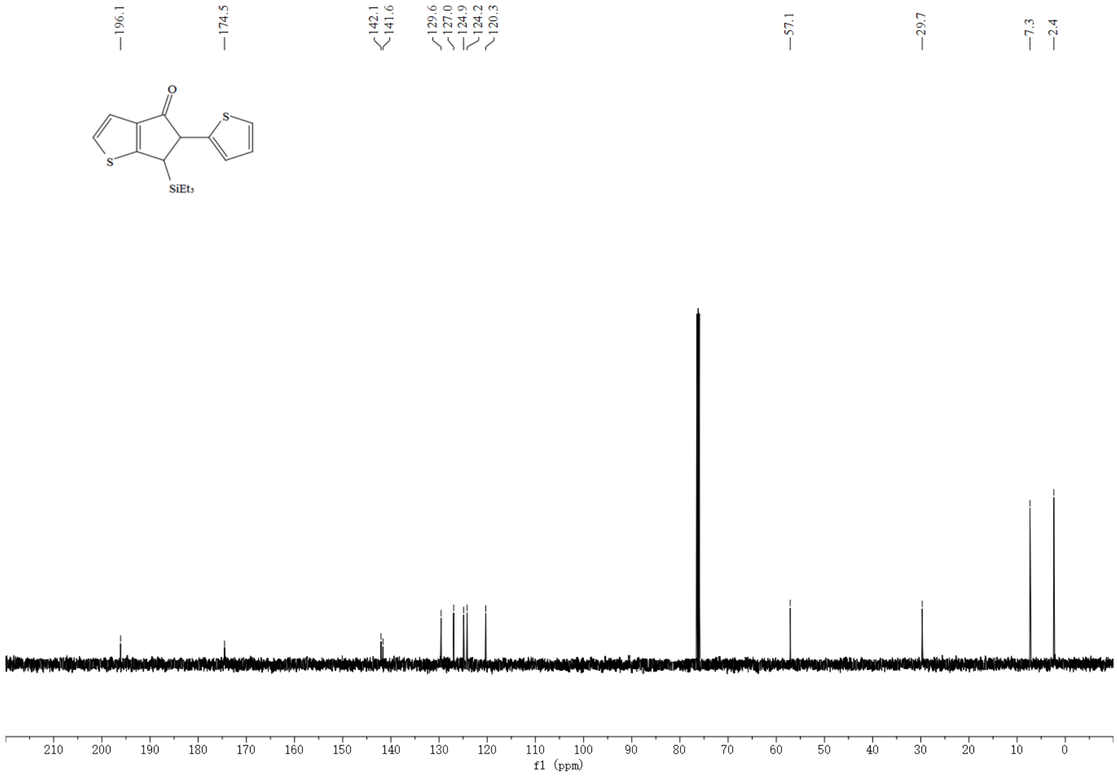

^{1}H NMR (500 MHz) Spectrum of **3af** in $CDCl_3$

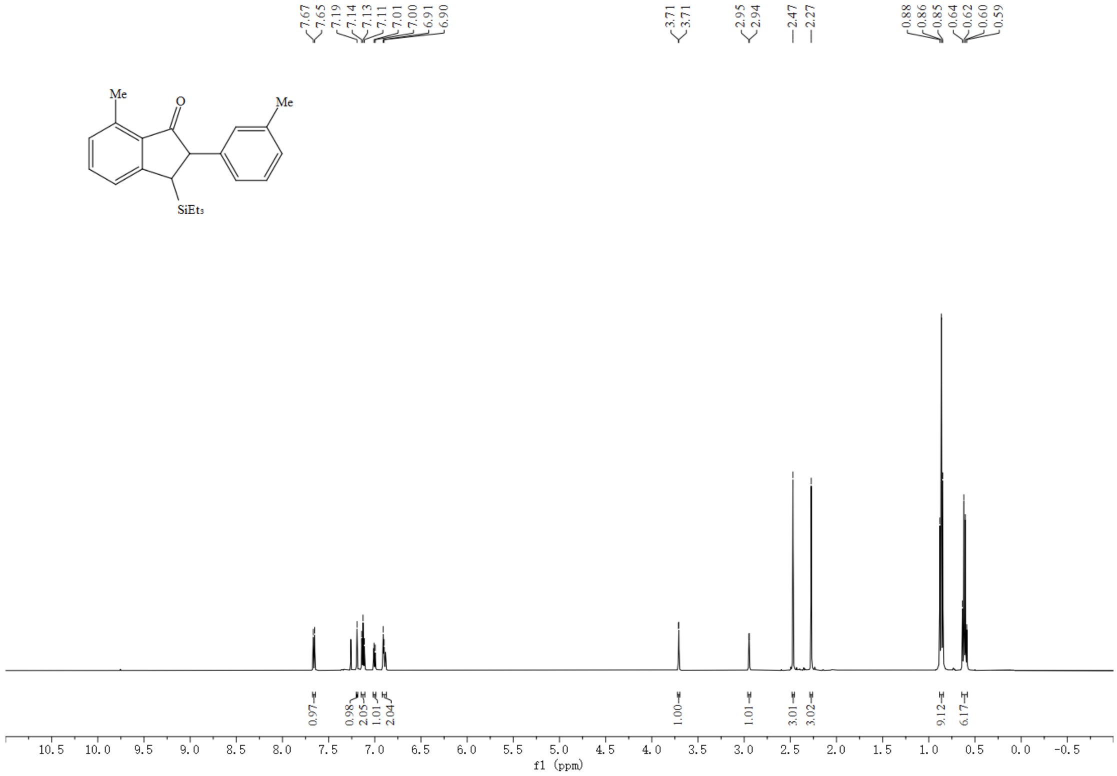

^{13}C{^{1}H} NMR (126 MHz) Spectrum of **3af** in $CDCl_3$

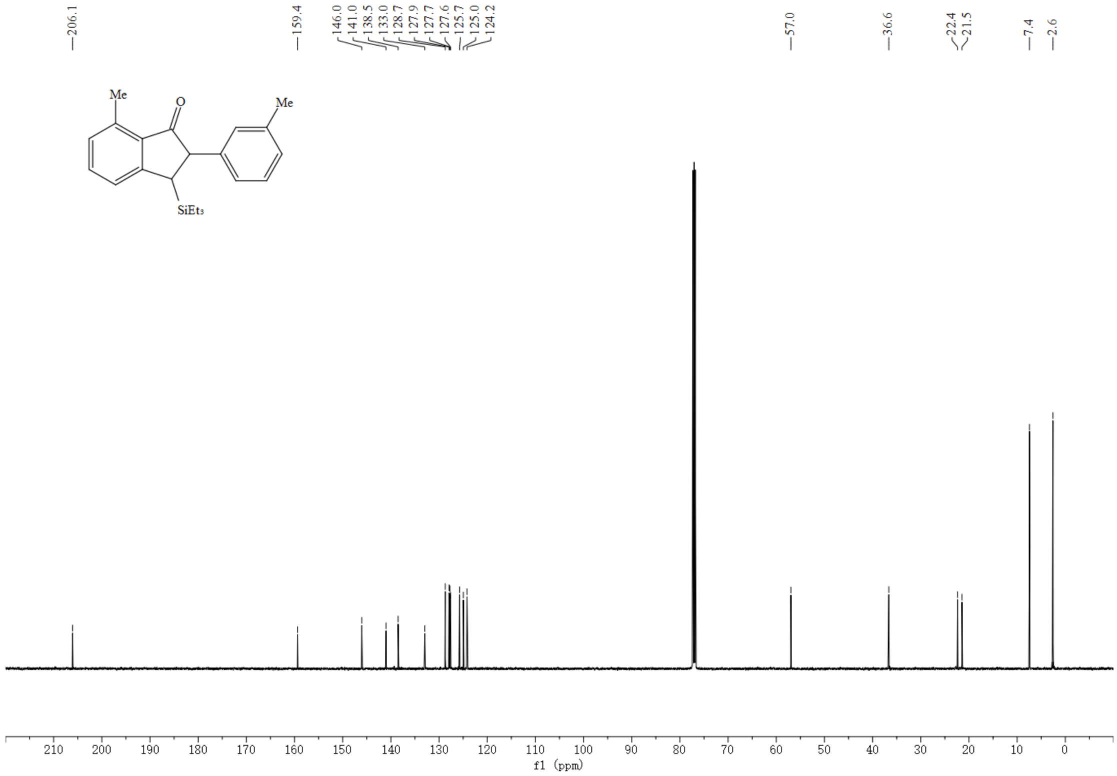

^{1}H NMR (500 MHz) Spectrum of **3ag** in $CDCl_3$

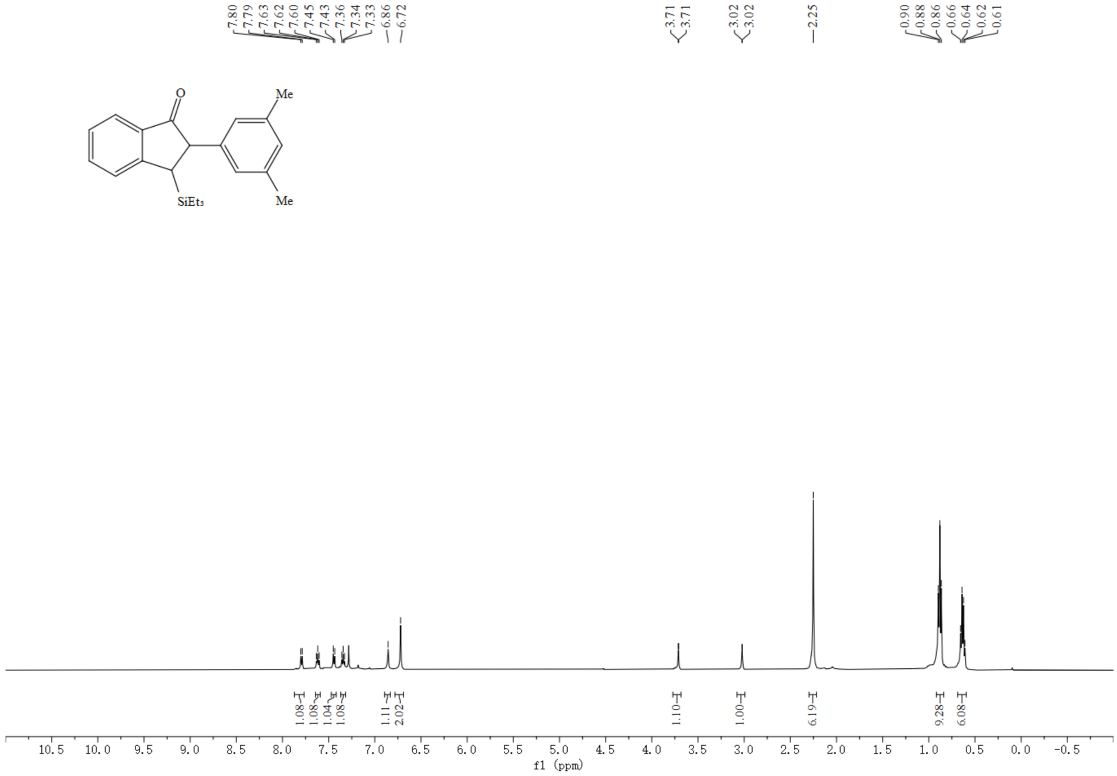

^{13}C{^{1}H} NMR (126 MHz) Spectrum of **3ag** in $CDCl_3$

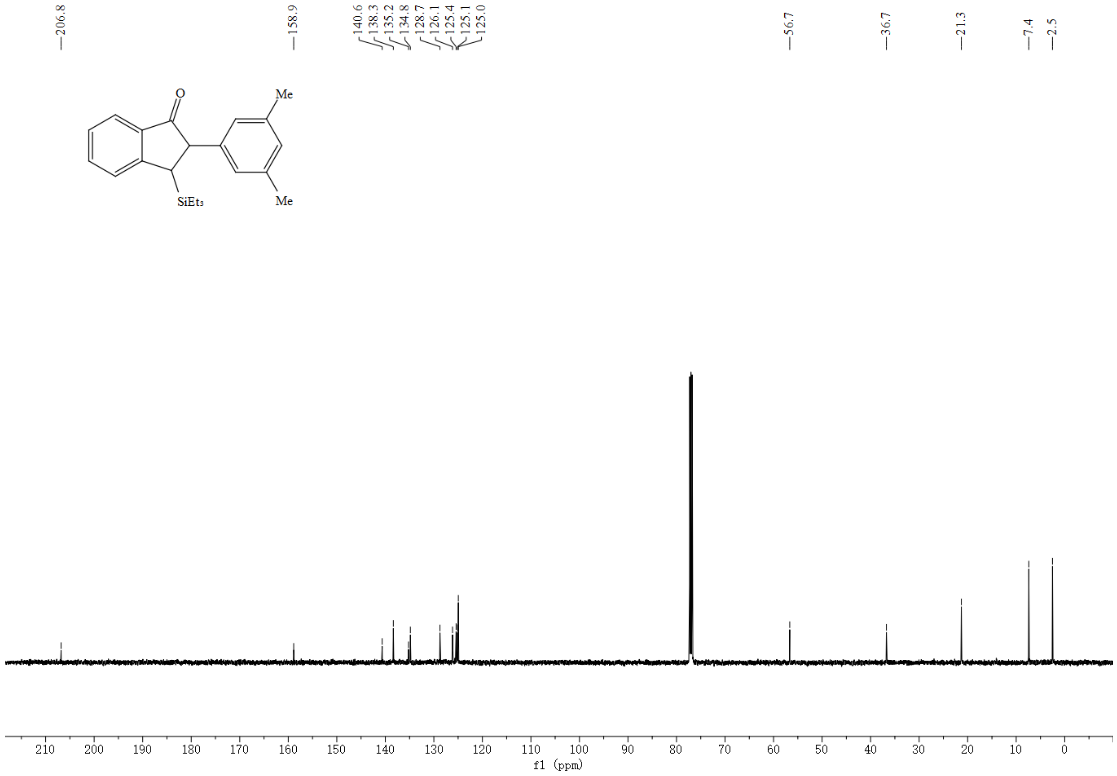

^{1}H NMR (500 MHz) Spectrum of **3ah** in $CDCl_3$

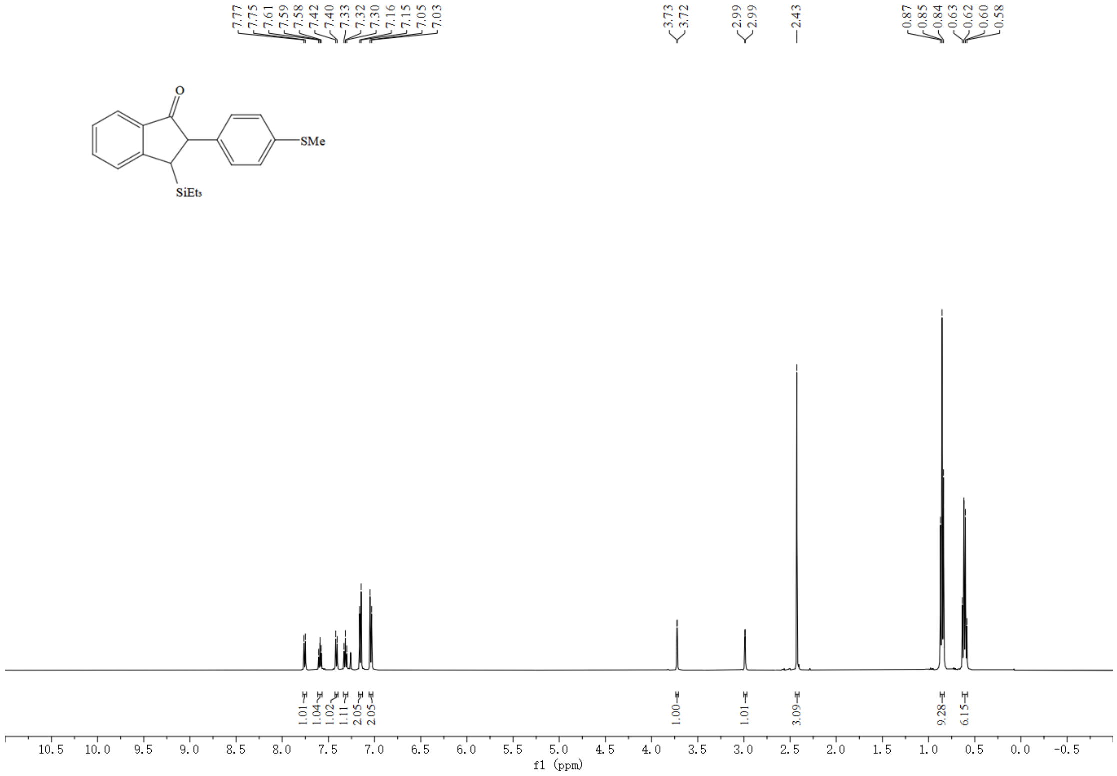

$^{13}C\{^{1}H\}$ NMR (126 MHz) Spectrum of **3ah** in $CDCl_3$

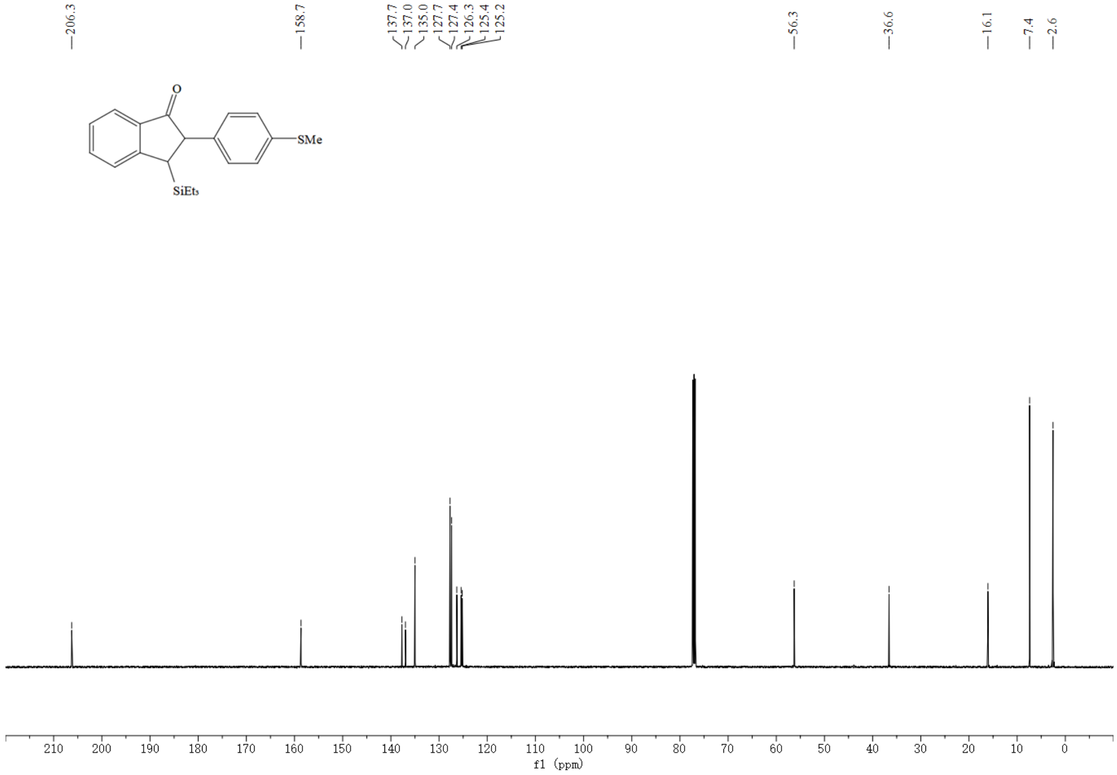

^{1}H NMR (500 MHz) Spectrum of **3ai** in $CDCl_3$

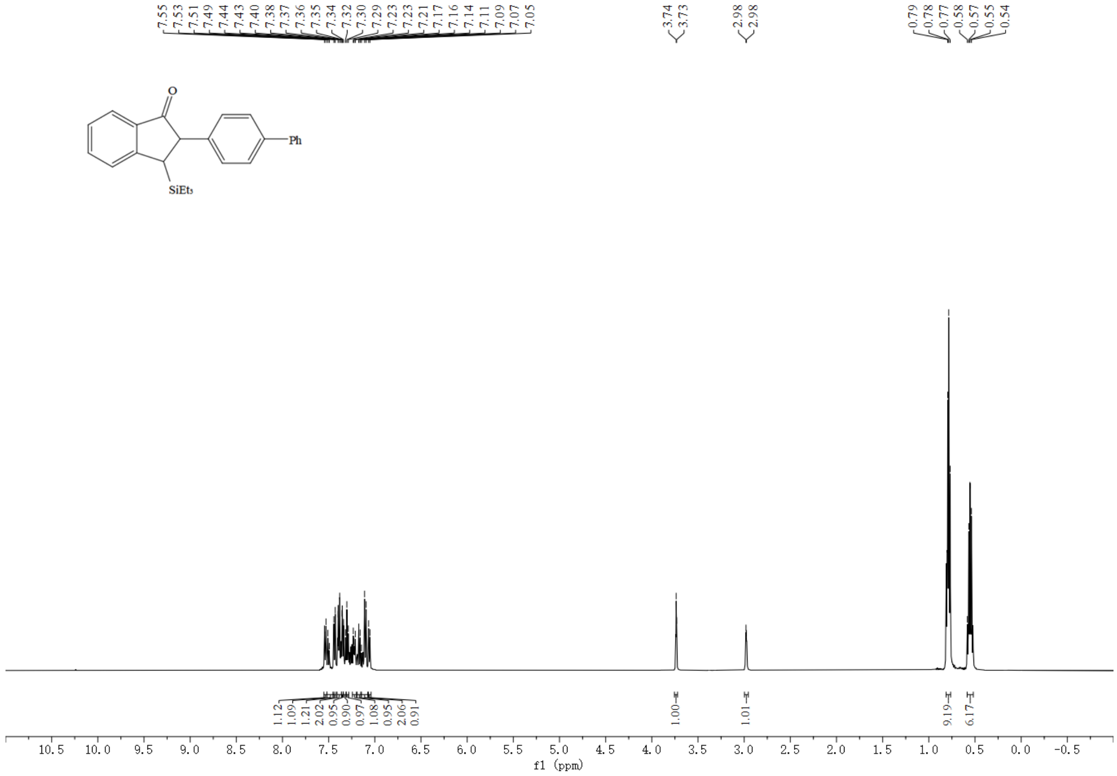

^{13}C{^{1}H} NMR (126 MHz) Spectrum of **3ai** in $CDCl_3$

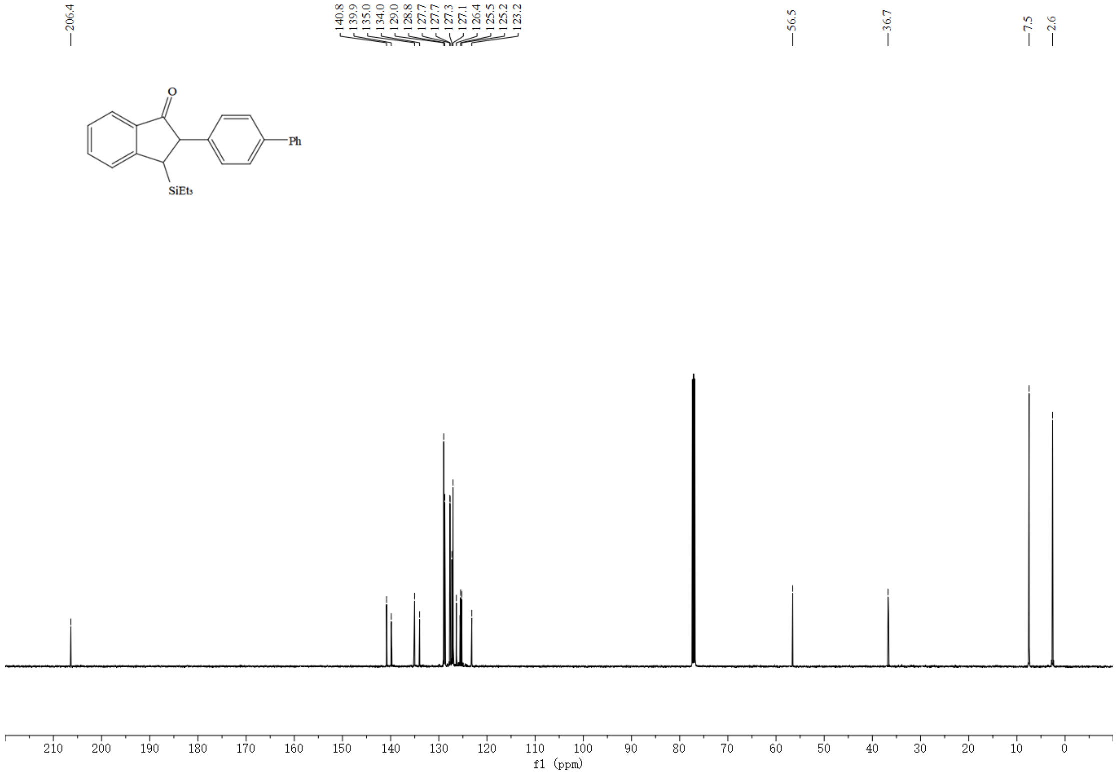

^{1}H NMR (500 MHz) Spectrum of **3aj** in $CDCl_3$

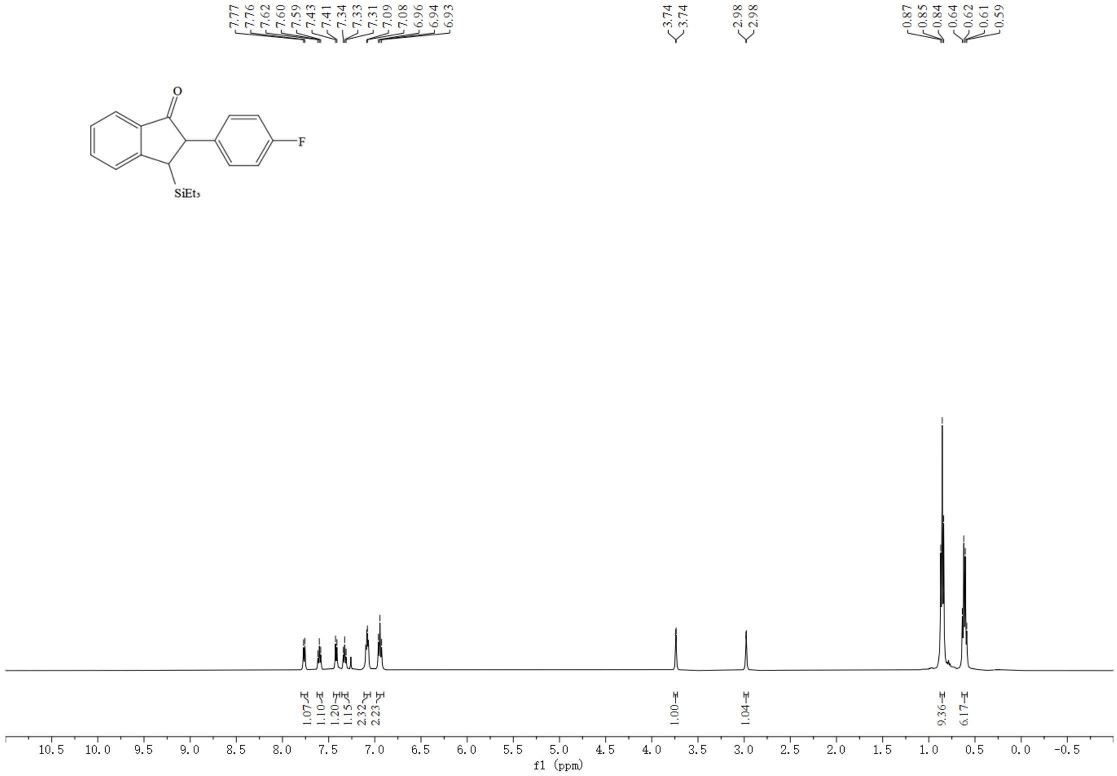

^{13}C{^{1}H} NMR (126 MHz) Spectrum of **3aj** in $CDCl_3$

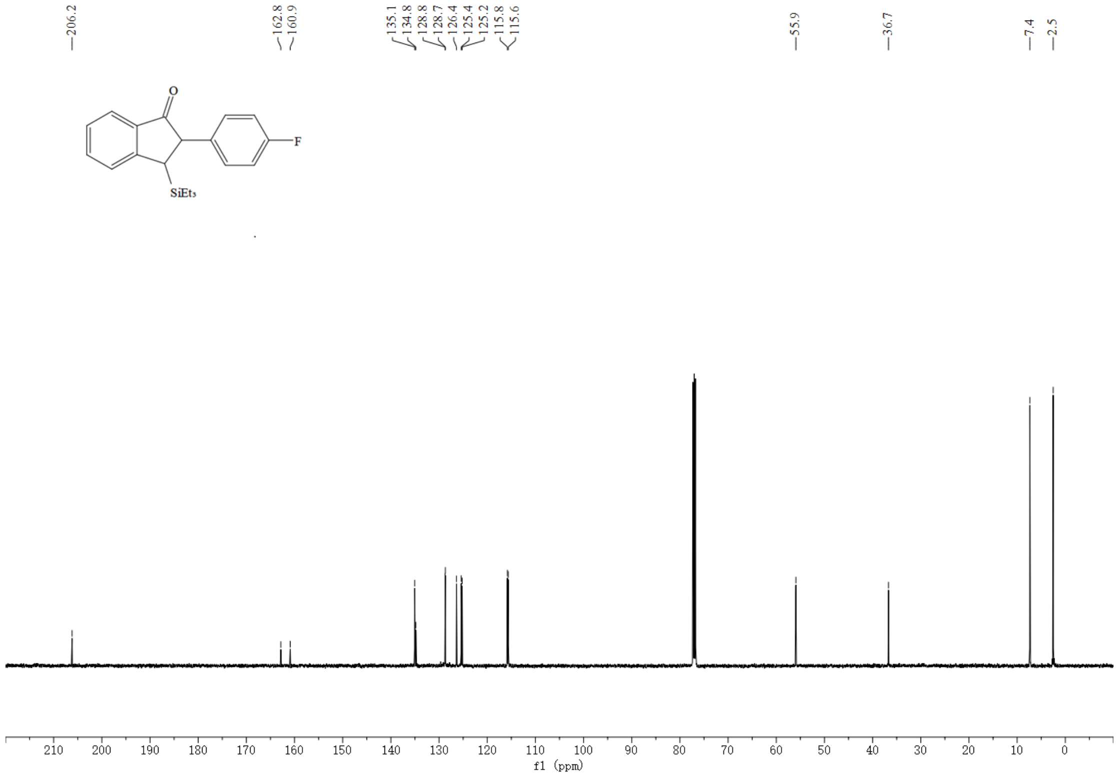

^{19}F NMR (471 MHz) Spectrum of **3aj** in $CDCl_3$

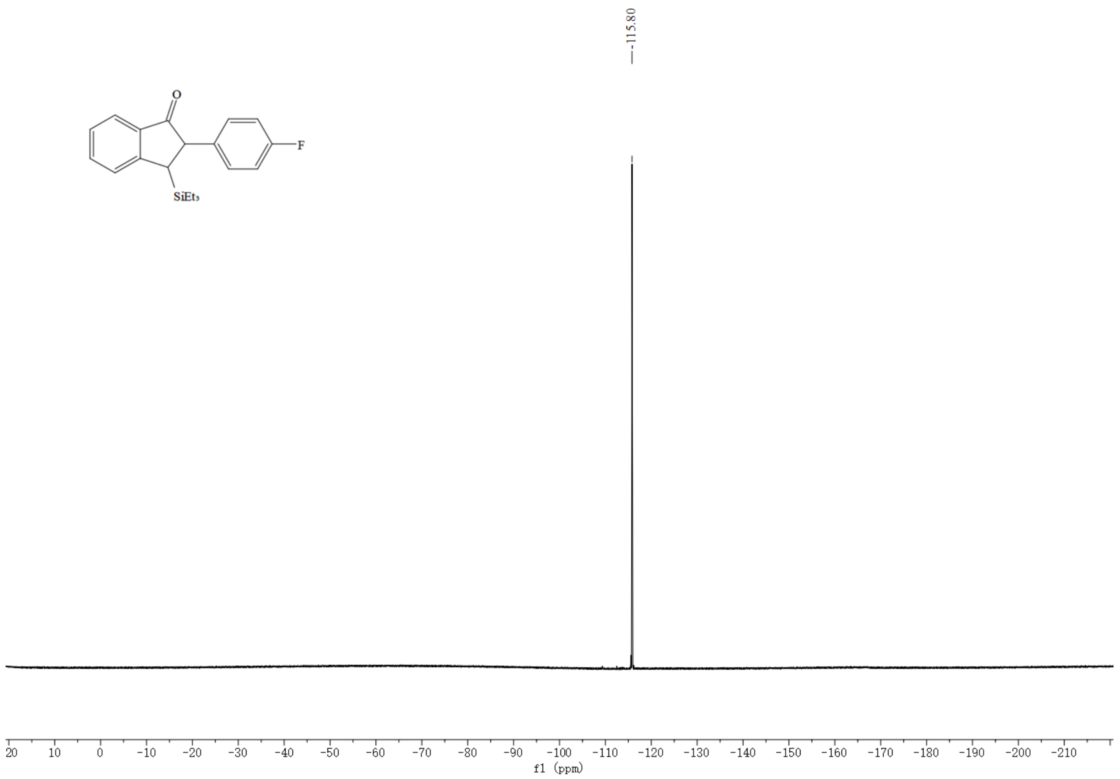

^{1}H NMR (500 MHz) Spectrum of **3ak** in $CDCl_3$

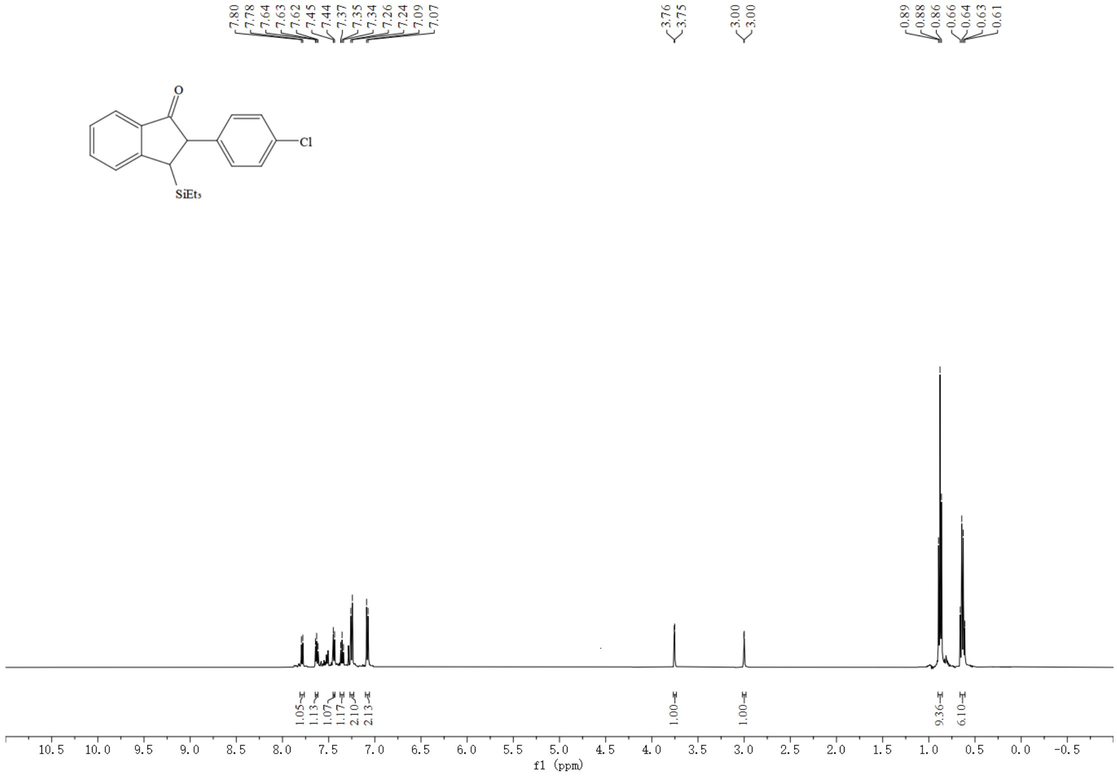

^{13}C{^{1}H} NMR (126 MHz) Spectrum of **3ak** in $CDCl_3$

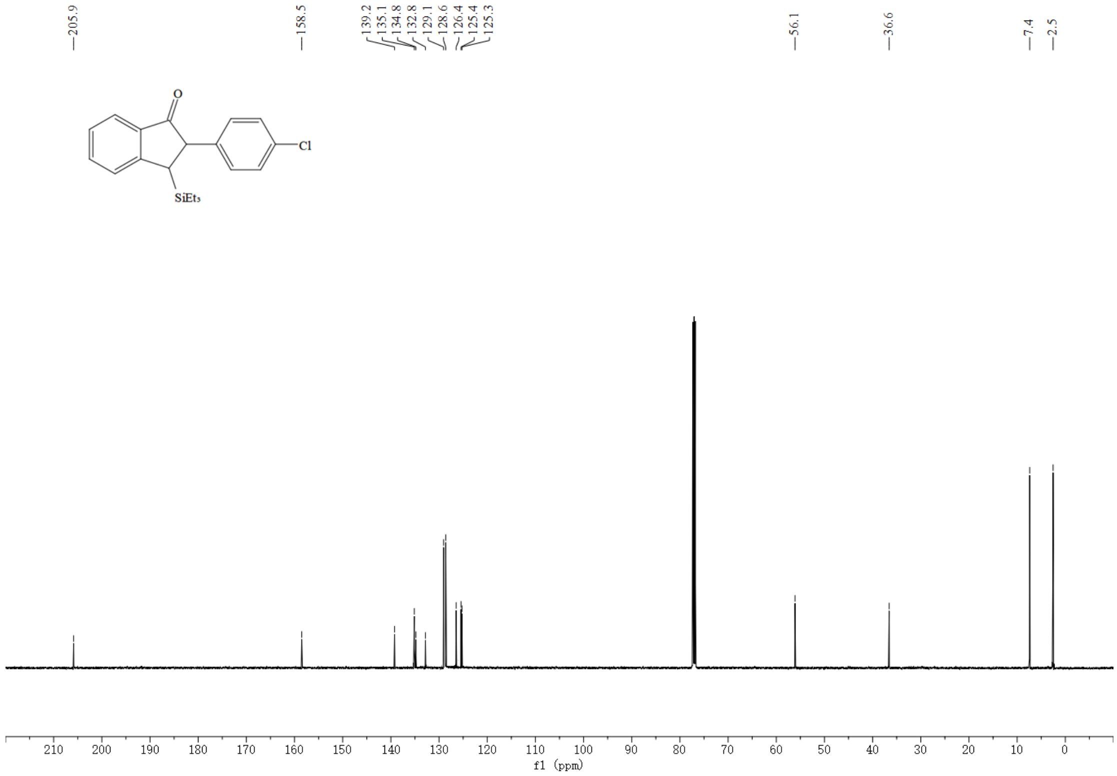

^{1}H NMR (500 MHz) Spectrum of **3al** in $CDCl_3$

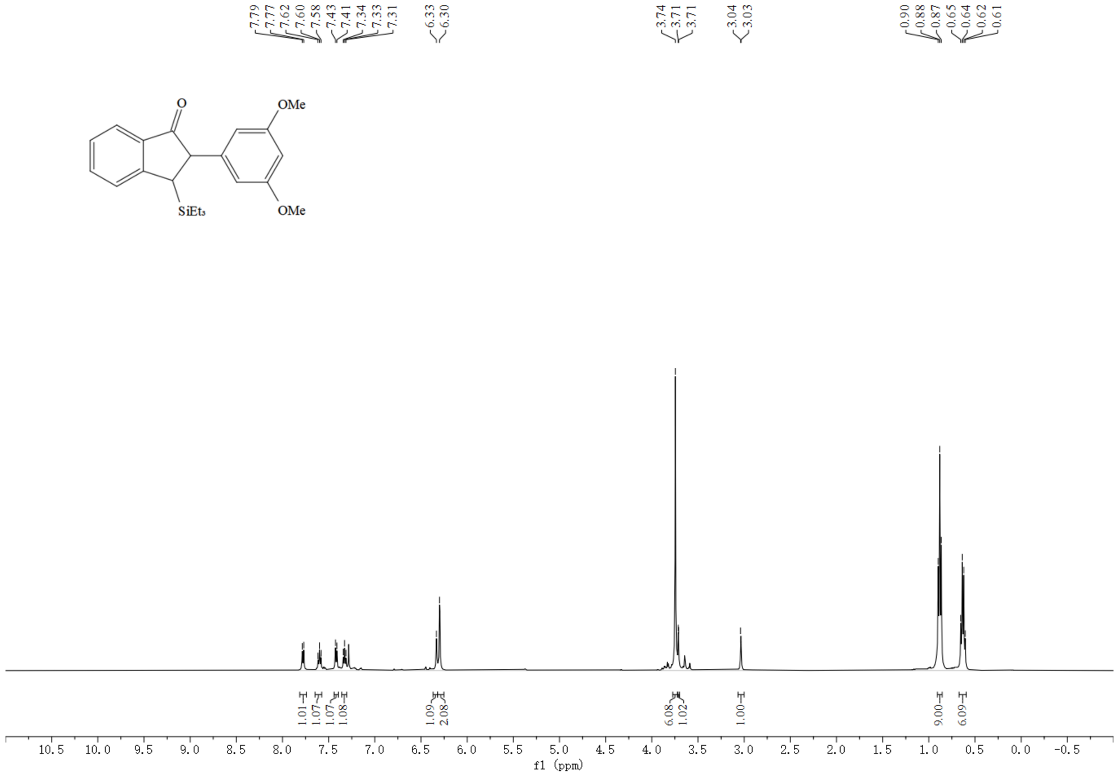

^{13}C{^{1}H} NMR (126 MHz) Spectrum of **3al** in $CDCl_3$

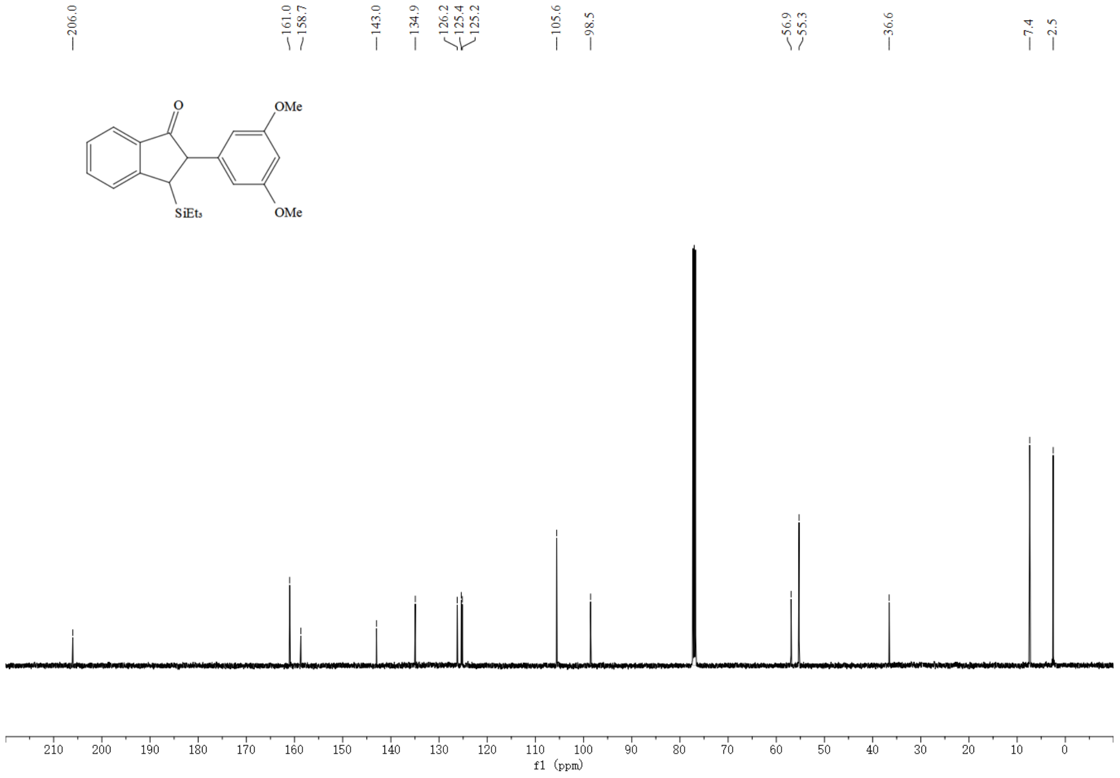

^{11}H NMR (500 MHz) Spectrum of **3am** in $CDCl_3$

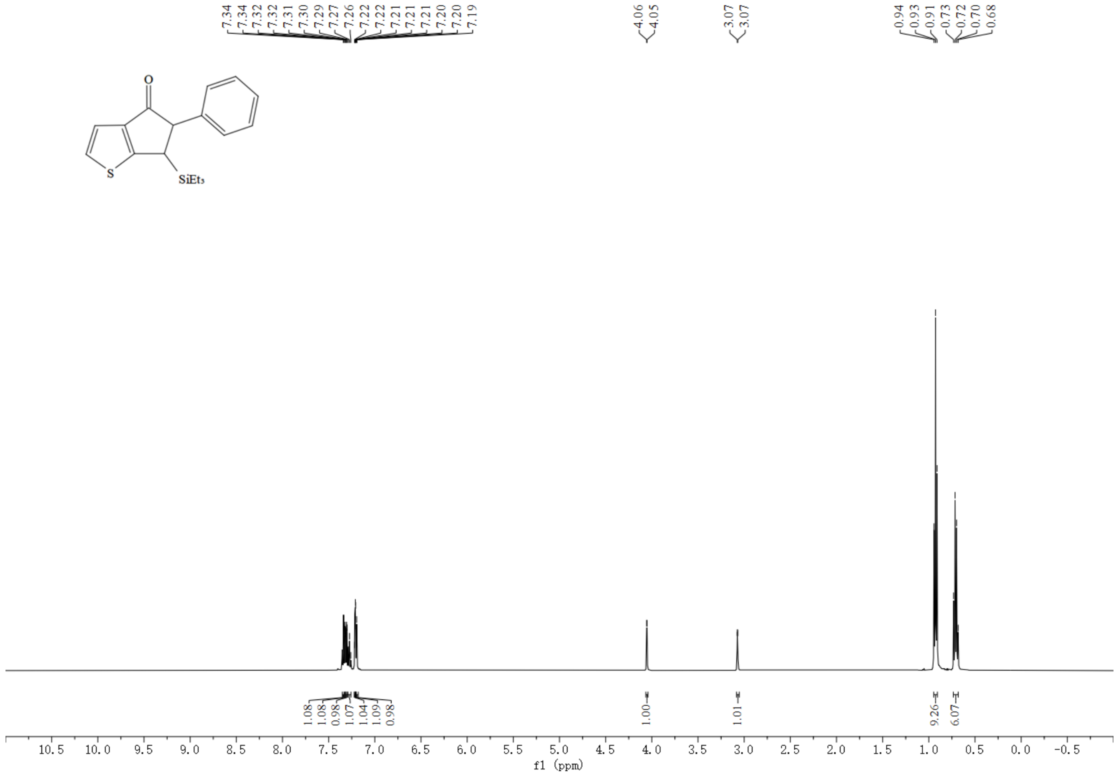

$^{13}C\{^1H\}$ NMR (126 MHz) Spectrum of **3am** in $CDCl_3$

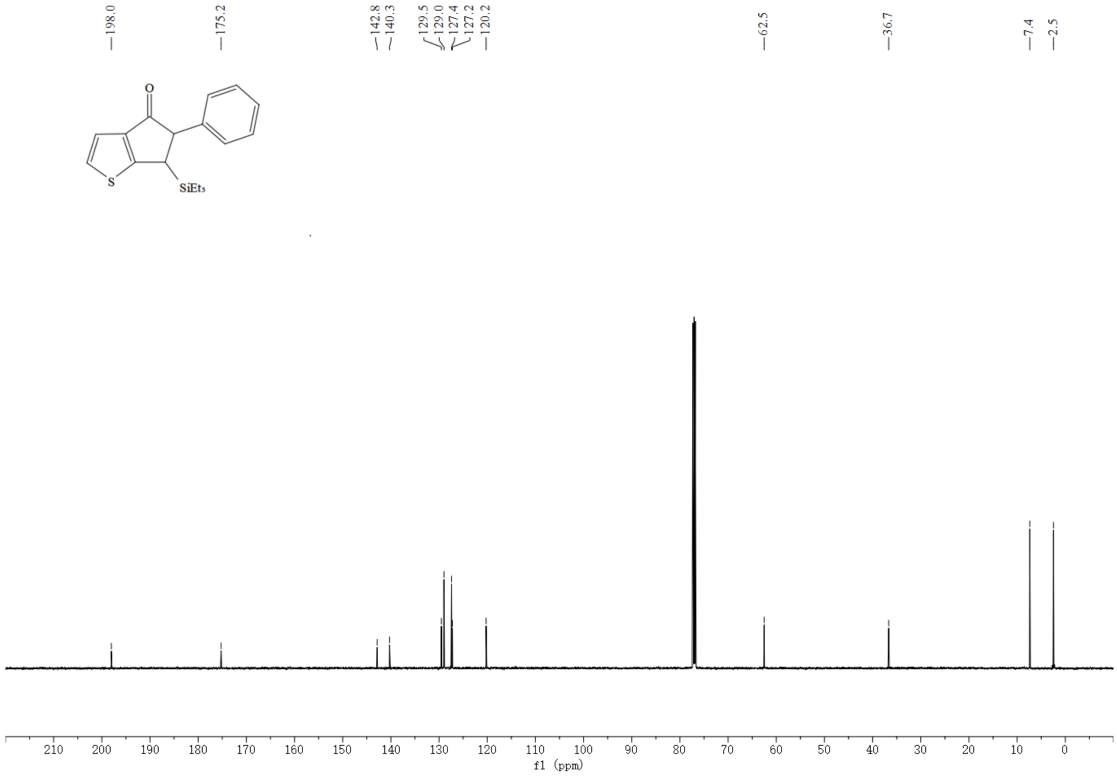

^{1}H NMR (500 MHz) Spectrum of **3an** in $CDCl_3$

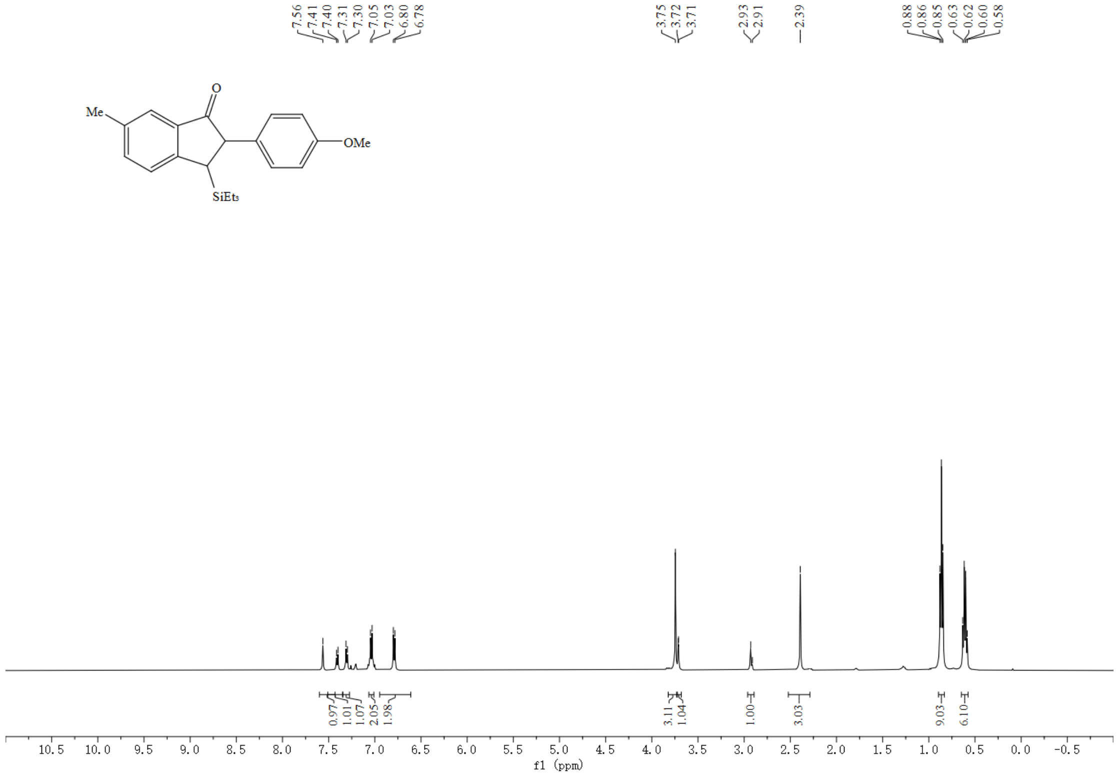

$^{13}C\{^{1}H\}$ NMR (126 MHz) Spectrum of **3an** in $CDCl_3$

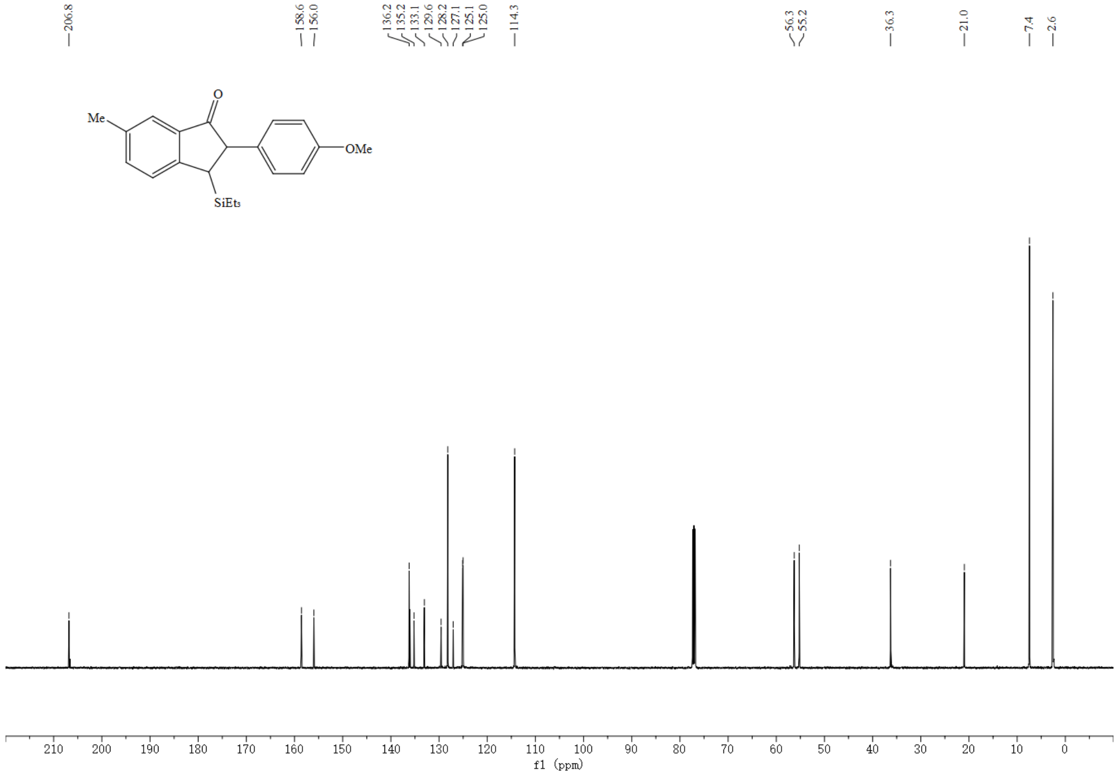

^{1}H NMR (500 MHz) Spectrum of **3ao** in $CDCl_3$

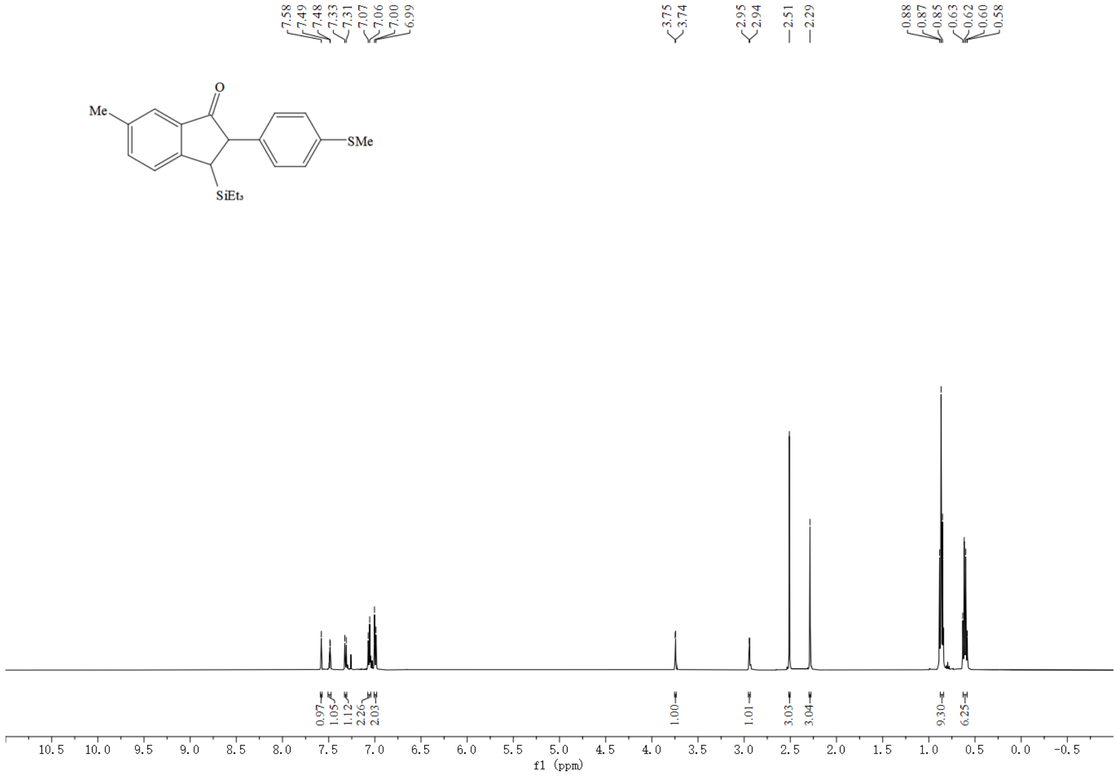

^{13}C{^{1}H} NMR (126 MHz) Spectrum of **3ao** in $CDCl_3$

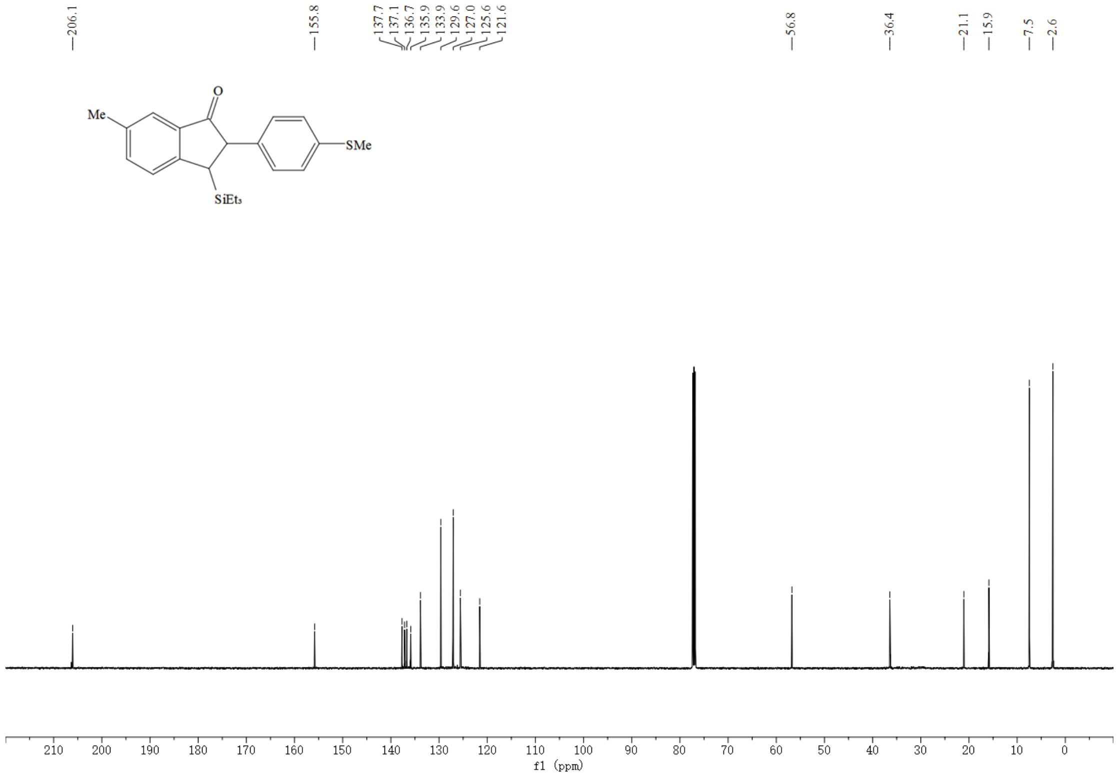

^{1}H NMR (500 MHz) Spectrum of **3ap** in $CDCl_3$

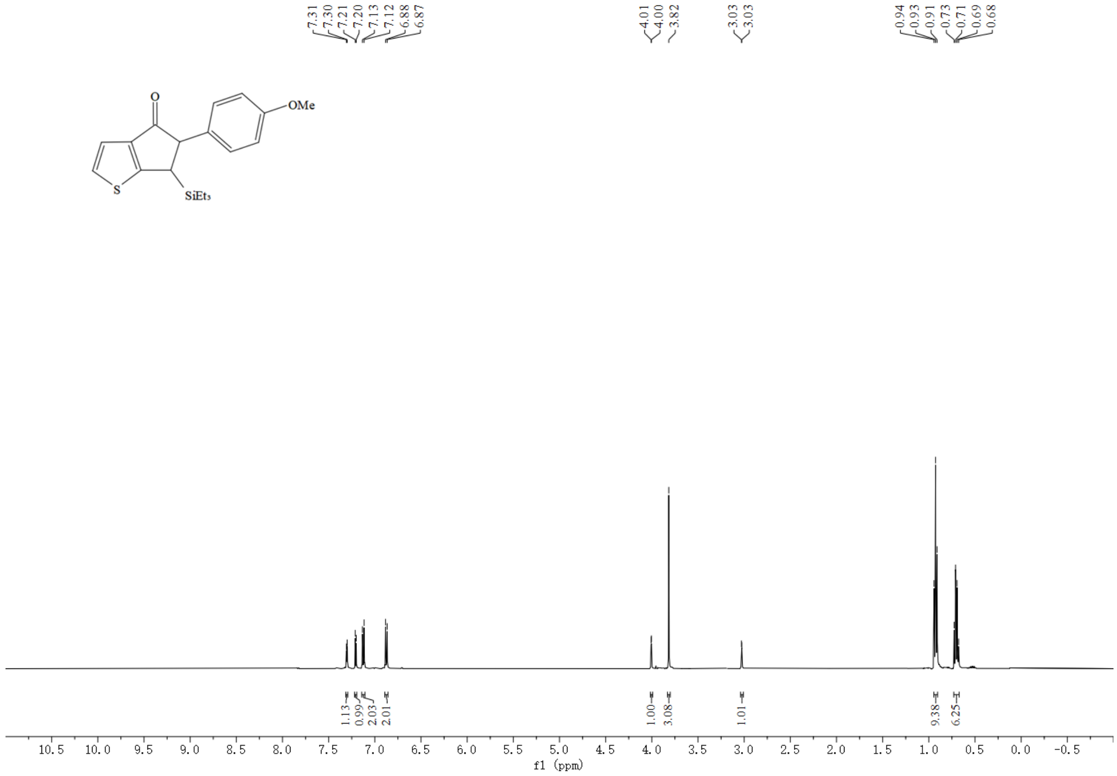

^{13}C{^{1}H} NMR (126 MHz) Spectrum of **3ap** in $CDCl_3$

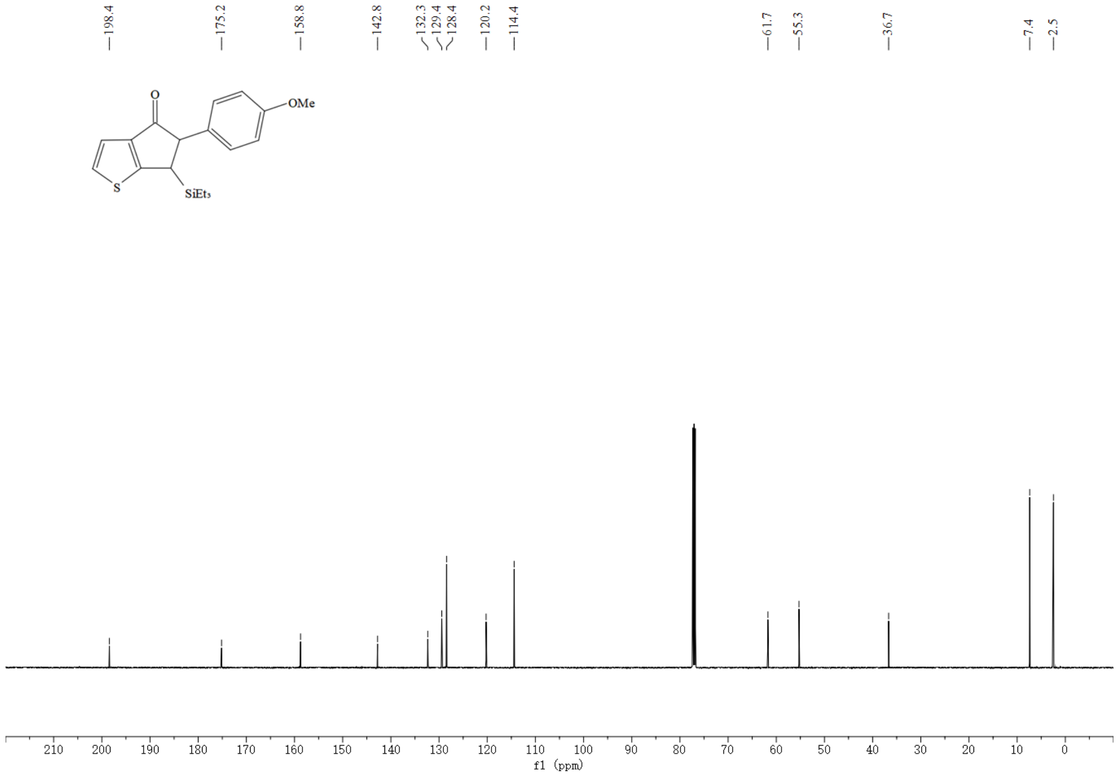

H NMR (500 MHz) Spectrum of **3aq** in $CDCl_3$

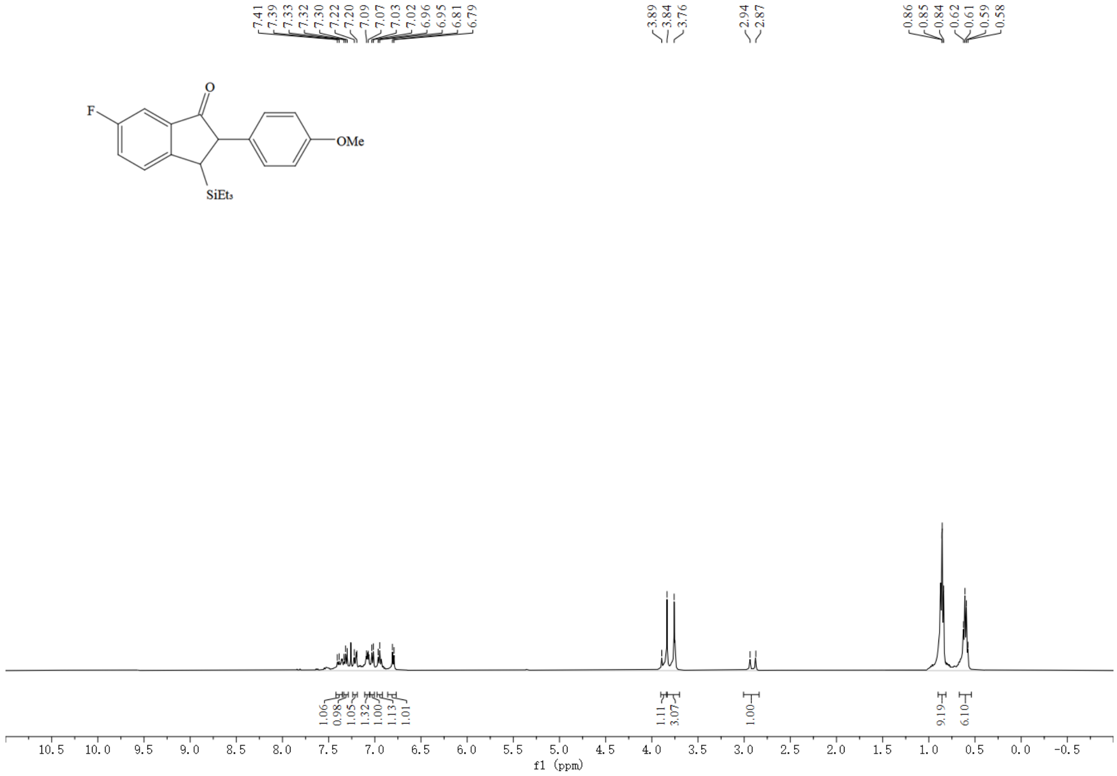

$^{13}C\{^{1}H\}$ NMR (126 MHz) Spectrum of **3aq** in $CDCl_3$

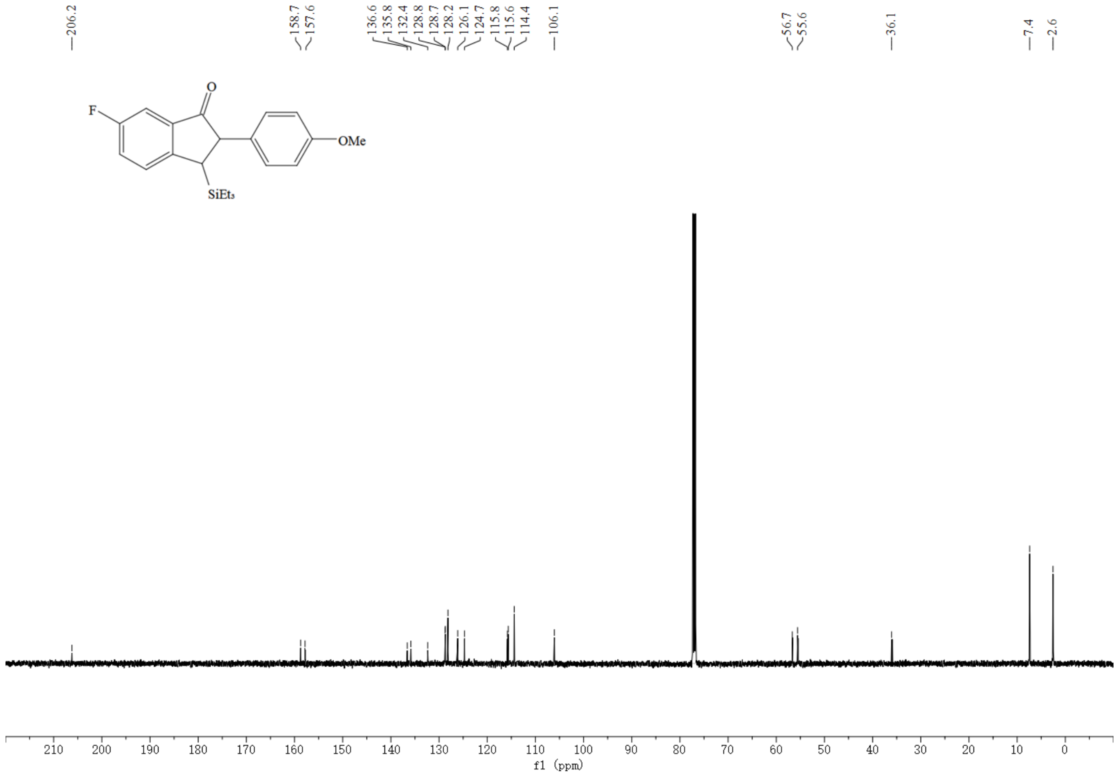

^{19}F NMR (471 MHz) Spectrum of **3aq** in $CDCl_3$

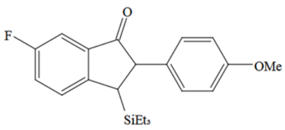

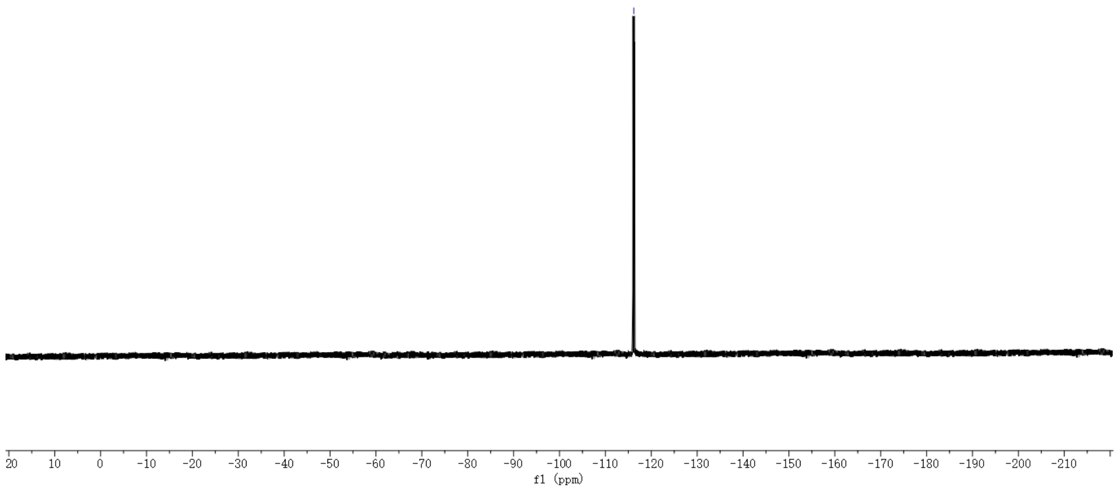

^{1}H NMR (500 MHz) Spectrum of **3ar** in $CDCl_3$

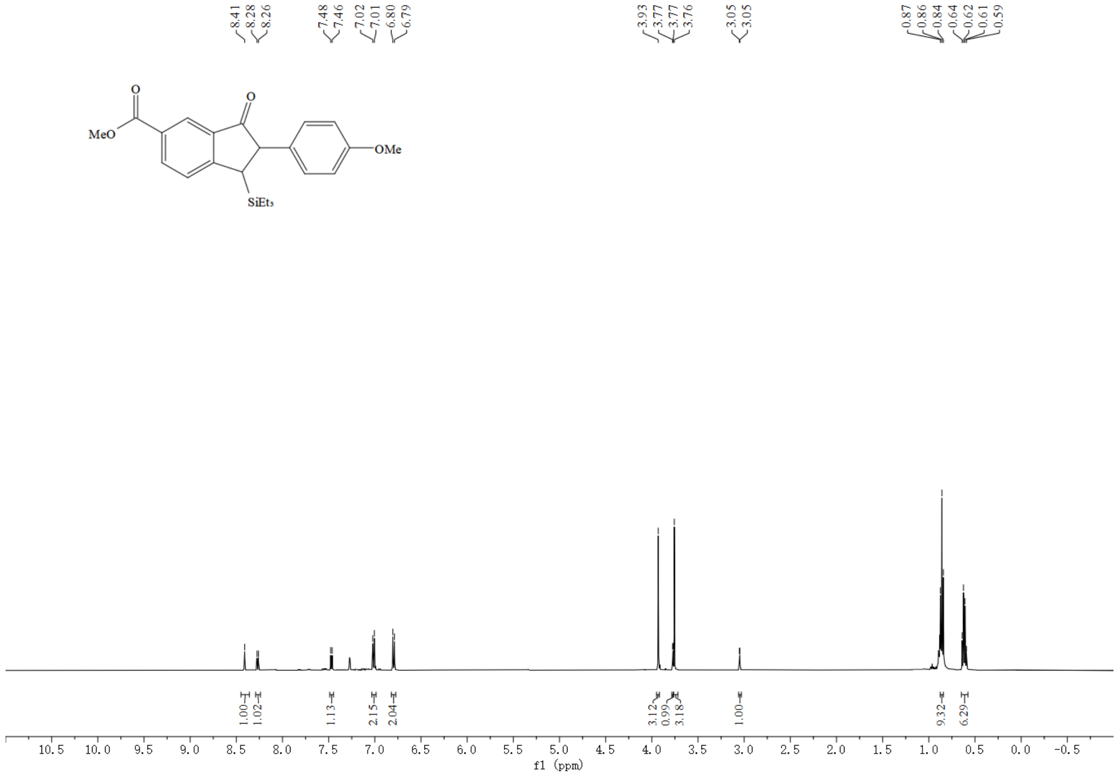

^{13}C{^{1}H} NMR (126 MHz) Spectrum of **3ar** in $CDCl_3$

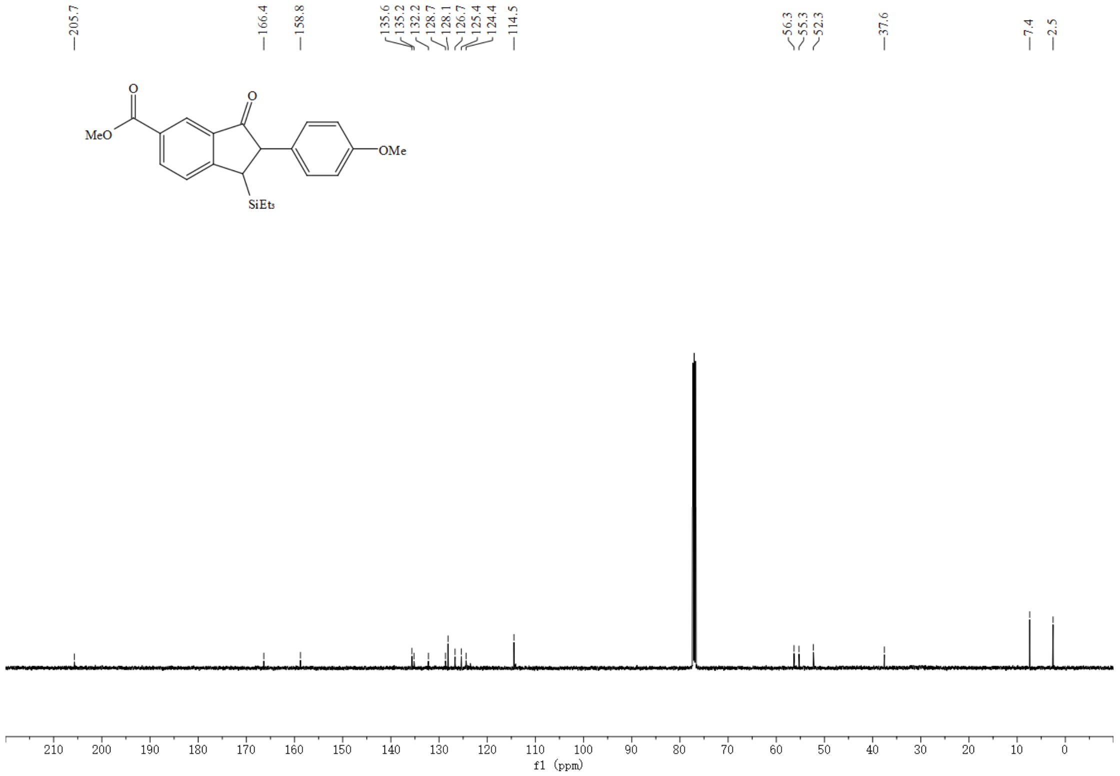

^{1}H NMR (500 MHz) Spectrum of **3as** in $CDCl_3$

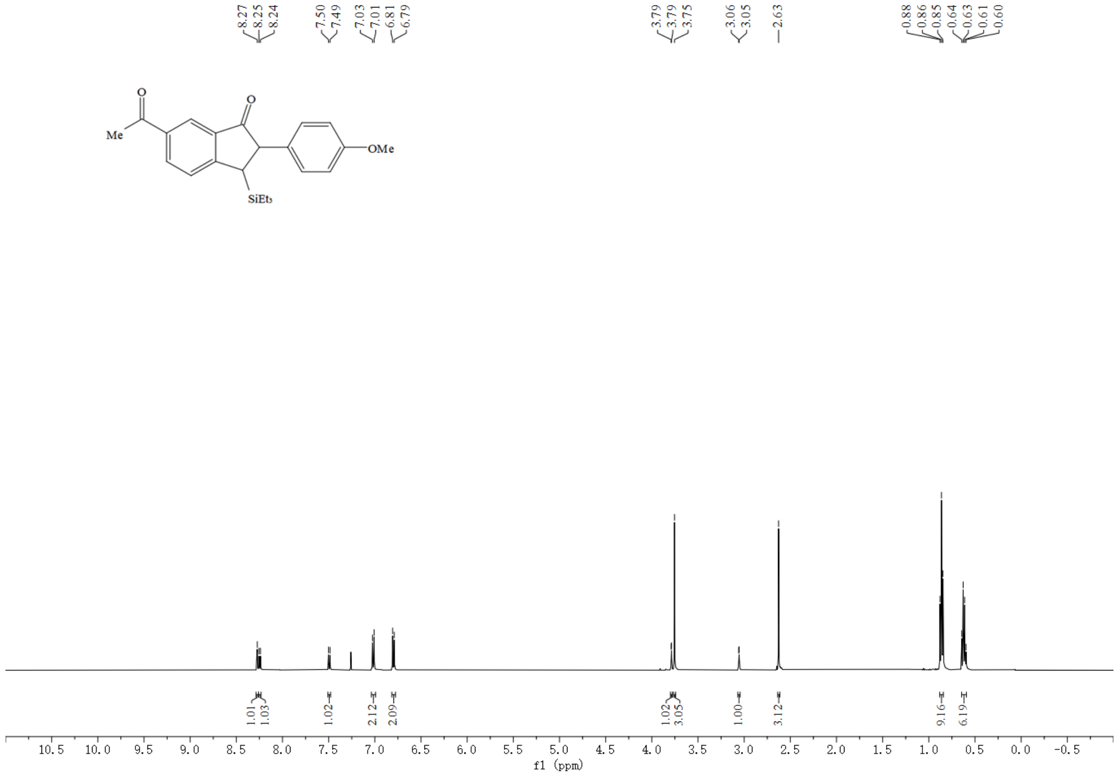

^{13}C{^{1}H} NMR (126 MHz) Spectrum of **3as** in $CDCl_3$

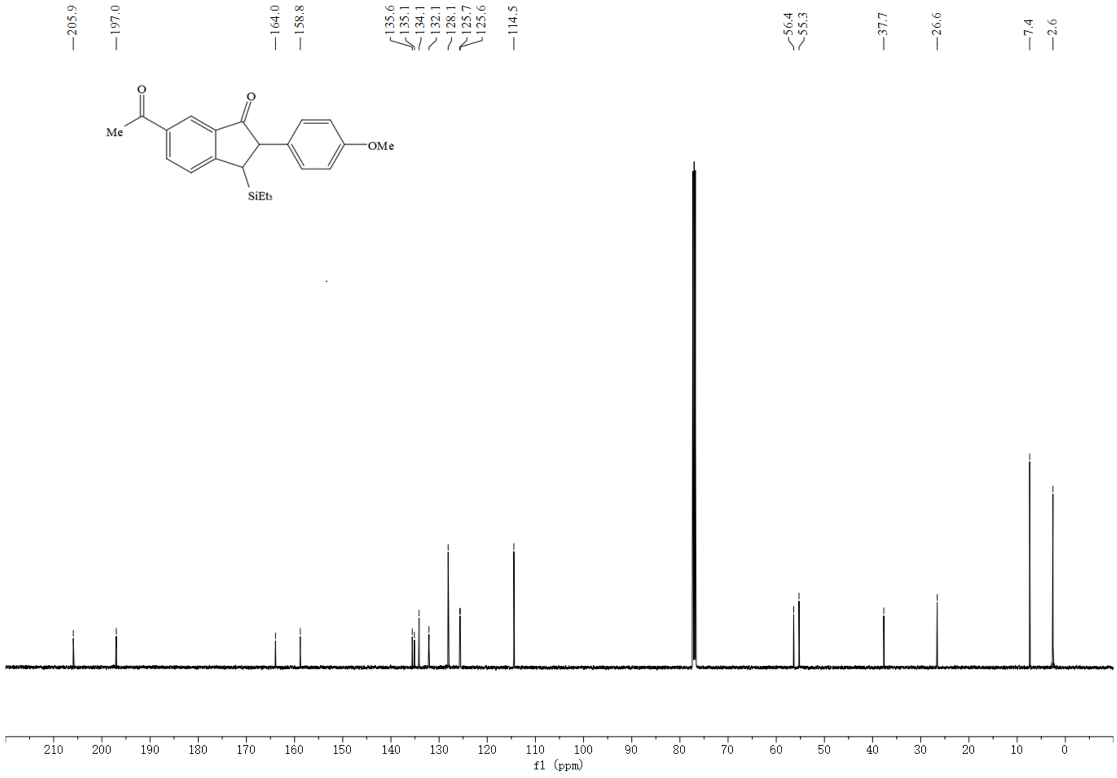

^{1}H NMR (500 MHz) Spectrum of **3at** in $CDCl_3$

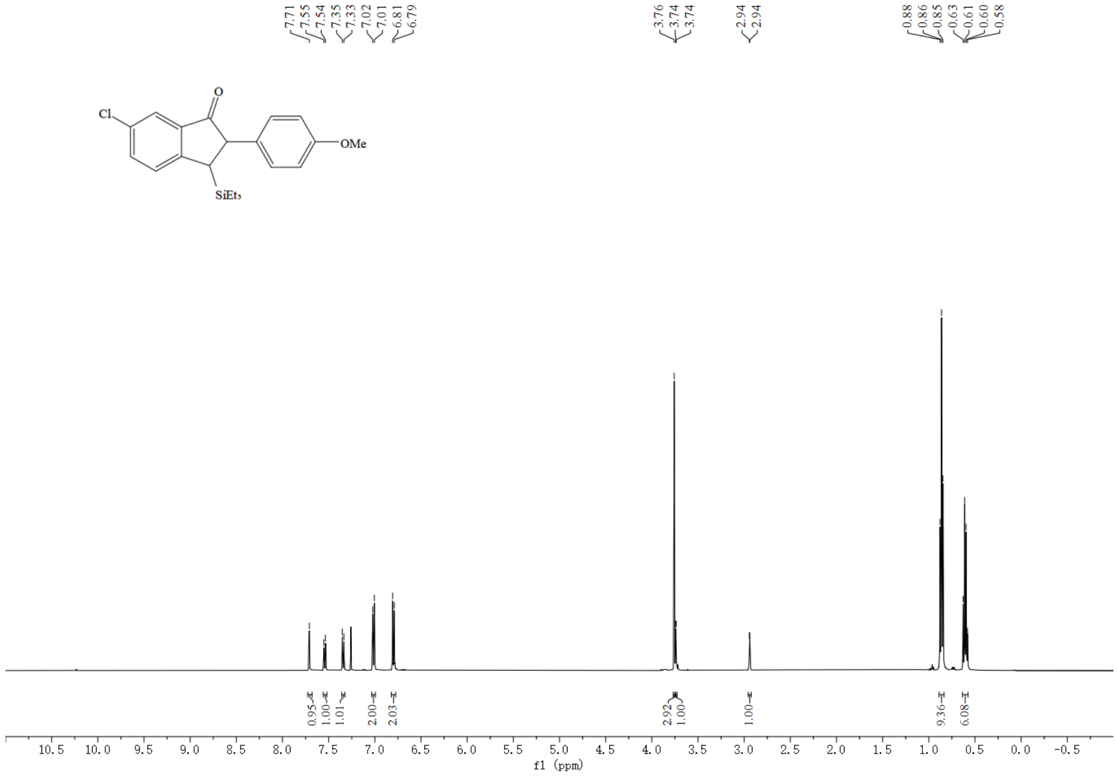

^{13}C{^{1}H} NMR (126 MHz) Spectrum of **3at** in $CDCl_3$

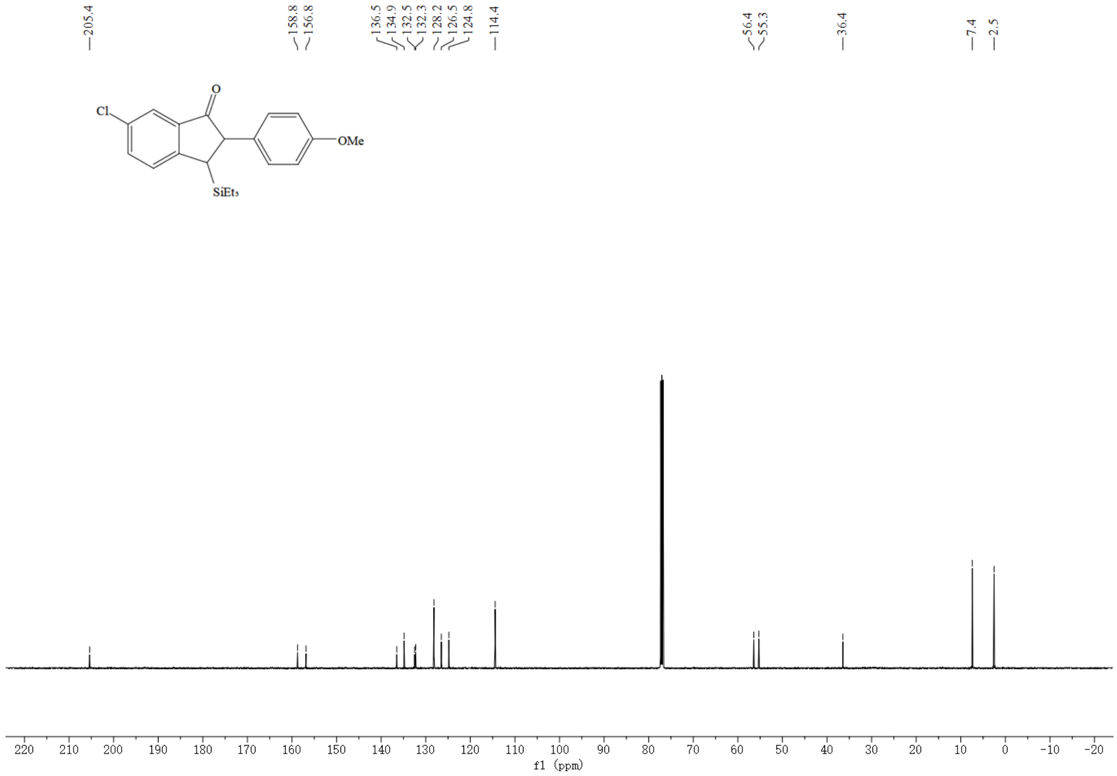

^{1}H NMR (500 MHz) Spectrum of **3ba** in $CDCl_3$

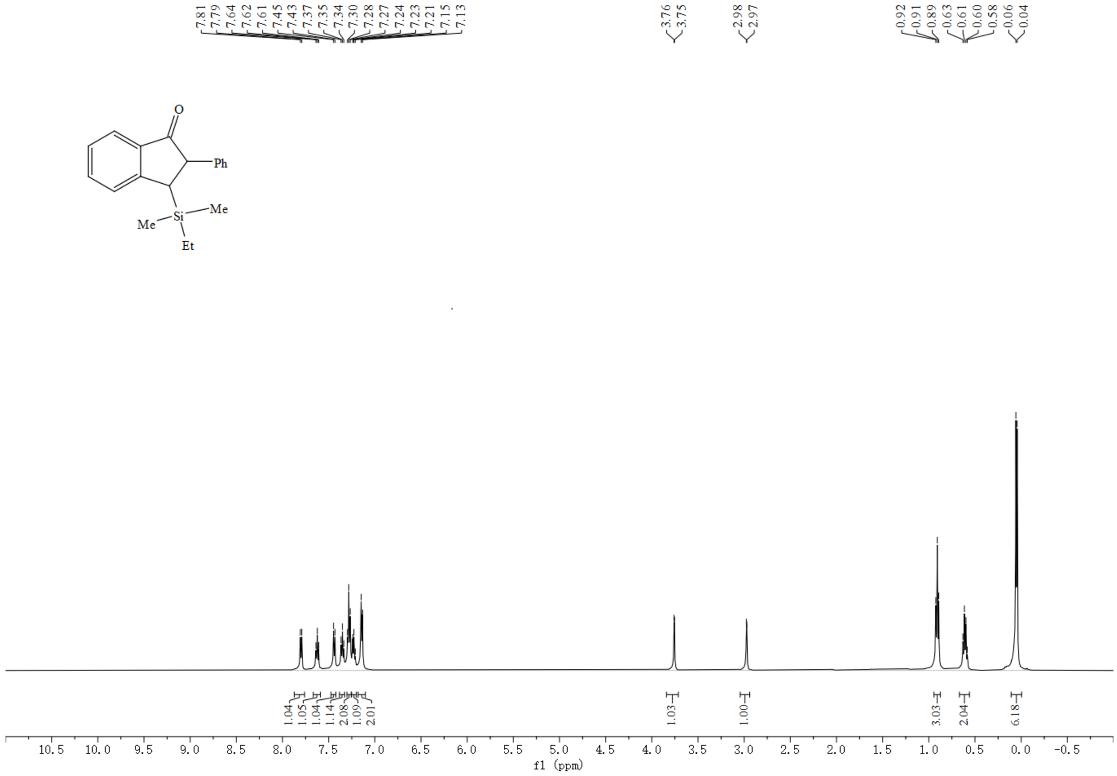

^{13}C{^{1}H} NMR (126 MHz) Spectrum of **3ba** in $CDCl_3$

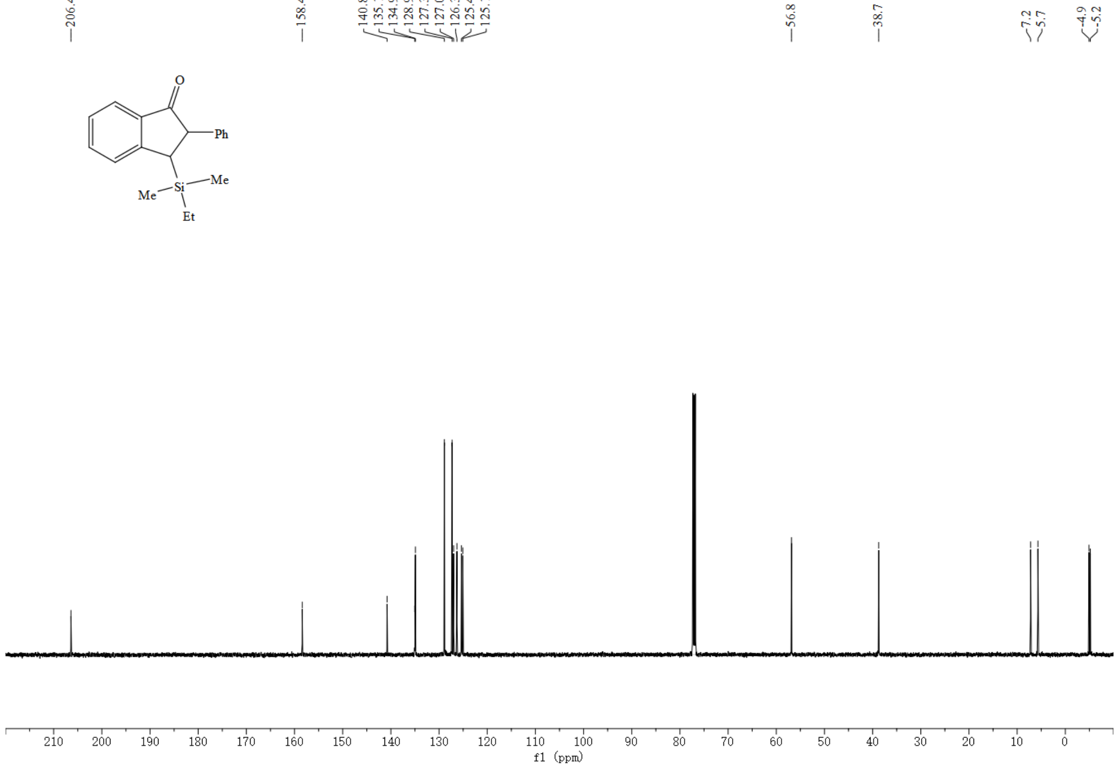

^{1}H NMR (500 MHz) Spectrum of **3ca** in $CDCl_3$

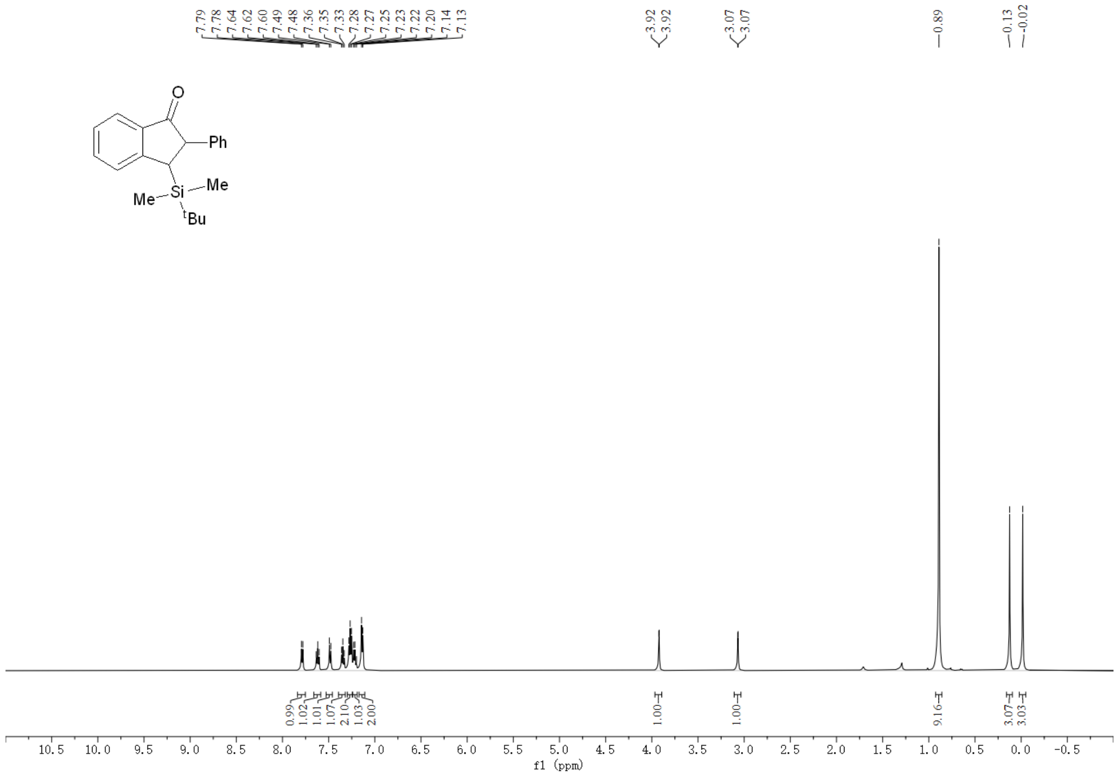

^{13}C{^{1}H} NMR (126 MHz) Spectrum of **3ca** in $CDCl_3$

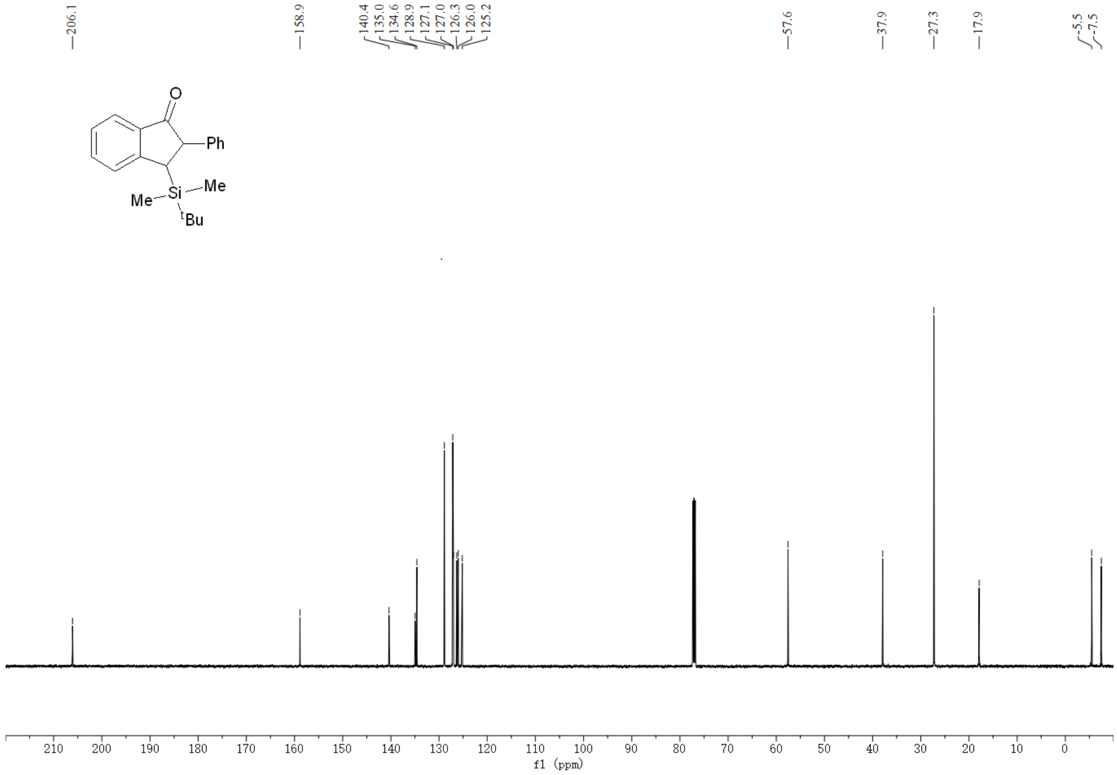

^{1}H NMR (500 MHz) Spectrum of **3da** in $CDCl_3$

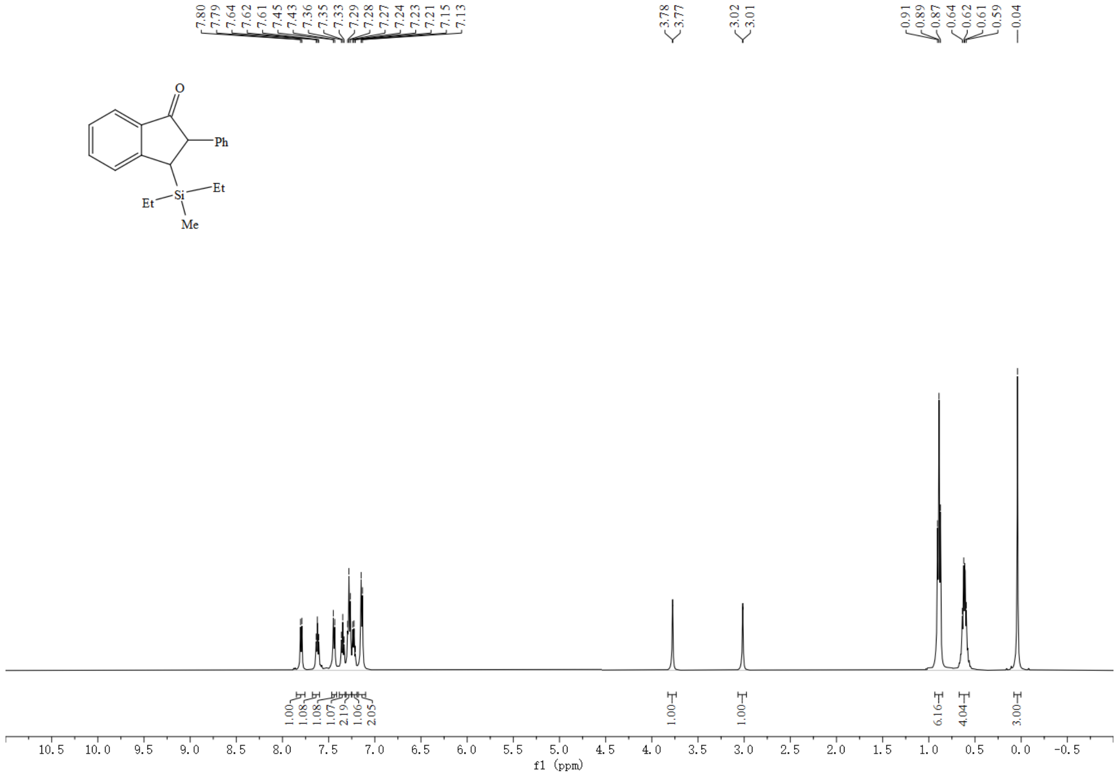

^{13}C{^{1}H} NMR (126 MHz) Spectrum of **3da** in $CDCl_3$

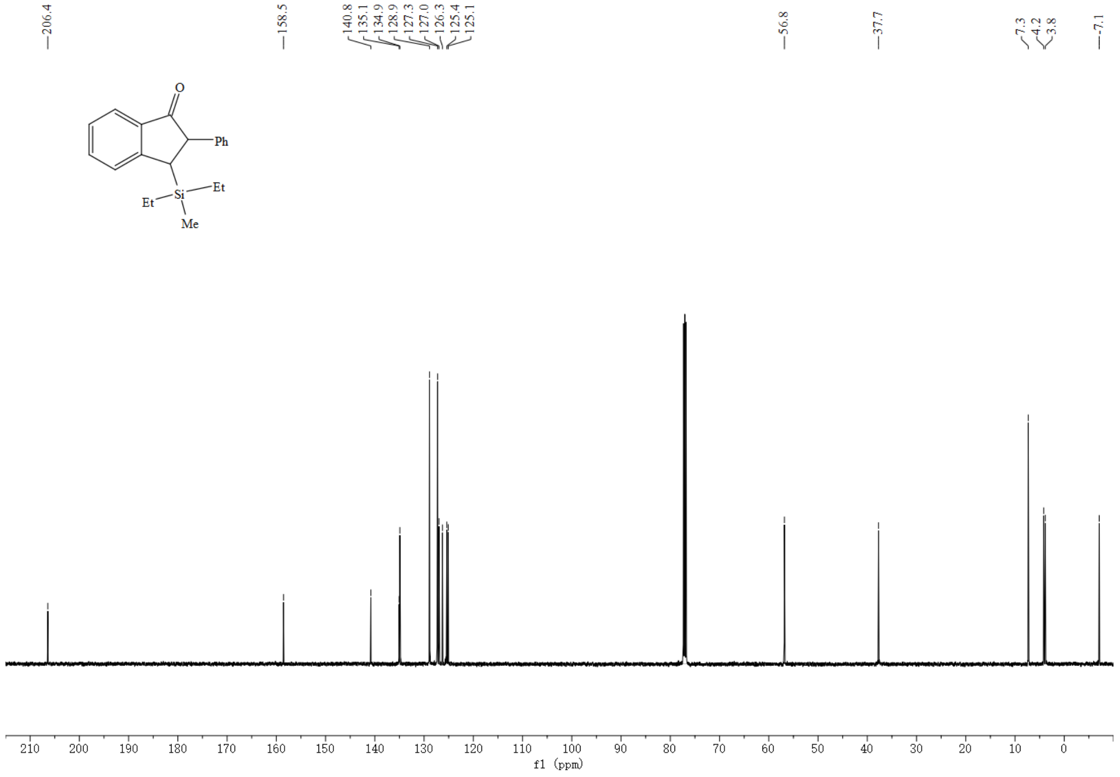

^{1}H NMR (500 MHz) Spectrum of **3ea** in $CDCl_3$

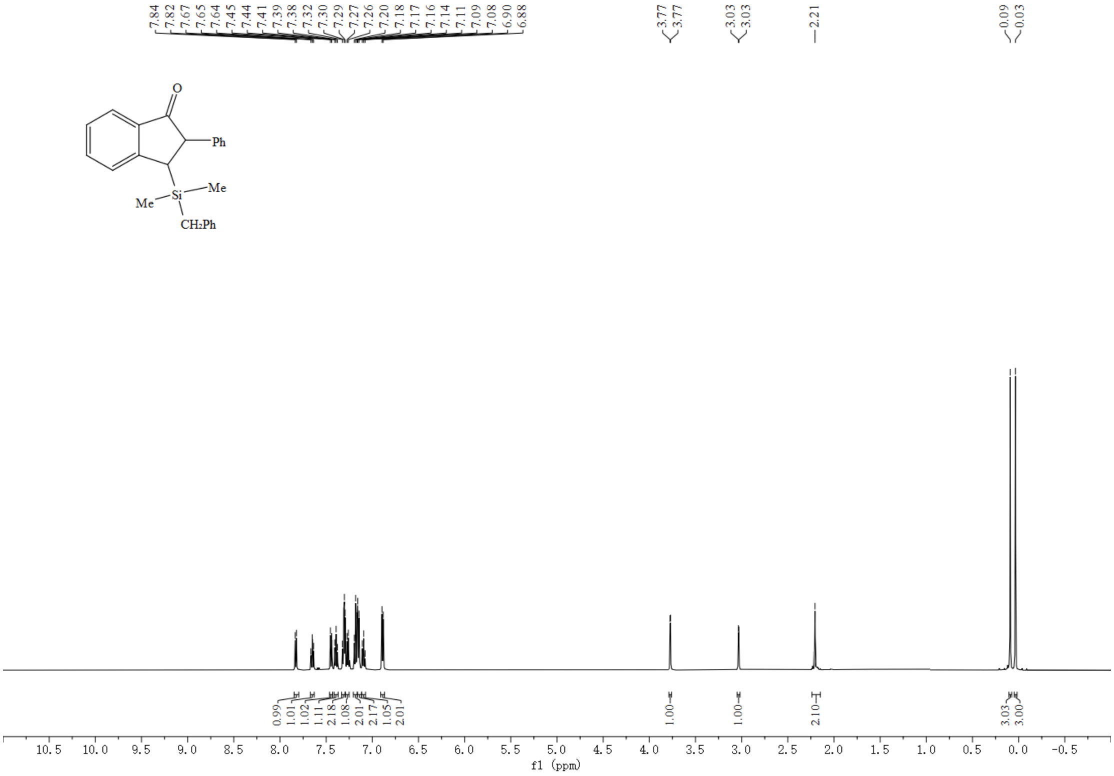

^{13}C{^{1}H} NMR (126 MHz) Spectrum of **3ea** in $CDCl_3$

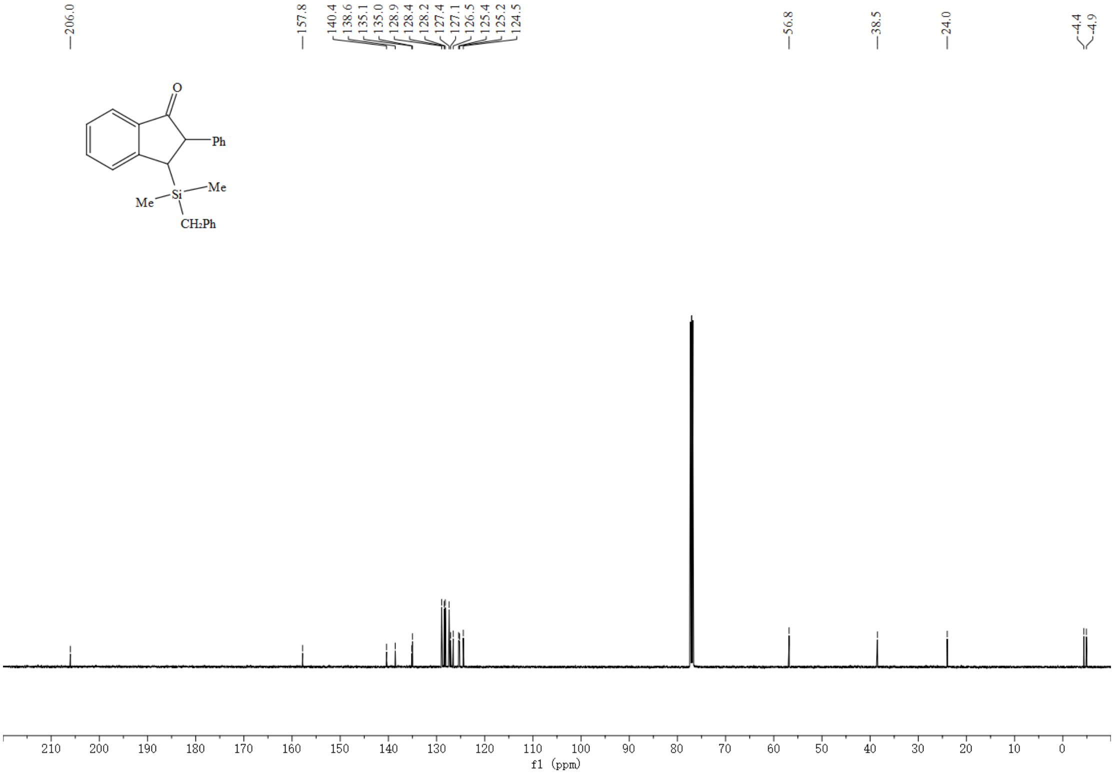

^{1}H NMR (600 MHz) Spectrum of **3fa** in $CDCl_3$

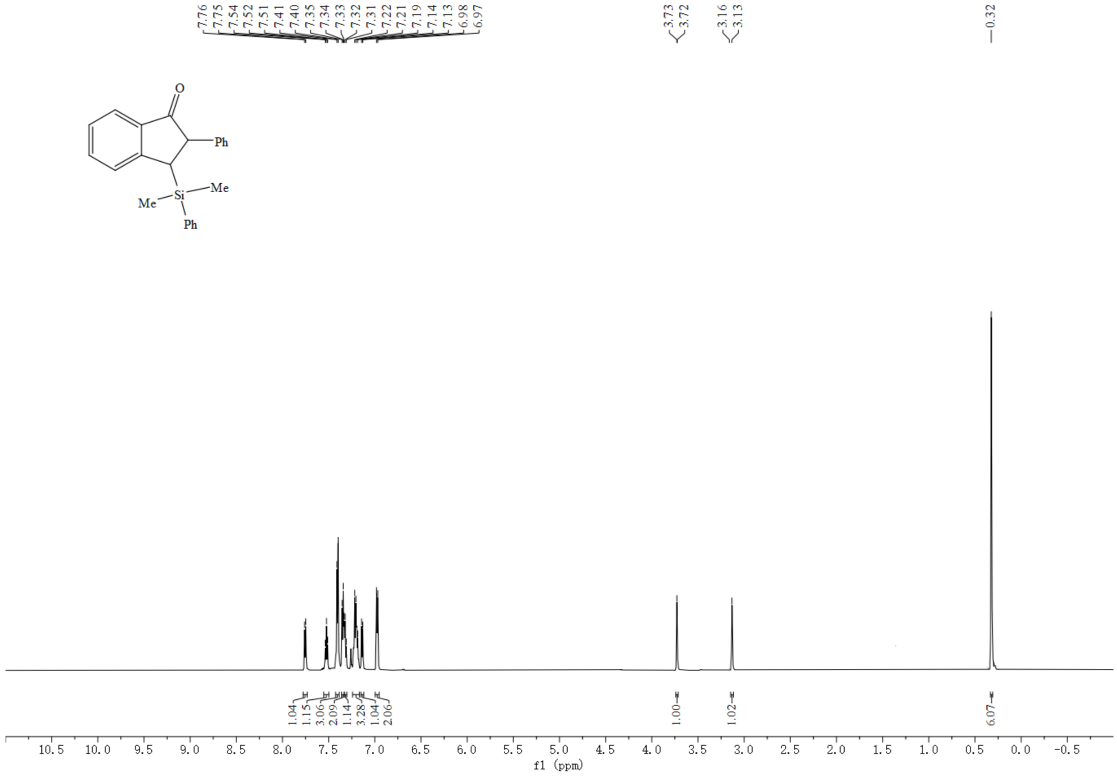

^{13}C{^{1}H} NMR (151 MHz) Spectrum of **3fa** in $CDCl_3$

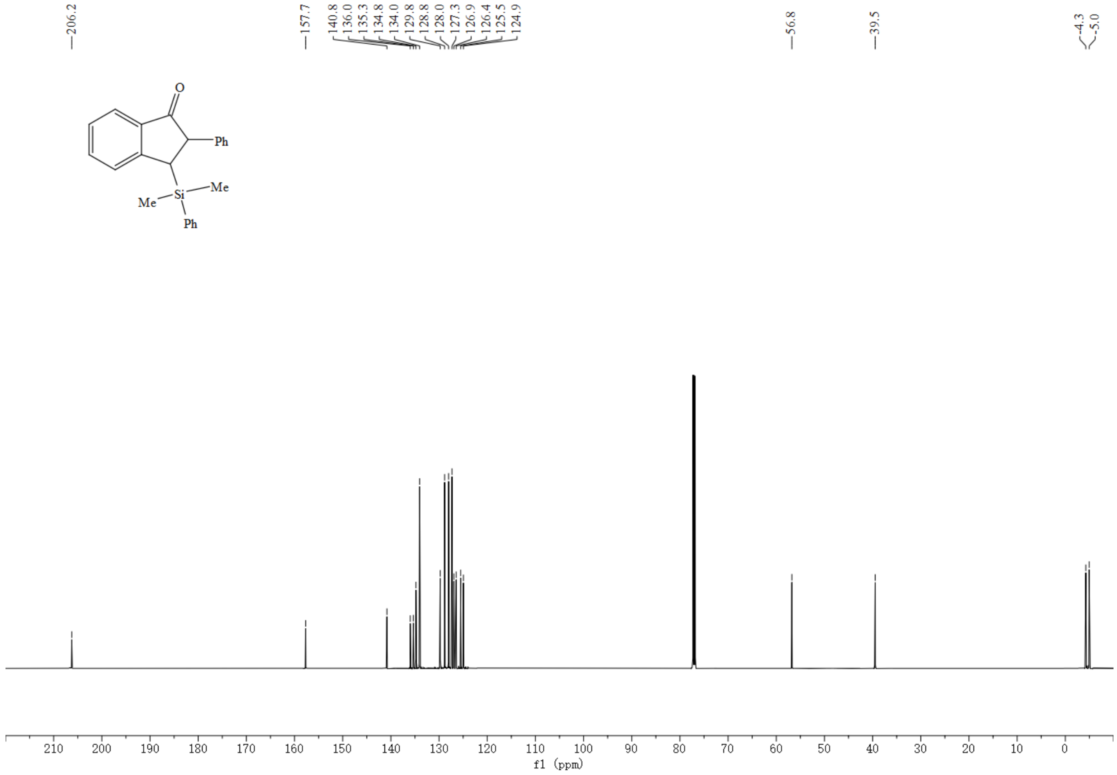

^{1}H NMR (500 MHz) Spectrum of **3ga** in $CDCl_3$

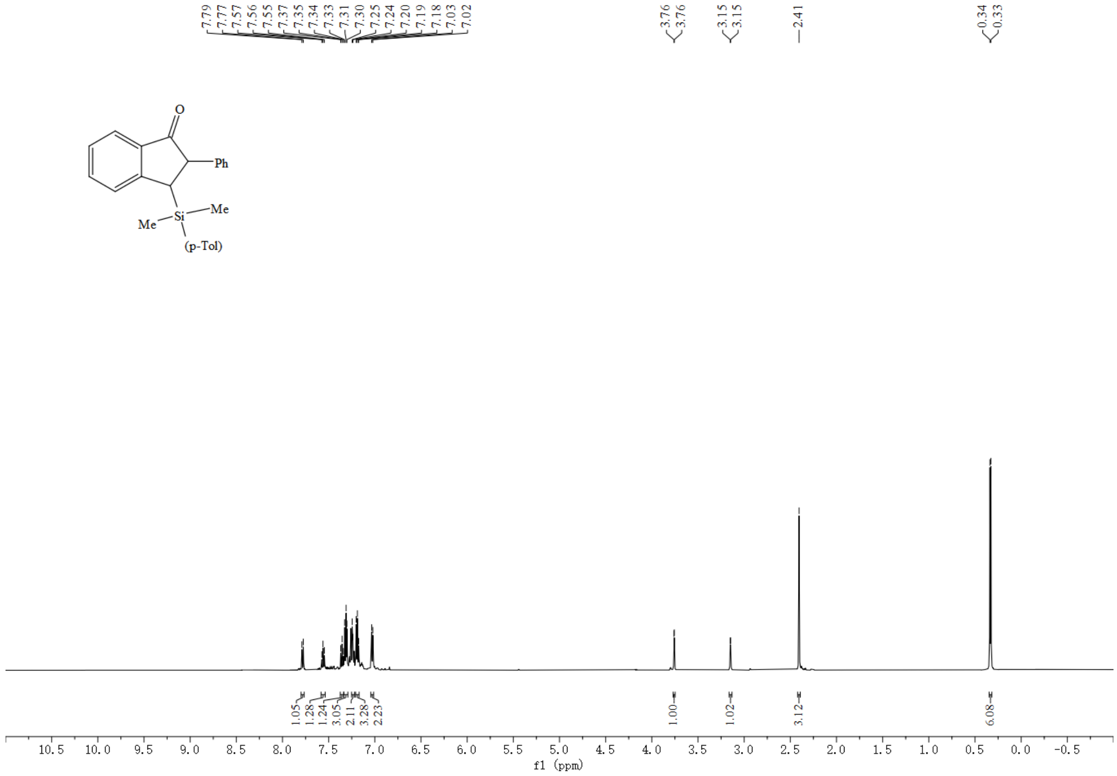

^{13}C{^{1}H} NMR (126 MHz) Spectrum of **3ga** in $CDCl_{3S}$

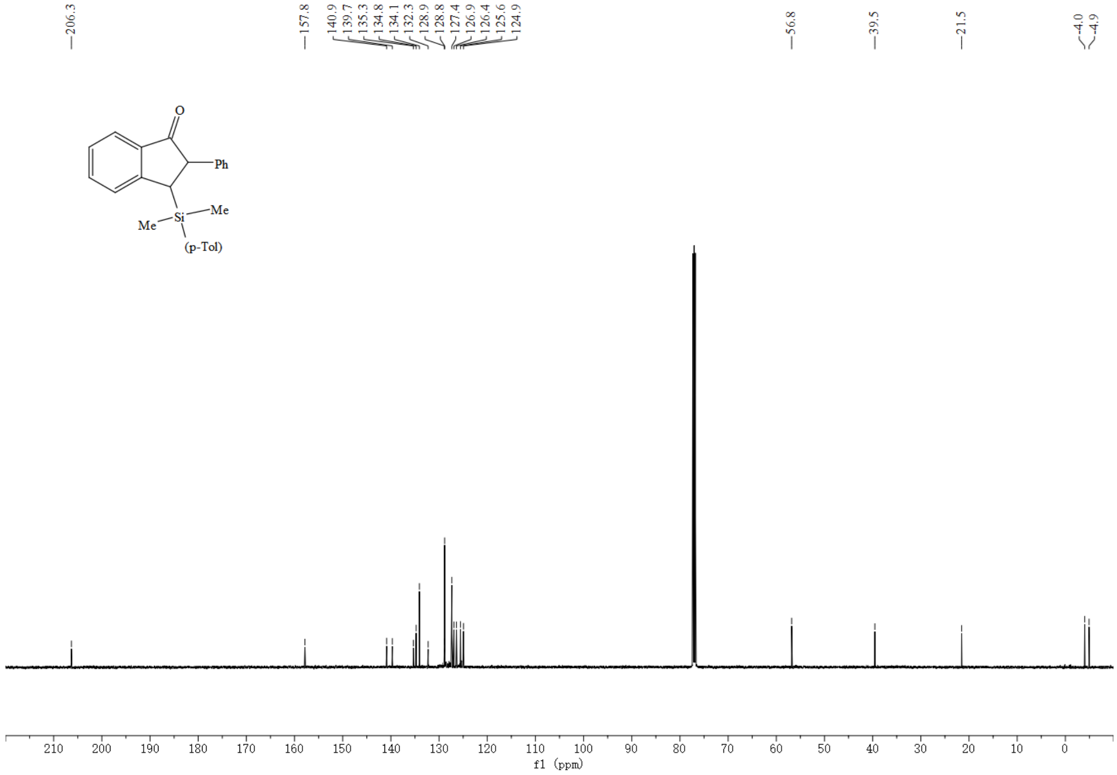

^{1}H NMR (500 MHz) Spectrum of **3ha** in $CDCl_3$

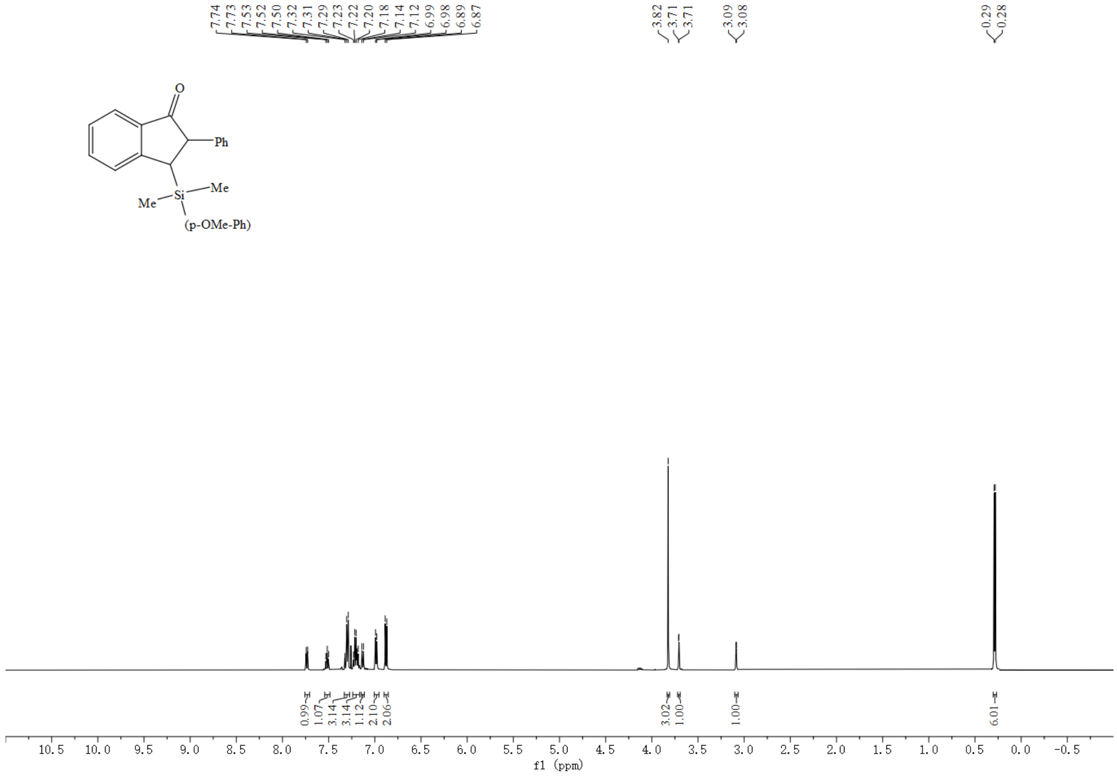

$^{13}C\{^{1}H\}$ NMR (126 MHz) Spectrum of **3ha** in $CDCl_3$

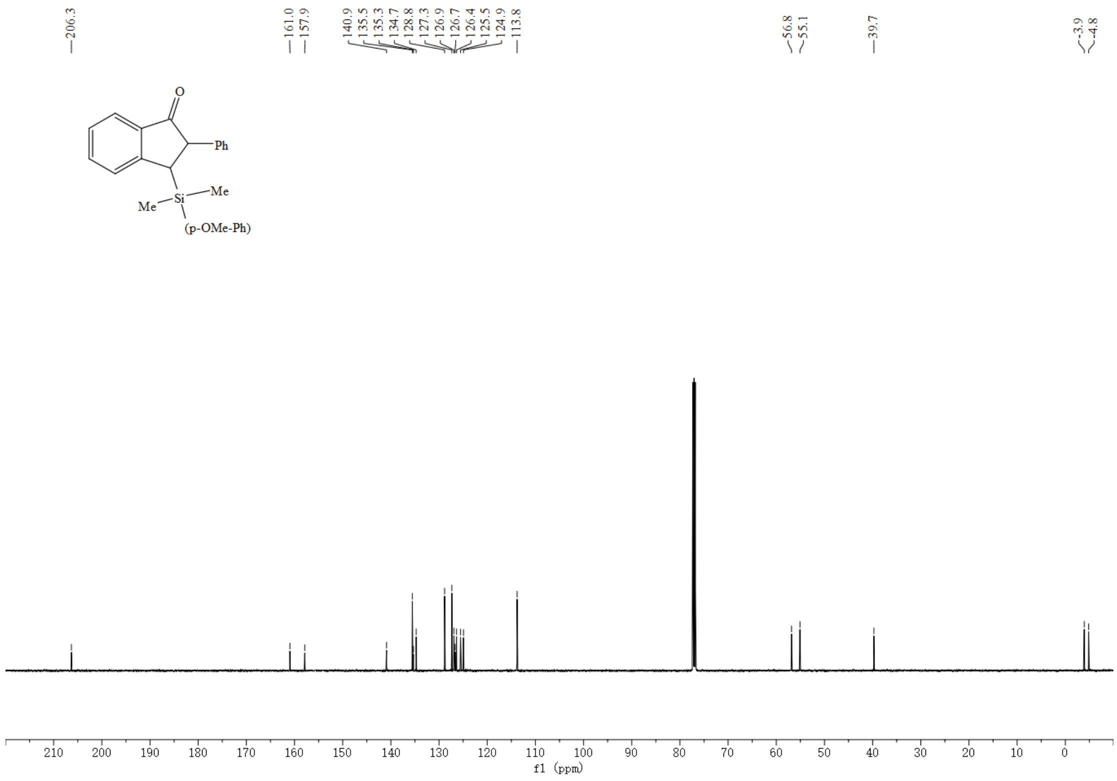

^{1}H NMR (500 MHz) Spectrum of **3ia** in $CDCl_3$

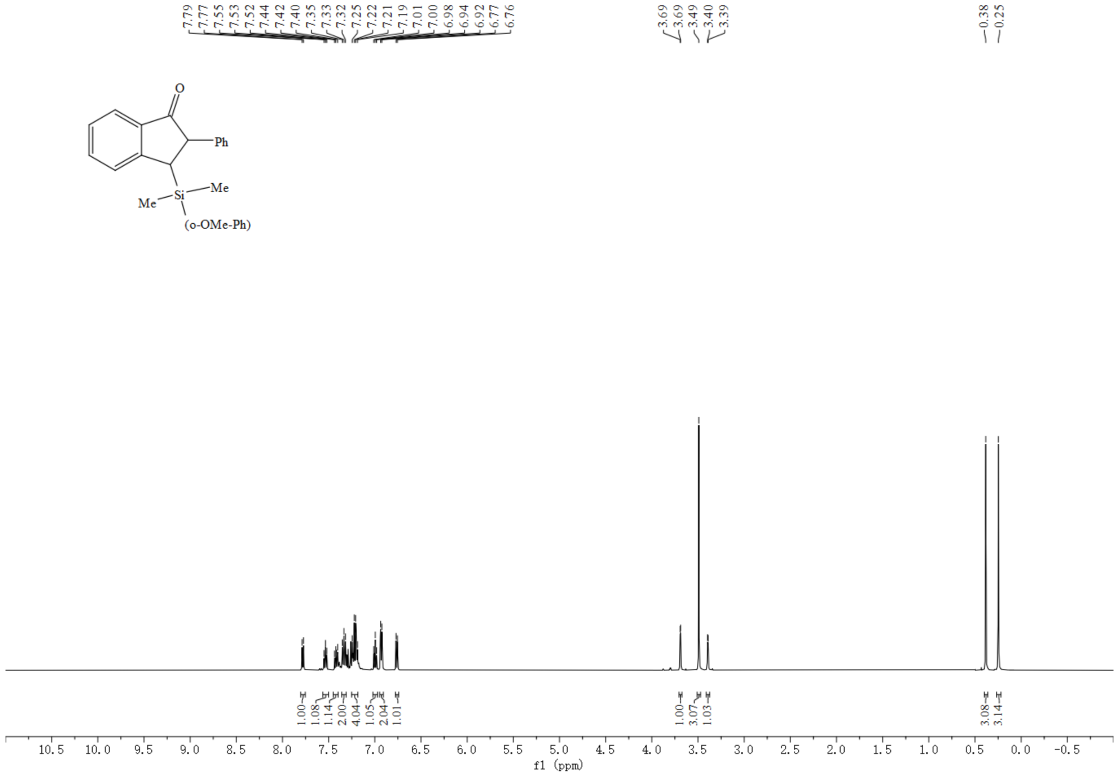

^{13}C{^{1}H} NMR (126 MHz) Spectrum of **3ia** in $CDCl_3$

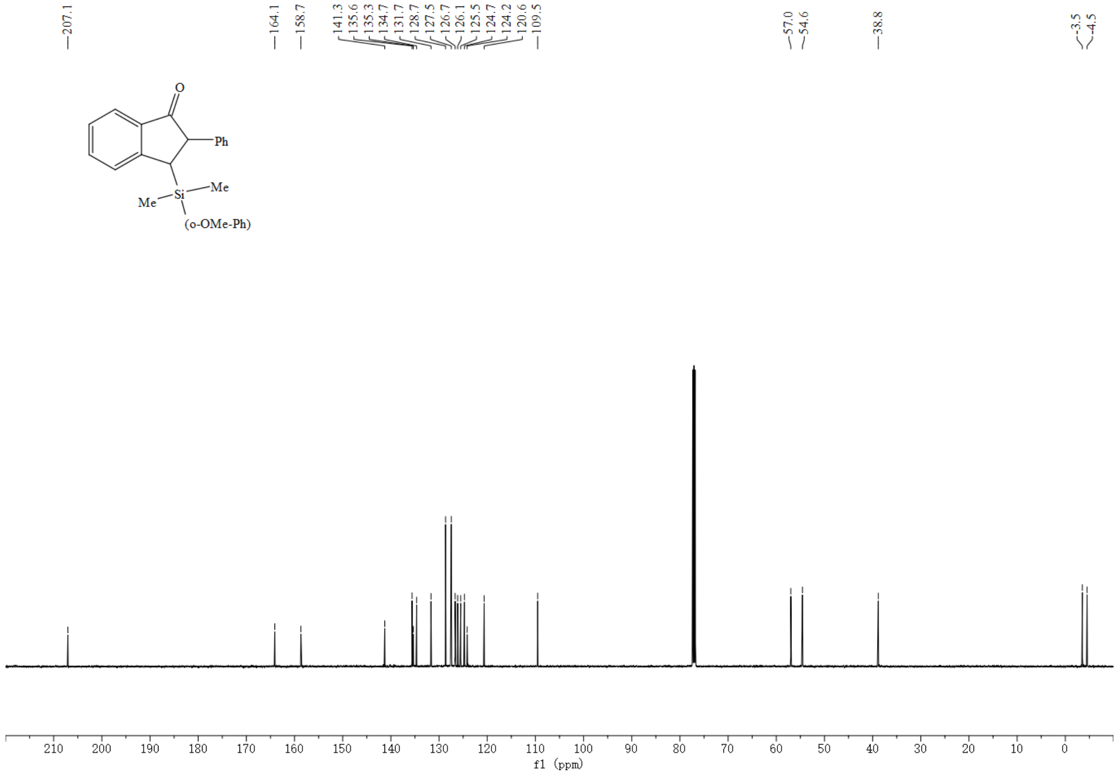

^{1}H NMR (400 MHz) Spectrum of **3ja** in $CDCl_3$

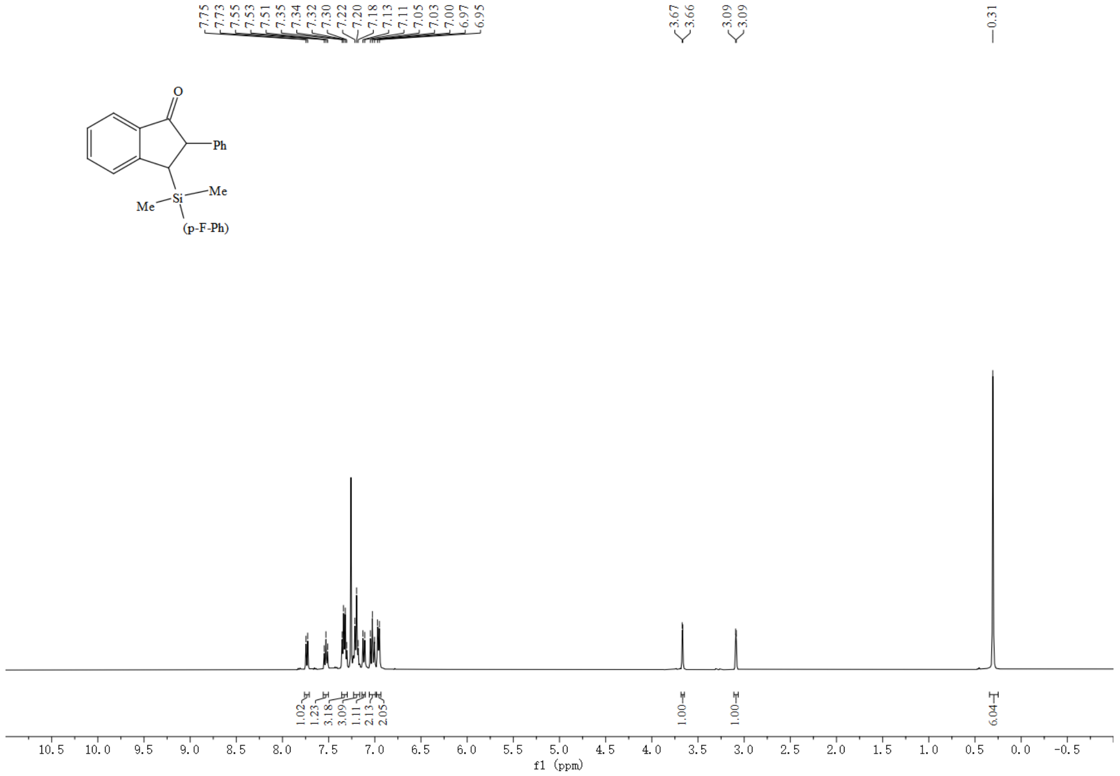

^{13}C{^{1}H} NMR (101 MHz) Spectrum of **3ja** in $CDCl_3$

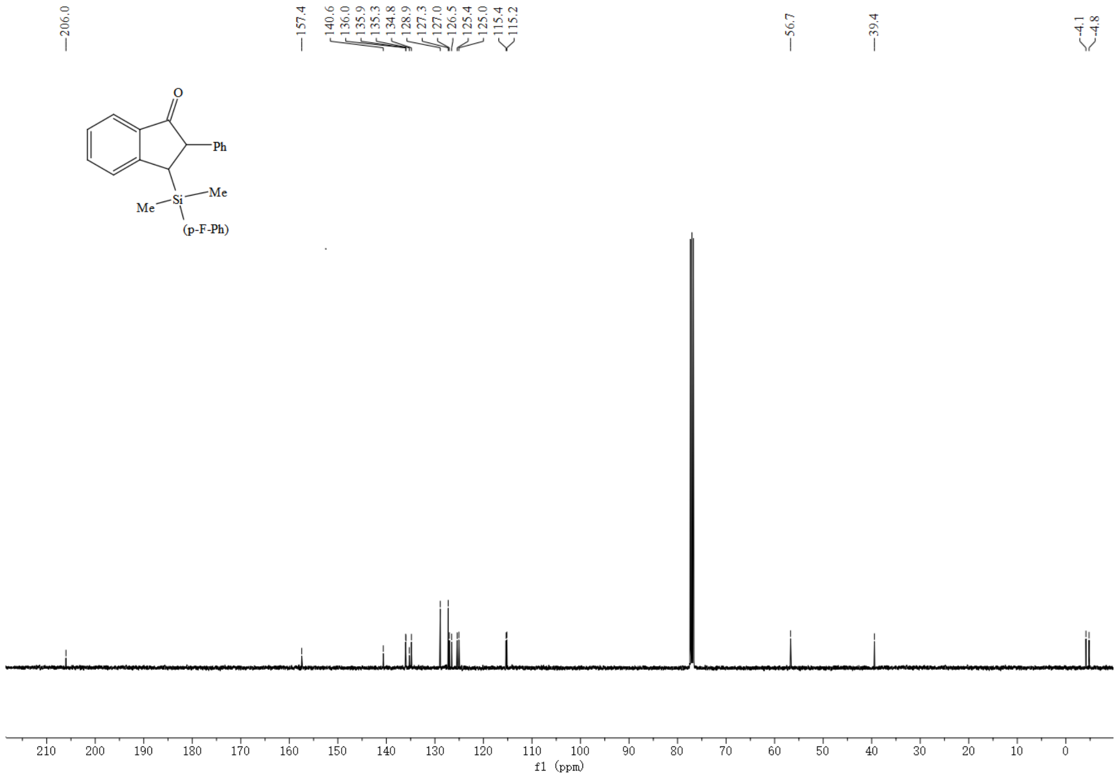

^{19}F NMR (376 MHz) Spectrum of **3ja** in $CDCl_3$

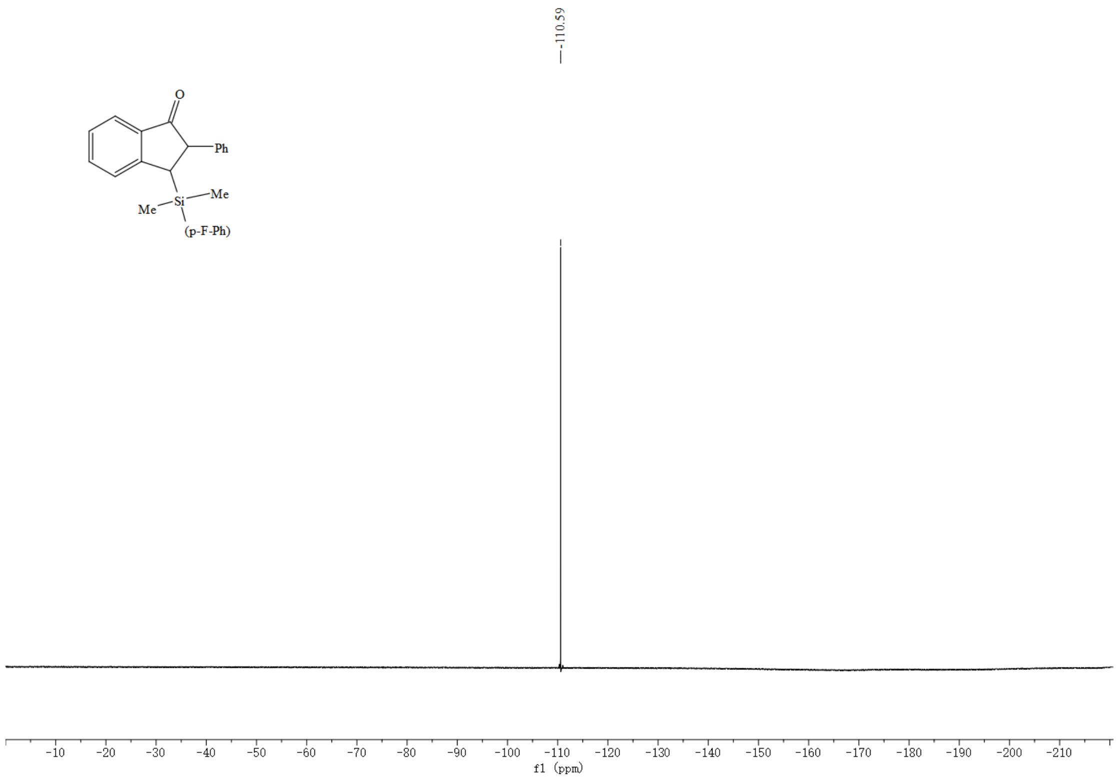

^{1}H NMR (600 MHz) Spectrum of **3ka** in $CDCl_3$

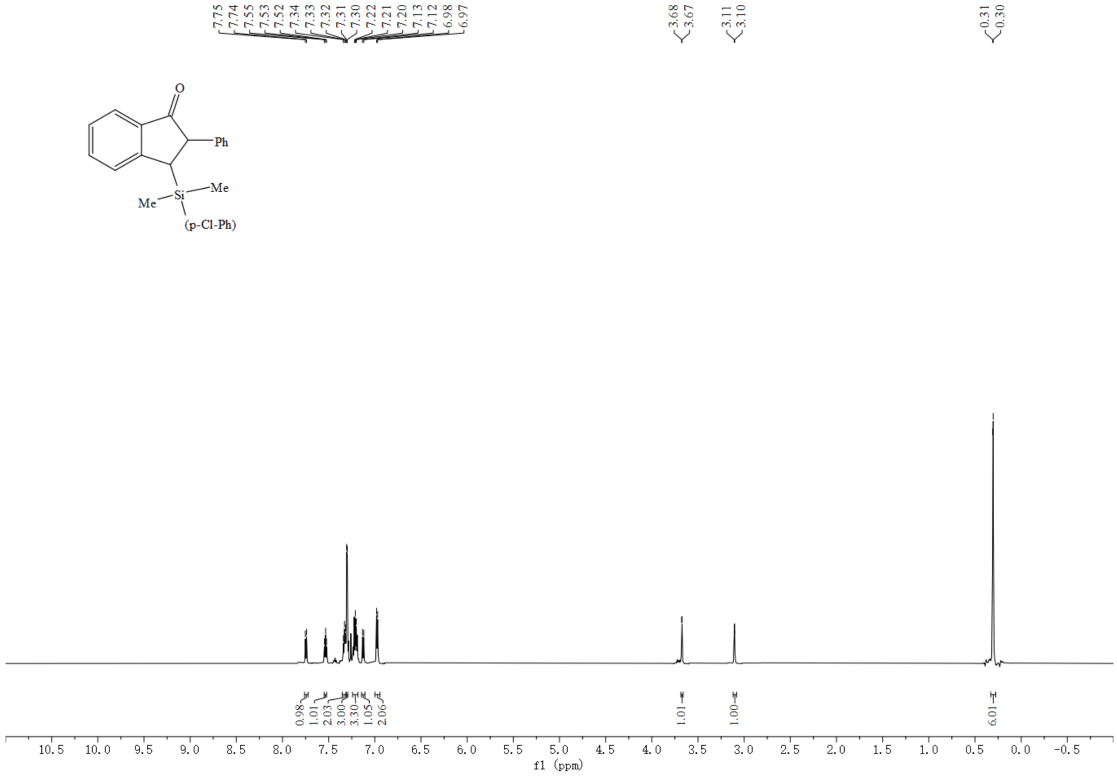

$^{13}C\{^{1}H\}$ NMR (151 MHz) Spectrum of **3ka** in $CDCl_3$

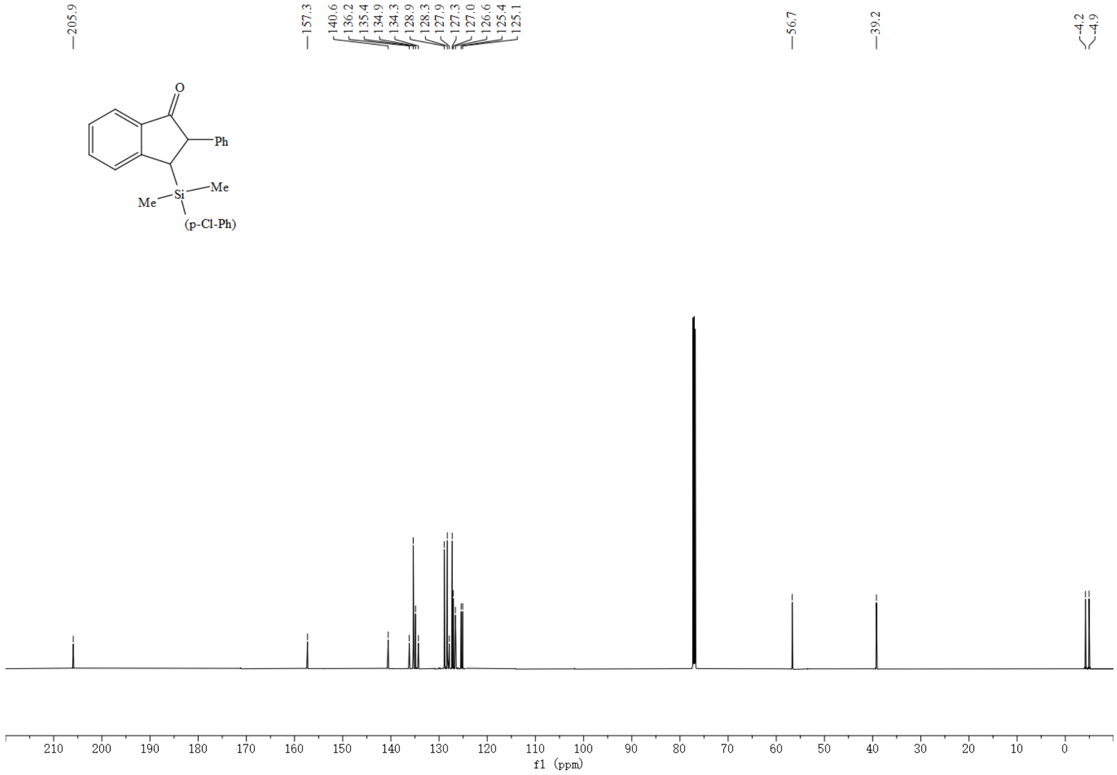

^{1}H NMR (500 MHz) Spectrum of **3aa+[D]-3aa** in $CDCl_3$

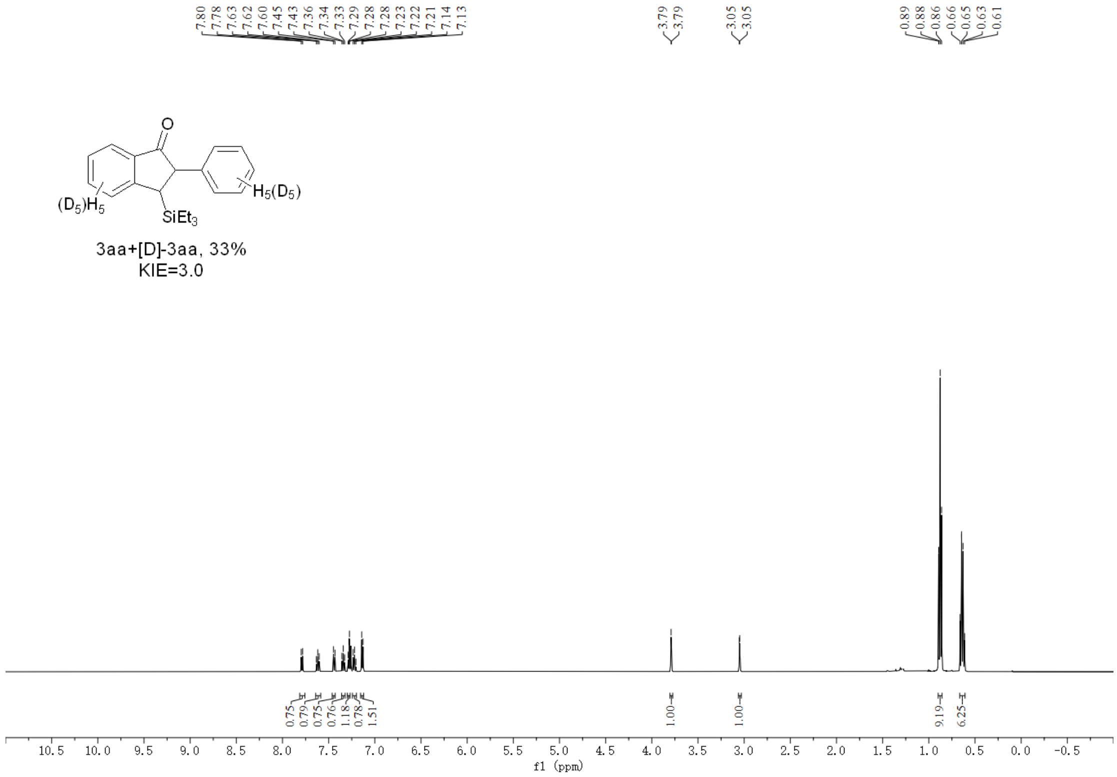

^{1}H NMR (500 MHz) Spectrum of **[D]-3aa** in $CDCl_3$

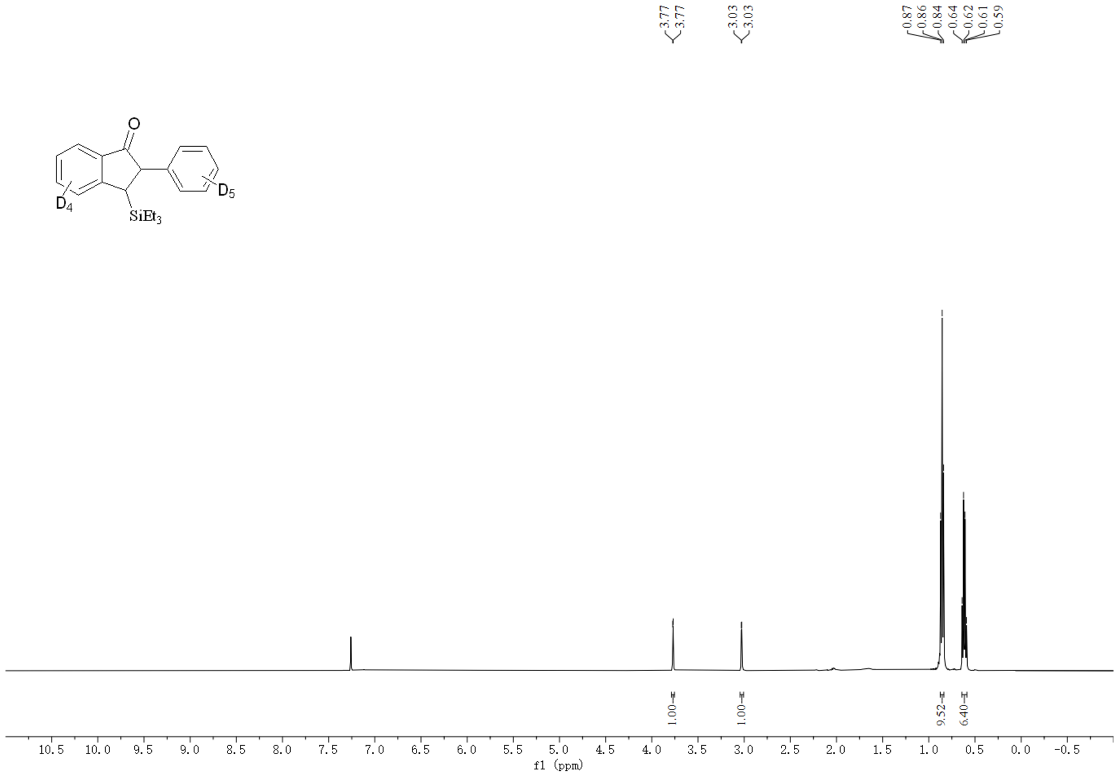

Supplement: Supplementary file 1 [file ol6c00345_si_001.pdf]
